# Acknowledgement to Reviewers of *International Journal of Molecular Science* in 2019

**DOI:** 10.3390/ijms21020668

**Published:** 2020-01-19

**Authors:** International Journal of Molecular Science Editorial Office

**Affiliations:** MDPI, St. Alban-Anlage 66, 4052 Basel, Switzerland

The editorial team greatly appreciates the reviewers who have dedicated their considerable time and expertise to the journal’s rigorous editorial process over the past 12 months, regardless of whether the papers are finally published or not. In 2019, a total of 6447 papers were published in the journal, with a median time to first decision of 16 days and a median time from submission to publication of 35 days. The editors would like to express their sincere gratitude to the following reviewers for their generous contribution in 2019:

Aalen, Reidunn BirgittaLoewen, MicheleAasen, TrondLogan, SreemathiAav, RiinaLogarinho, ElsaAbad-Zapatero, CelerinoLoh, KimAbarrategi, AnderLoh, ZhixuanAbassi, ZaidLohr, ChristianAbate, GiuliaLoinard, CélineAbbate, AntonioLoizides-Mangold, UrsulaAbbina, SrinivasLojkić, MartinaAbbott, DavidLoka, DimitraAbdalla, Maher Y.Lombardi, AngelaAbdallah, AliLombardi, TommasoAbdelalim, Essam M.Lombardi, Vincent C.Abdel-Daim, MohamedLomonaco, TommasoAbdelrahman, MostafaLonardo, AmedeoAbdelrazik, HebaLončar, Mirela BausAbduo, JaafarLondei, PaolaAbebe, WorkuLong, YichengAbend, MichaelLong, YuchenAbeti, RosellaLonguespee, RemiAbeywardana, TharindumalaŁoniewski, IgorAbinaya, ManivannanLönnberg, TuomasAblonczy, ZsoltLooijenga, Leendert H.J.Abondio, PaoloLopaschuk, Gary D.Abraham, Bincy P.Lopes, FranciscaAbraham, DietmarLópez Antón, RicardoAbraham, EleniLópez Lluch, GuillermoAbraham, NaderLopez, Jose JavierAbraham, Samuel JKLópez, Susana B BravoAbrol, RavinderLópez-Cebral, RitaAbrunhosa, LuísLópez-Cornejo, PilarAbu Farhan, Abdal RahmanLopez-Ferber, MiguelAbu-Reidah, Ibrahim M.López-Guerrero, José AntonioAburima, AhmedLópez-Hoyos, MarcosAbu-Romman, SaeidLopez-Jornet, PiaAbuyassin, Bisher H.López-López, DanielAccardi, LuisaLópez-López, JoséAccardo, GraziaLópez-López, ManuelAceto, SerenaLopez-Viñas, EduardoAcher, Francine C.Lopresti, FrancescoAchim, CristinaLops, FrancescoAchreja, AbhinavLora, JorgeAcuña-Castroviejo, DaríoLorber, AvrahamÁdám, Attila L.Lorberboum-Galski, HayaAdamakis, Ioannis-DimosthenisLordan, RonanAdamcakova-Dodd, AndreaLorentz, AxelAdamczak, MarekLorenz, AlexanderAdamczyk, BartoszLorenz, KristinaAdamek, AgnieszkaLorenzen, MarceAdamowski, MaciekLorenzo González, ÓscarAdams, BrianLorico, AurelioAdams, JamesLortholary, AlainAdamska-Patruno, EdytaLortzing, TobiasAdegbola, Samuel O.Losada, Juan MAdela Iuga, CristinaLosada-Perez, PatriciaAdell, TeresaLosurdo, GiuseppeAdhikari, AtinŁotocka, BarbaraAdhikari, NeetaLötsch, JörnAdhikari, RajanLotti, Lavinia VittoriaAdhikary, AmitavaLou, ChenguangAdi, Pradeepkiran JangampalliLou, SongAdly, Frady G.Loussouarn, MargotAdriana, Estrada-BernalLovicu, FrankAdunyah, Samuel E.Lovisolo, ClaudioAfantitis, AntreasLöw, PéterAffolter, AnnetteLowin, TorstenAfonin, KirillLozano, R. M.Afonso, JulietaLozano-Ojalvo, DanielAfzal, AdeelLu, Da-WenAgamennone, MariangelaLu, HuaAgarwal, PoonamLu, Jeng-WeiAgarwal, SaurabhLu, JingAgarwal, SumitLu, Jyh-FengAgarwala, SeemaLu, LingengAggarwal, Bharat B.Lu, QuanlongAggeli, Ioanna-KaterinaLu, ShaoyongAgirre, JonLu, SizhaoAgnelli, LucaLu, Wan-JungAgnieszka, SzopaLu, Wei-YuAgostino, MarkLu, YouAgrimi, GennaroLu, YujingAguilera-Aguirre, LeopoldoLu, ZhanpingAh Kim, HyunLuca, AndreiAhlenstiel, ChantelleLucacchini, AntonioAhluwalia, AmritaLucaciu, Constantin MihaiAhmad, AliLucarini, SimoneAhmad, FahimLucas, Andrew T.Ahmad, KhurshidLucas, DonaldAhmad, ShakilLucas, Jose AntonioAhmed, Aisha SiddiqahLucas, KurtAhmed, ChulbulLucek, KayAhmed, HafizLuchini, ClaudioAhmed, IshfaqLuciani, GiuseppinaAhmed, MukhtarLuciani, PaolaAhmed, ZeeshanLuciano, Alberto MariaAhmetaj-Shala, BlerinaLučić, BonoAhn, TaehoLucie, GrodeckáAhola, AnttiLucini, CarlaAi, YongLuco, Reini FAiba, YuichiroŁuczak, JustynaAiello, FrancescaLudlow, Andrew T.Aiolfi, RobertoLuduena, RichardAjduk, AnnaLudwiczuk, AgnieszkaAkagi, SatoshiLuhová, LenkaAkahoshi, MitsuteruLui, Edmund M.K.Akamizu, TakashiLui, KathyAkbar, MohammedLukas, JanAkerman, IldemLukaszewski, Adam J.Akhtar, SultanLukinavicius, GrazvydasAkiba, YasutadaŁukowski, AdrianAkimov, Sergey A.Lukyanov, Konstantin A.Akita, SadanoriLumniczky, KatalinAkk, GustavLuna, Jesus IsaacAkula, ShawLundberg, MarieAlaimo, AlessandroLundell, TainaAlain, LescoatLunov, OlegAlain, TommyLuo, JianAl-Ajrash, Saja M. NabatLuo, Jiang-YunAlam, Mohammad ParvezLuo, RayAlambert, Renata PereiraLuo, WeiboAl-Aubaidy, HayderLuo, XuAlazzam, AnasLuo, ZonghuaAlba, JuanLuongo, LivioAlbano, EmanueleLupberger, JoachimAlbano, FrancescoLupini, AntonioAlbarnaz, J. D.Lupu, StelianAlbertin, GiovannaLuque, Victor ManuelAlbertini, GiuliaLuquet, DelphineAlbertolle, Matthew E.Lurje, GeorgAlbizua, IgorLuscinskas, Francis William (Bill)Albonici, LoredanaLusczek, Elizabeth R.Alboresi, AlessandroLushchekina, SofyaAlbrecht, JanLushington, GerryAlcendor, Donald J.Lusk, C. PatrickAlcolea, VeronicaLutfy, KabirullahAlder, JanetLutter, ErikaAldewachi, HasanLutz, ManfredAldieri, ElisabettaLuvisetto, SiroAldred, KatieLuwor, RodneyAldred, NicholasLux, Robert L.Alduina, RosaLy, PeterAl-Dujaili, EmadLymperopoulos, AnastasiosAleixandre, ManuelLyons, LisaAleksandrov, AlexeyLyukmanova, Ekaterina N.Alessandri, ClaudiaM Kamal, Abu HenaAlessandri, GiulioM. Laura, IddaAlessenko, AliceM’kacher, RadhiaAlessenko, Alice V.M’Koma, Amosy EAlesutan, IoanaMa, BuyongAleu, JosefinaMa, ChuyingAlexaki, Vassilia IsminiMa, GuojiaAlexander, DorotheaMa, HongAlexander, H. DenisMa, LiangAlexander, RebeccaMa, YiyiAlexandre, AnaMa, ZhikunAlexandre, MezentsevMaak, Steffen MaakAlexandrov, Oleg S.Maász, GáborAlexeyev, MikhailMaayah, Zaid H.Alexiou, ChristophMacauley, Matthew S.Alexov, EmilMaccallini, CristinaAlfano, DanielaMacchi, BeatriceAlfano, MassimoMacDiarmid, Colin W.Alfarouk, Khalid OmerMacDonald, Clinton C.Alferink, JudithMacDonald, JessicaAlfieri, CarloMacedo, FatimaAlfieri, CarolineMacedo, M. PaulaAlfonso-Prieto, MercedesMacgregor, RobertAlgate, KentMachelart, ArnaudAlhakamy, NabilMachida, ShuichiALHENC, FrancoisMachin, Daniel R.Al-Horani, Rami A.Macias, Maria J.Ali Zaidi, ShanMacias-Gonzalez, ManuelAli, HydarMaciążek-Jurczyk, MałgorzataAli, MuhammadMaciejczyk, MateuszAlicia, PickrellMacIejewska, DominikaAlicja, Macko-PodgorniMacín, Stella MarisAliev, GjumrakchMacIntosh, Gustavo C.Alina, WiszniewskaMacIver, M. BruceAlique, MatildeMacKenzie, AlasdairAlivernini, StefanoMackenzie, AndrewAlkasalias, TwanaMackenzie, SallyAllain, FabriceMackereth, CameronAllegaert, KarelMaclellan, Reid A.Allegra, SarahMacpherson, James N.Allendes, RodolfoMacQueen, AliceAlmeida, AfonsoMacreadie, IanAlmeida, HenriqueMadala, Hanumantha RaoAlmeida, LuisMadaro, LucaAlmeida, PedroMaddelein, Marie-LiseAlmoazen, HassenMadej, DawidAl-Mrabeh, AhmadMadej, M. GregorAlmstrup, KristianMaden Wilkinson, ThomasAloisi, Anna MariaMadine, JillianAlonso-Alconada, DanielMadkour, AichaAloraifi, FatimaMadonov, PavelAlpizar, Yeranddy AMadrigal, IreneAlqudah, AhmedMadsen, Claus KroghAlsaab, HashemMadureira, Patrícia A.Alt, KarenMadzhidov, TimurAlteri, ClaudiaMaeda, AkikoAltmann, BrigitteMaedler, KathrinAltmann, FriedrichMaehara, NoritoshiAltomare, ClaudiaMaekawa, MasamitsuAlva, NormaMaekawa, RyoAlvarez De La Rosa, DiegoMaffei, MargheritaAlvarez, GuzmánMagán-Fernández, AntonioAlvarez, Juan B.Magarlamov, Timur Yu.Álvarez, MarMaggi, DavideAlvarez-Pizarro, Juan CarlosMaggio, AlbinoÁlvarez-Sánchez, NuriaMagherini, FrancescaAlvarez-Suarez, JoseMaghuly, FatemehÁlvarez-Suarez, José M.Magierowski, MarcinAlves, CelsoMagini, AlessandroAlves, MarcoMagistrato, AlessandraAlves, SandraMaglio, MelaniaAlves-Lopes, RheureMagnaldo, ThierryAlviano, FrancescoMagnelli, LuciaAlyass, AkramMagni, FulvioAmantini, ConsueloMagnus, MarcinAmarilla, Alberto A.Magnusson, Karl-EricAmarowicz, RyszardMagnusson, NilsAmasheh, SalahMaher, PamelaAmato, AntonellaMahfouz, RedaAmato, FeliceMahmood, KashifAmbros, PeterMahmood, KhalidAmbu, RitaMahmood, NasirAmedei, AmedeoMahmuda, Naila AlAmelio, DanielaMahony, Timothy JohnAmengual, JaumeMai, Hans-JörgAmeye, MaartenMaia, CláudioAmiard, SimonMaia, F. RaquelAmillis, SotirisMaidaniuc, AndreeaAmin, RajeshMaier, PatrickAminin, Dmitry L.Maioli, MargheritaAmiral, JeanMaiorano, DomenicoAmith, Schammim RayMaisch, TimAmmazzalorso, AlessandraMaity, ShuvadeepAmoaku, Winfried M.Maixent, Jean MichelAmodeo, PietroMájek, PavelAmodio, NicolaMajello, BarbaraAmorim, Maria JoaoMajhi, PrabinAmoutzias, GrigoriosMajima, Hideyuki J.Ampofo, EmmanuelMajka, MarcinAmpollini, LucaMajkowska-Skrobek, GrażynaAmrani, AbdelazizMajor, FrançoisAmrani, YassineMajor, Ian T.An, HongMajumdar, Adhip P. N.An, SeungMajumdar, IndrajitAn, YuMajumder, AvisekAna, Fernández-OcañaMajumder, MousumiAnakk, SayeepriyadarshiniMajumder, MrinmoyeeAnamthathmakula, PrashanthMajumder, PoulamiAnand, SwatiMak, AnselmAnastasiadou, MariaMakarevich, PavelAncona-Contreras, VeronicaMakarevitch, IrinaAndersen, Thomas LevinMakarska-Bialokoz, MagdalenaAnderson, CharlesMakena, Monish RamAnderson, Jeffrey L.Maki, JamesAnderson, Sarah NMaki, YasuyukiAndia, IsabelMakishima, MakotoAndjelkovic, Anuska V.Malacarne, GiuliaAndo, HitoshiMalacrinò, AntoninoAndrada, DiegoMalaguarnera, GiuliaAndrade, FernandoMalaguarnera, MicheleAndre, HelderMalanga, GerardAndreadou, IoannaMalany, SiobhanAndreas, ProkeschMalara, AlessandroAndreassi, MariaMalard, VeroniqueAndreev, Yaroslav A.Malashicheva, AnnaAndré-Fontaine, GenevieveMalatino, LorenzoAndreotti, GiuseppinaMalavasi, VeronicaAndres, Allen M.Malavolta, MarcoAndrés-León, EduardoMalekigorji, MaryamAndreuzzi, EvaMalerba, GiovanniAndrikopoulos, PetrosMali, VishalAnestopoulos, IoannisMalic, SladjanaAng, Kok SiongMalik, Anna R.Ang, WeeMalik, RavinderAng, ZhiweiMalinowski, RobertAngeles-Shim, Rosalyn B.Malkowski, EugeniuszAngeletti, MauroMallamace, DomenicoAngeli, FabioMallamace, FrancescoAngelici, Maria CristinaMallat, MichelAngelini, FrancescoMallein-Gérin, FrédéricAngell, YvonneMalliavin, ThérèseAngelova, AngelinaMallipeddi, Prema LathaAngelova, MihaelaMallipeddi, SrikrishnanAngelucci, AdrianoMallis, PanagiotisAngius, FabrizioMallo, NataliaAniello, FrancescoMalmström, LarsAnifandis, GeorgeMalpeli, GiorgioAnisimova, IrinaMaltseva, ArinaAnken, RalfMalwal, Satish R.Annese, VitoMaly, PavelAnnesley, SarahMammadova-Bach, ElminaAnnibalini, GiosuèMan, AdrianAnquetil, VincentManagò, StefanoAnsari, RaisManai, FedericoAntal, DianaManakhov, AntonAntcliffe, DavidManali, EffrosyniAnticoli, SimonaManariotis, Ioannis D.Antinozzi, CristinaMaňásková-Postlerová, PavlaAntipova, VeronicaManavalan, BalachandranAntognelli, CinziaManavski, NikolayAntoine, Marie-HélèneManca, GabrieleAntoine, RodolpheManca, Maria LetiziaAntonini, DarioMancheño, JoséAntonkiewicz, JacekMancias, Joseph D.Antony, AntoniouMancinelli, RominaAntony, JaneMancini, AnnamariaAntonyuk, SvetlanaMancini, FrancescaAntosiewicz, JędrzejMancini, GiordanoAntunović, MajaMancini, InesAnzenbacher, PavelMancini, ManuelaAoe, TomohikoMancini, Stéphane J. C.Aoki, DanManciulli, TommasoAon, Miguel A.Manco, GiuseppeAparicio Blanco, JuanMandal, Mihir KumarAparicio, FredericMandato, ClaudiaApartsin, EvgenyMandyam, ChitraApone, FabioManera, ClementinaAppathurai, Narayana P.Manetti, MirkoApplebaum, MarkMangala Prasad, VidyaAprile, AlessioMangalagiu, IonelAprile, Francesco A.Mangaonkar, AbhishekAprodu, IulianaMangini, GiacomoAquaro, StefanoMangogna, AlessandroAragonés, JuliánMankia, Kulveer S.Arai, RyoichiMankowski, MarkArai, ToshiroMann, FrancisArakawa, YukiMann, MatiAraki, RyoichiMann, Vivek Aramini, JamesMannell, HannaAran, GemmaMannik, JaanAranda, Carlos J.Manocha, GunjanAránega, AmeliaManocha, Gunjan DhawanAraniciu, CătălinManohar, NivedhArano, YasushiMansell, ThomasAraújo, WagnerMånsson, AlfArbo, IngeridMansuri, ShahidArbones-Mainar, JoseMantamadiotis, TheoArchambault, VincentMante, Francis K.Archid, RamiManti, LorenzoArcidiacono, BiagioManuel, RemyArdolino, MicheleManzotti, GloriaArena, FrancescaMao, MaoArena, GoffredoMao, MengArenas, JesúsMao, YoudongArendarczyk, NataliaMarasco, DanielaArenz, ChristophMarc, GabrielArevalo, Juan CarlosMarçais, AntoineArguelles, SandroMarceau, FrançoisArif, EhteshamMarcel R., De ZoeteArikkath, JyothiMarcel, VirginieArimura, ShinichiMarchese, AdrianoArino, JoaquinMarchese, MariaArita, MasanoriMarchesi, AlessandraArita, ReikoMarchetti, GiovannaAriyasu, DaisukeMarchetti, MartaAriyoshi, WataruMarchetti, SandrineAriz, IdoiaMarchi, Fabiola DeArkhipov, Vladimir I.Marchini, SergioArmanini, DecioMarchuk, Douglas A.Armato, UbaldoMarciello, MarziaArmellini, EliaMarcin Henryk, StruszczykArmenio, BarbosaMarciniec, KrzysztofArmijo, Juan FranciscoMarco, FranciscoArnaud, FrançoiseMarco-Contelles, JoséArndt, Patrick G.Marco-Contelles, Jose LuisArnesano, FabioMarcus, Schmitt-EgenolfArnold, RolandMarcuzzo, StefaniaAroca, RicardoMarczak, MałgorzataArora, GunjanMarec, FrantišekArora, MansiMarechal, AlexandreArosio, PaoloMareev, SemyonArrais, AldoMaresca, MarcArruda, JamesMaresca, VittoriaArruda, Valder RMargaglione, MaurizioArsene, Andreea LetiţiaMargiotta, AzzurraArtati, AnnaMari, MicheleArtero, RubenMarian, CatalinArtín-Aragón, Sagrario MMariani, ErminiaArumäe, UrmasMariani-Costantini, RenatoAryal, RishiMarie Camille, ChaumaisAryal, Uma KMariggiò, Maria A.Asa’ad, FarahMarigo, IlariaAsadi, AminMarigorta, Urko M.Asano, RyutaroMarijanovic, IngaAsano, YasuhisaMarin, TraciAsatryan, BabkenMarini, FrancescaAscenzioni, FiorentinaMariniello, LoredanaAscoli Marchetti, AndreaMarino, FrancaAsea, Alexzander A. A.Marino, JosephAsgharian, HosseinaliMarino, MariaAshfaq, Mohammad KhalidMarinozzi, MauraAshida, HitoshiMarins, MozartAshihara, EishiMariot, VirginieAshley, RyanMariotto, SofiaAshton, TrentMariscal, VicenteAsim, MohammadMarjanović, MarkoAsimaki, AngelikiMark, ManuelAskes, SvenMarketou, Maria E.Aslam, MuhammadMarklund, UlrikaAspatwar, AshokMarkopoulos, GeorgiosAspenström, PontusMarkova-Car, Elitza P.Assaf, Khaleel IbrahimMarkowicz-Piasecka, MagdalenaAssirelli, ElisaMarkus, KrebsAstolfi, AndreaMarla, SandeepAszodi, AttilaMarminon, ChristelleAtamian, HagopMármol, InesAtanasov, Petar AsenovMaroni, PaolaAtawia, ReemMarosi, ChristineAterini, StefanoMaroteaux, LucAthanasios, DalakourasMaroulis, GeorgeAtlas, EllaMarques, NatáliaAtrei, AndreaMarquez, EdgarAtsawasuwan, PhimonMarra, AlbertoAtsumi, GoMarranzano, MarinaAttanasio, ChiaraMarrazzo, PasqualeAttard, AgnèsMarrs, James A.Aubé, JeffreyMarsano, René MassimilianoAuberger, PatrickMarsh, JenniferAubert, JulieMarshall, Pamela A.Audi, SaidMarshall, PaulAuerbach, SanfordMarshall, Sarah A.Aung, Meiji SoeMarston, Steven B.Aurrand-Lions, MichelMartel, PauloAury-Landas, JulietteMartella, GiuseppinaAushev, VasilyMartelletti, PaoloAutry, JosephMartelli, Alberto MariaAvarvari, NarcisMartelli, FaustoAvdonin, Pavel V.Martelli, Pier LuigiAvignone, ElenaMarteyn, AntoineAviles, Francesc XavierMartha C., Rosales-HernándezAvin, VijayMartial, SoniaAvinoam, OriMartí-Calatayud, Manuel CésarAvnet, SofiaMartignago, DamianoAvonto, CristinaMartin Hernandez, Ana MontserratAvramidou, EvangeliaMartin, AidaAvramova, ViktoriyaMartin, AnkeAvrani, SaritMartin, FranckAvtanski, DimiterMartin, IanAwatade, NikhilMartín, Juan FranciscoAwe, SamuelMartin, PatriciaAyabe, TatsuhiroMartin, Sallyayalam, SrujanaMartin, SimonAyat, NadiaMartin, Tammy M.Aydin, YucelMartina, ByronAzarnia Tehran, DomenicoMartín-Acebes, Miguel A.Azer, Samy A.Martín-Antonio, BeatrizAzevedo, HerlanderMartín-Aragón, SagrarioAzevedo, JorgeMartinat, CécileAzuma, KotaroMartinelli, GiovanniBa, YongMartinez Alonso, MartaBaba, KenkichiMartinez Calejman, CamilaBaba, ShunsukeMartinez Garcia, Miguel AngelBaba, YasunoriMartínez Iglesias, OlaiaBabajko, SylvieMartínez, AlbertoBabenko, Nataliya A.Martínez, ConstantinoBabenko, Vladislav V.Martínez, GermánBabina, MagdaMartinez, ManuelBabu, AnishMartinez, VicenteBacakova, LucieMartínez-Campa, CarlosBacci, Maria LauraMartínez-Carmona, MarinaBach, Duc-HiepMartínez-Esteso, María JoséBąchor, RemigiuszMartínez-Flórez, SusanaBachschmid, MarkusMartínez-Gómez, PedroBack, KyoungwhanMartinez-Gonzalez, Luis J.Bácskay, IldikóMartinez-Guryn, KristinaBacso, ZsoltMartinez-Hervas, SergioBadawi, MohamedMartinez-Moreno, CarlosBadea, MihaelaMartinez-Pastor, FelipeBade-Doeding, ChristinaMartínez-Salas, EncarnaciónBadiger, BhaskaraMartinez-Sanchez, AidaBadino, PaolaMartínez-Sánchez, NoeliaBado, IgorMartinez-Useros, JavierBadowski, MichaelMartinez-Zubiaurre, ÍñigoBadr, ChristianMartinho, OlgaBadran, ZahiMartínková, LudmilaBadylak, StephenMartín-Montalvo, AlejandroBae, Eun HuiMartino, SabataBae, Hae-RahnMartinotti, SimonaBae, Ok-NamMartins Lima, AugustoBae, Woong JinMartins, AlbinoBaer, Patrick C.Martins, Andre F.Baerga-Ortiz, AbelMartins, IanBaert, YoniMartins, IsabelBagdziunas, GintautasMartins, José A.Bagetta, GiacintoMartins, Sandra Fátima FernandesBagherigaleh, SaadatMartins, SoniaBagnicka, EmiliaMartín-Valero, María JesúsBagyinszky, EvaMarto, JoanaBai, RenrenMartorana, AlessandroBai, YunMartos, SoledadBailey, Charles GMartz, FrancoiseBailly, ChristianMaruani, AntoineBaindara, PiyushMaruo, KohjiBaiocchi, MartaMaruyama, KeiBais, Manish V.Marvin (Kaplan), ShaunaBaj, GabrieleMarx, FlorentineBajaj, JeevishaMary, DidierBajaj, RuchikaMarycz, KrzysztofBajda, TomaszMarzaro, GiovanniBajinskis, AinarsMarzetti, EmanueleBak, AndrzejMarzo, TizianoBak, IstvanMarzocco, StefaniaBaker, Mark D.Mas, AymaraBaker, Sheila A.Mas, EricBakht, MartinMasahiro, SatoBakker, HansMasala, CarlaBakloushinskaya, IrinaMasalova, Olga V.Bakowsky, UdoMasana, LuisBal, WojciechMasarik, MichalBalabanova, LarissaMascitti, MarcoBalakirev, Evgeniy S.Maserti, Bianca ElenaBalalaeva, Irina V.Mashima, RyuichiBalan, SreeKumarMaslov, Mikhail A.Balarini, CamilleMasłyk, MaciejBalas, MihaelaMason, Howard J.Balasubramaniam, MeenakshisundaramMasonbrink, RickBalasubramanian, GaneshMass, ElviraBalasubramanian, KarthikMassa, ValentinaBalázs, MargitMassari, FrancescoBalboa, M. AngelesMassaro, MarikaBalcerczyk, AnetaMassella, DanieleBaldanzi, GianlucaMasson, DavidBaldassano, SaraMassotte, DominiqueBaldini, EnkeMastanjević, KristinaBaldo, Brian A.Masters, Colin L.Baldrich, PatriciaMastino, AntonioBalea, AnaMastinu, AndreaBalesdent, Marie-HeleneMastrangelo, Anna M.Balestrini, RaffaellaMastroianni, AntonioBalestrini, Raffaella MariaMastroleo, FeliceBall, JonathanMasuda, IsaoBall, VincentMasuda, JunkoBalla, JozsefMasuda, SachikoBallarini, FrancescaMasuda, SatohiroBallmer-Hofer, KurtMasui, KentaBallottari, MatteoMasullo, MariorosarioBalloy, VivianeMasutani, MitsukoBalme, SébastienMasuyer, GeoffreyBalogi, ZsoltMatarrese, PaolaBaloni, PriyankaMatchkov, VladimirBaluška, FrantišekMateo, CesarBalzano, TizianoMateo-Bonmatí, EduardoBambling, MatthewMateu, MauricioBamdad, CynthiaMatheï, CatharinaBamforth, SimonMatheu, M. IsabelBan, YusukeMathew, BobbyBan, ZoltanMathews, IrimpanBanach, ArturMathias, ClintonBanaszak, SzymonMathieu, DidierBandy, BrianMatias, Pedro M.Bandyopadhyay, DebasishMatichenkov, VladimirBanerjee, AditiMatijašic, MarioBanerjee, Hirendra NathMatikonda, SiddharthBanerjee, KalpitaMatilla, Angel J.Banerjee, Priya R.Matilla, MiguelBanerjee, SouravMatkowski, AdamBanerjee, UddyalokMato Prado, MireiaBanerji, VershaMatoba, KeiichiroBani, AlessiaMatos, ManuelaBanning, AntjeMatos, PauloBannon, SarahMatricardi, SaraBansal, AdityaMatrone, CarmelaBansal, Geetha P.Matsubara, KeiichiBanti, Christina N.Matsuda, AkioBao, AndeMatsuda, KoichiBao, YupingMatsuda, SatoshiBarabas, PeterMatsuda, ShinjiBarakat, Tahsin StefanMatsugaki, AiraBarakate, AbdellahMatsui, ToruBaran, AnnaMatsui, YasuhisaBaran, JaroslawMatsumoto, HideakiBarančík, MiroslavMatsumoto, HiroyukiBaranov, VladislavMatsumoto, Ken’ichiroBaranowska-Bociaska, IrenaMatsumoto, KunioBaranowska-Bosiacka, IrenaMatsumoto, TakayukiBarao, IsabelMatsumoto, TaroBarathi, Veluchamy AmuthaMatsumoto, YasuhikoBarbagallo, DavideMatsumoto, YoshinoriBarbanti-Bròdano, GiovanniMatsumura, TsuyoshiBarber, AmoretteMatsuo, KoichiBarbero, AndreaMatsuo, KojiBarbieri, FedericaMatsuo, MasafumiBarbillon, GrégoryMatsuo, MuneakiBarbolina, MariaMatsushima, AyamiBarbon, SilviaMatsuura, BunzoBarbone, AlessandroMatsuura, KazunoriBarbosa, Ana IsabelMatsuzaki, JuntaroBarbosa, Judite N.Matsuzaki, KeiichiBarca, AmilcareMatsuzaki, ToshiyukiBarceló-Coblijn, GwendolynMattei, CesarBarcelos, AnabelaMattei, VincenzoBarczak, WojciechMattoscio, DomenicoBardag-Gorce, FawziaMatucci-Cerinic, MarcoBardella, ChiaraMaturana, Andrés DanielBardi, GiuseppeMatusica, DusanBaregundi Subbarao, RaghavendraMatveev, VictorBarile, LucioMatwijczuk, ArkadiuszBarillari, GiovanniMaue, Robert A.Bařinka, CyrilMaugeri, GraziaBaritaki, StavroulaMaupin-Furlow, Julie A.Barłóg, PrzemysławMaurelli, Maria PaolaBarlow, James W.Maurizio, MartiniBarnes, JarrodMauro, Adolfo G.Barnet, Megan B.Mauro, AnnunziataBarnych, BogdanMaurya, ShailendraBaron, MarcoMavila, NirmalaBarra, FabioMavridis, IoannisBarra, GiusiMavridis, KonstantinosBarral, DuarteMavrogeni, SophieBarrell, Graham K.Mavrogonatou, EleniBarrero, Maria J.Maxwell, Jessie R.Barrios-Masias, Felipe H.Mayan, Maria D.Barros, Pedro M.Mayberry, JohnBarros-Timmons, AnaMaye, Peter F.Barrott, JaredMayerhofer, ArturBarrow, Alexander DavidMayer-Wagner, SusanneBarta, CsengeleMayne, ChristopherBartelt, AlexanderMayo, KevinBartley, JamesMayr, ChristianBartley, LauraMays, RyanBartolazzi, ArmandoMazerbourg, SabineBartolomeo, Antonio DiMazunin, IliaBarton, LudekMazur-Bialy, Agnieszka IrenaBartosik, MartinMazzaglia, AntoninoBartosz, GrzegorzMazzeo, Maria FiorellaBartoszewska, SylwiaMazzone, PaoloBartulos, OscarMazzoni, MaraBartuzi, DamianMazzucato, AndreaBarygin, Oleg I.Mazzulli, JoeBarzilay, JoshuaMba Blázquez, MiriamBasak, SujitMc Auley, Mark T.Basar, MuratMcAnany, J. JasonBaschant, UlrikeMcArdle, StephanieBasha, RiyazMcBride, Devin W.Bashir, KhurramMcCann, Matthew R.Bashtrykov, PavelMcCary, Lindsay M.Basiak, EwelinaMcCaughan, Geoffrey WilliamBasilico, CristinaMcClain, Kenneth L.Basini, GiuseppinaMcCormick, RachelBasiricò, LoredanaMcDermott, Suzanne M.Basit, AbdulMcDonagh, BrianBasith, ShaherinMcDonald, PaulBaskin, Kedryn.McDonald, ThomasBaslam, MarouaneMcGee-Lawrence, MeghanBasnet, SanjayMcJarrow, PaulBasoli, ValentinaMcKay, BrianBasrai, MuniraMcKenna, Malachi J.Bassareo, Pier PaoloMcLemore, Gabrielle LynnBasso, DanielaMcMahon, StephenBasso, KariMcManus, KirkBaston-Büst, Dunja MariaMcMillin, MatthewBastos, JulioMcmorrow, TaraBasu, DebaratiMcNagny, Kelly M.Basu, DipanjanMcNutt, PatrickBaswan, SudhirMcPherson, BradleyBata-Csorgo, ZsuzsannaMcWhorter, AndreaBatchu, RameshMeade, AidanBatinic-Haberle, InesMeana Paneda, RubenBatistič, OliverMears, JasonBatley, JacquelineMeccariello, RosariaBattafarano, GiuliaMechaly, ArielBattaglia, EricMecocci, PatriziaBattaglino, RicardoMedici, ValentinaBattista, NataliaMedina Piles, VicenteBattista, SabrinaMedina, Francisco JavierBattistelli, CeciliaMedina, Gisselle N.Battistelli, MichelaMedina, Reinhold J.Battula, NarendraMedová, MichaelaBaubec, TuncayMedraño-Fernandez, IriaBaucher, MarieMeduri, AlessandroBauckneht, MatteoMedvedev, AlexeiBaucum, Anthony J.Meerang, MayuraBaudin, BrunoMeerson, AriBaudis, StefanMegighian, AramBaudoin-Dehoux, CécileMeglič, VladimirBauer, BjörnMegyeri, K.Bauer, GeorgMehla, JogenderBauer, JohannMehrotra, ShikharBauersachs, StefanMehta, SunaliBaulin, Vladimir AMei, ShenglinBaulina, NataliaMei, YangBaumann, HeinzMeier, Kathryn E.Baumann, MarcusMeijer, Harold J GBaumgart, Simon J.Meijer, OnnoBaumgarten, ThomasMeinke, PeterBautista, MartaMeiri, HamutalBawadekar, MandarMeisel, ChristianBay, Denice C.Meisel, Hans-JörgBay, PeterMeissner, AnjaBayat Mokhtari, RezaMejuto, JuanBayerl, ChristianeMekori, Yoseph A.Bayin, N. SumruMelanitou, EvieBaykov, SergeyMelcher, KarstenBayliss, Christopher D.Meldolesi, JacopoBazán, EulaliaMele, LauraBazhanova, Elena DMeleady, PaulaBazhin, Alexandr V.Melin, FredericBazinet, LaurentMellies, JayBazrafshan, ZahraMelnikov, StanislavBazwinsky-Wutschke, IvonneMelo, Ana M.P.Bazylińska, UrszulaMelo, LuísBeard, MatthewMelo, Sonia A.Beatson, Richard E.Melone, Mariarosa Anna BeatriceBeberok, ArturMelrose, JamesBecerra, ManuelMemon, TosifaBecerril, SaraMenale, CiroBechmann, NicoleMenasché, PhilippeBeck, William T.Mendes, Larissa LouresBeckel, J. M.Mendes, Livia P.Becker, JürgenMendes, MateusBecker, Katrin AnneMéndez, Loyda B.Becker, ThereseMéndez, LucíaBecker, ThomasMenegazzi, MartaBecker, YvonneMeneguzzo, FrancescoBecker-Pauly, ChristophMenendez, EstherBeckervordersandforth, RuthMenezes, Miriam-RoseBecskei, AttilaMeng, DongBednar, PetrMeng, GeBednarska-Szczepaniak, KatarzynaMeng, HaoBednarski, Patrick J.Meng, JinhongBedoyan, Jirair KMennerich, DanielaBeebe, StephenMenon, ManojBeeckman, TomMenon, VipinBegcy, KevinMensah, Enoch A.Beharry, Kay D.Menu, ElineBehringer, ErikMerah, OthmaneBehzadi, PayamMeraviglia, VivianaBeilby, MaryMerdes, AndreasBeineke, AndreasMereiter, StefanBeirowski, BogdanMergeay, MaxBeis, DimitrisMergia, AyalewBekeschus, SanderMergler, StefanBeld, JorisMeriney, Stephen D.Belfrage, Anna KarinMerino, Gabrielbell, StephenMerino, Jaime M.Bellardita, CarmeloMerino, Jose JoaquinBellavia, DanieleMerli, ManuelaBellelli, RobertoMerret, RémyBellini, Maria IreneMertas, AnnaBello, LucaMerviel, Philippe.Bellucci, AriannaMesaros, Clementina A.Belluzzi, ElisaMeschwitz, SusanBelosludtsev, KonstantinMeseda, ClementBel’skaya, LyudmilaMesirca, PietroBeltowski, JerzyMesquita, JoãoBeltran, ChrisMesquita-Guimarães, JoanaBel-Vialar, SophieMessner, BarbaraBenada, JanMessori, LuigiBenarba, BachirMessyasz, BeataBenavides-Mendoza, AdalbertoMester, Patrick-JulianBencivenga, StefanoMesuraca, MariaBencsik, PeterMetes, Diana M.Bendahhou, SaïdMetz, Stefan W.Ben-David, YaacovMetzger, JuliaBender, KyleMetzinger, LaurentBenedetti, MicheleMeulenberg, ElineBenes, PetrMeurer, Steffen KlausBeng Kah, SongMeuris, BartBengalli, RossellaMeyer, FlorentBeninati, SimoneMeyerholz, David K.Benini, StefanoMezzadra, RiccardoBenistant, ChristineMezzetti, MatteoBenito, BegoñaMian, RoufBenito, Ernesto P.Miao, TianxinBenjamin, WylieMicarelli, AlessandroBenn, PeterMichalak, KrystynaBennett, James P.Michalek, KatarzynaBenoit, MatthiasMichallet, Marie-CécileBenrick, AnnaMichalopoulos, GeorgeBentel, Jacqueline M.Michard, ErwanBentrop, JoachimMicheau, OlivierBenussi, StefanoMichel, MaximilianBen-Yosef, TamarMichel, PiotrBequignon, EmilieMichel, SebastianBeranova-Giorgianni, SarkaMichelhaugh, SharonBerardi, EmanueleMichelle, PalmieriBerardo, ClarissaMichelsen, Trond MelbyeBerenjian, AydinMichetti, FabrizioBerent-Maoz, BeataMichiels, CarineBeretta, Giovanni L.Michinaga, ShotaroBerg, Lise C.Michl, Thomas D.Bergamo, AlbertaMichlewski, GracjanBergandi, LoredanaMicillo, RaffaellaBergdahl, AndreasMicol, José LuisBerger, FredericMicotti, EdoardoBerger, Ralf G.Micó-Vicent, BàrbaraBerger, WalterMiddei, SilviaBergholz, TeresaMiddelkoop, EstherBerglund, J. AndrewMiękus, NataliaBergonzi, SaraMielańczyk, AnnaBergonzo, ChristinaMiele, Adriana EricaBergougnoux, VéroniqueMielenz, DirkBerlanga, Ángel MéridaMieliauskaitė, DianaBerlin, K. DarrellMierek-Adamska, AgnieszkaBermudez, MarcelMiernyk, Ján A.Bernacchioni, CaterinaMiethke, ThomasBernal, VicenteMigaud, Marie E.Bernard, KristenMigliorini, DenisBernard, Marek K.Migonney, VéroniqueBernardes, NunoMíguez, Jesús M.Bernardi, HenriMihály, AndrásBernardi, SaraMi-ichi, FumikaBernardini, GiuliaMijailovich, SrbaBernardino, Raquel L.Mikac, Lara M.Bernards, MatthewMikhailova, ElenaBerndt, Michael C.Miki, YasuhiroBernhardt, Harold S.Mikiewicz, MateuszBernstein, Audrey M.Mikolajczyk, AnitaBerry, James OMikropoulos, ChristosBerta, TemuginMikstacka, RenataBerteau, Jean-PhilippeMikula, MarioBertelloni, FabrizioMilano, FrancescoBerthelot, LaurelineMilano, SerenaBerthier, CelineMilanovic, DesankaBertinaria, MassimoMilatz, SusanneBertini, LauraMilcarek, ChristineBertocchini, FedericaMilczarski, PawelBertran, AndreMildner, MichaelBervoets, LieneMilella, LuigiBesalú, EmiliMiles, Wayne OwenBesendorfer, VišnjaMilioto, StefanaBessa, LucindaMilisav, IrinaBessmertnykh-Lemeune, AllaMilković, LidijaBesteiro, SébastienMill, Jose GeraldoBetekhtin, AlexanderMillan, TeresaBethanis, KostasMillar, AnthonyBetsou, FayMillard, AndrewBettache, NadirMILLEMANN, YvesBetti, CamillaMiller, CharlesBevilacqua, ArturoMiller, DennisBevington, AlanMiller, GalenBeyer, Andreas M.Miller, IngridBeyer, Eric C.Miller, JustinBeyer, GeorgMiller, Robert H.Beyer, SebastianMiller, Walter L.Beyerlein, KennethMillet, ÓscarBezdetnaya-Bolotine, LinaMills, KenBhagirath, DivyaMilne, RickyBharadwaj, ShishiraMilosavljević, AleksandarBharadwaj, VivekMilosavljevic, NinaBharath, Leena P.Milošević, Ivana S.Bhargava, PavanMilovanovic, DragomirBhaskara, Govinal BadigerMimura, JunseiBhat, KamakotiMin, DuBhat, KruttikaMin, Kyung-JinBhatia, Harsharan S.Minaga, KosukeBhatta, MadhavMinami, MasaakiBhatta, UshaMinami, YoichiBhattacharjee, SonaliMinami, YosukeBhattacharya, DebswapnaMinamisawa, SusumuBhattacharya, SupriyoMinarovits, JanosBhattacharyya, SwatiMinde, DavidBhattarai, Kashi RajMine, YuichiBhattarai, KrishnaMineharu, YoheiBhatti, DanishMinematsu, ToshioBhatti, M.M.Minetti, GiampaoloBhavsar, Amit P.Mingione, AlessandraBhetwal, Bhupal P.Mingo, AntonioBhosekar, VijayMinguet, Eugenio G.Bhowmik, Pradip K.Miniero, Daniela ValeriaBhusal, Siddhi JeewanMink, MátyásBiagi, MarcoMino, MasanobuBiagini, GiuseppeMinocha, Subhash C.Białek, MałgorzataMintz, C. DavidBianchi, ElisaMinucci, AngeloBianchi, VittorioMinucci, SergioBianchini, FrancescaMiotla, PawelBianco, AndreaMiotto, PaulaBianco, CarmelinaMiquel, MartineBianco, PieroMir, RicardoBiase, FernandoMiralles, FranciscoBibb, James A.Miranda, CristobalBichet, Daniel G.Miranda, João MárioBidère, NicolasMiranda, MaríaBidwai, AshokMiranda, Rafael De SouzaBielska, EwaMirete, SalvadorBierle, CraigMironova, Victoria V.Biernasiuk, AnnaMirzayans, RazmikBiersack, BernhardMishin, Alexander S.Biffis, AndreaMishra, AwdheshBihari, NevenkaMishra, DushyantBijak, MichalMishra, Jay S.Bijlsma, Maarten F.Mishra, ManishBikbova, GuzelMishra, Pawan KumarBikiaris, DimitriosMishra, RajeshBikov, AndrasMishra, SasmitaBilbao, DanielMiskulin, MajaBilewicz, AleksanderMisra, Biswapriya B.Bilic-Ćurčić, InesMistiaen, WilhelmBilski, JanMistry, PramodBindila, LauraMitash, NilayBirchler, James A.Mitchell, Cassie S.Bird, David Mck.Mitchell, DavidBird, RanjanaMitchell, ToddBirket, SusanMitkin, Nikita A.Biro, OrsolyaMitoma, HirokiBironaité, DaivaMitrofanis, JohnBiscetti, FedericoMitrofanova, AllaBishop, KarenMitsui, ToshiakiBisio, AlessandraMitsuya, ShiroBisti, SilviaMittapelly, PriyankaBiswas, SangitaMittelsten Scheid, OrtrunBiswas, SantanuMittmann, ThomasBitonti, MariaMiura, NatsukoBitra, ArunaMiura, YokoBizzarri, MarianoMiyagaki, TomomitsuBjelland, SveinMiyagawa, KiyoshiBlack, William CMiyake, TerukiBlais, AnneMiyamoto, HirotakaBlakely, William F.Miyamoto, KojiBlanco, AntonioMiyanaga, AkimasaBlanco, José Manuel LópezMiyanishi, TakayukiBlanton, CynthiaMiyata, JunBlasiak, JanuszMiyata, NaoyukiBlaya, DeliaMiyata, YasuyoshiBlazevic, IvicaMiyoshi, HiroakiBlazquez, Ana-BelenMiyoshi, TakekazuBlein, ThomasMizejewski, Gerald JBlein-Nicolas, MélisandeMizielinska, SarahBlikslager, AnthonyMizuno, MasashiBlind, RaymondMizuno, NobuyukiBloch, WilhelmMizuno, TooruBlock, StephanMizunoya, WataruBloise, EnrricoMizuo, KeisukeBloise, NoraMladěnka, PřemyslBlomme, EricMlejnek, PetrBlondel, DanielleMlocicki, DanielBlonska, MarzennaMo, ChenglinBluett, JamesMo, FeiBlum, ArnonMo, YanyuanBlumer-Schuette, Sara E.Mobasheri, AliBoard, MaryMoccia, FrancescoBoateng, JoshuaMoccia, MarcelloBoaventura, PaulaMochida, KeiichiBobbala, SharanModenutti, CarlosBobinger, MarcoModepalli, VengamanaiduBoccella, SerenaModhiran, NaphakBocchinfuso, GianfrancoModica, MariaBocharov, Eduard V.Modjtahedi, HelmoutBocianowski, JanModos, DezsöBock, Karl WalterModrowski, DominiqueBöckmann, Rainer AMoen, ErikaBoda, Sunil KumarMoertl, SimoneBoddapati, LoukyaMoffa, Matthew A.Boddu, Sai H. S.Moghaddam, ArashBodea, LiviuMogi, MasakiBodenstine, ThomasMogilski, SzczepanBoeckel, Jes-NielsMoha Ou Maati, HamidBoege, FritzMohamed, Azza H.Boehm, StefanMohamed, F. AttiaBoekema, BoukeMohamed, Salah A.Boerma, MarjanMohamed, SharmarkeBoevink, PetraMohamed, TamerBogacka, IwonaMohamed, WaelBogdanos, Dimitrios P.Mohamedali, KhalidBogdanova, AlionaMohammad, ArifBogdanowicz, Krzysztof ArturMohammad, Haroon TajBoggaram, VijayMohammad, Ramzi M.Bogusławska, JoannaMohammadiarani, HosseinBøgwald, JarlMohanram, HariniBoichuk, SergeiMohanta, Tapan KumarBoink, Gerard J. J.Mohanty, VakulBoisivon, Helene RobertMohd Hanif, ZulfakarBoix, EsterMohd, Farooq ShaikhBojarová, PavlaMoin, MazaharBojková, BiankaMojib, NaziaBoldogh, IstvanMold, Matthew J.Boling, Warren W.Molenaar, Remco J.Bolotov, LeonidMolesini, BarbaraBölükbas, DenizMolina, AnaBoman, JesperMolina, Cristina E.Bombard, SophieMolina-Cerrillo, JavierBombardieri, StefanoMolinari, SusannaBombieri, CristinaMoliner, Candela CuestaBonanno, GiambattistaMolinier, JeanBonaventura, AldoMolins, BlancaBoncela, JoannaMoll, Ute M.Boncoeur, EmilieMoller Wolmarans, MarisaBongarzone, ItaliaMøller, IanBongers, KaleMøller, Ian MaxBonifacio, Maria AddolorataMøller, PålBono, Jean JacquesMollica Nardo, VivianaBono, RobertoMollica, AdrianoBonsi, LauraMołoń, MateuszBoonen, MarielleMoltzau, Lise RománBooth, BrianMolvarec, AttilaBooth, Daniel G.Momen, MehdiBooth, DavidMonaghan, DanielBooth, Kevin T.Monahan, Thomas S.Bopegamage, ShubhadaMonari, AntonioBorbély, JánosMoncharmont, BrunoBorbély, YvesMonclus, MichelBordin, LucianaMonferran, SylvieBordonaro, MichaelMong, Mei-ChinBordons, AlbertMongin, AlexanderBorek, SławomirMoni, LisaBorger, VerenaMonji, AkiraBorghaei, Ruth C.Monk, PeterBorghi, MonicaMonsalve, MariaBorghini, AndreaMonsarrat, PaulBorkow, GadiMontag, DirkBorodkina, AleksandraMontagnani Marelli, MarinaBoros, LaszloMontagne, KevinBoros, LászlóMontagner, DiegoBorota, AnaMontague, SamanthaBorovac, Josip A.Montalvo-Rodríguez, RafaelBorovikov, Yurii S.Montanaro, LorenzoBorradale, DavidMontecucco, FabrizioBorrego, Ana AriasMonteiro, FilipaBorriello, AdrianaMonteleone, IvanBorriello, LuciaMontenegro, LuciaBorroni, Elena MonicaMontes Resano, MartaBorsani, ElisaMontgomery, McKaleBorse, VikrantMonti, BarbaraBortolozzi, MarioMoody, WilliamBortolus, MarcoMooers, BlaineBorup Jensen, SvendMookerjee, ShonaBorymski, SławomirMookherjee, NeelofferBoscaiu, MonicaMoolhuijzen, Paula M.Bosch, ChristineMoon, Hong ChulBOSCHI-MULLER, SandrineMoon, Jong-SeokBoscia, FrancescaMoon, KyungBose, SaurabhMoon, MinhoBose, TanimaMoon, Yong-HwanBosi, SaraMoore, Roger A.Bosman, GielMoore, StuartBosnjak, BerislavMoracci, MarcoBosserhoff, AnjaMorahan, GrantBostan, HamedMorales, JulioBotelho, GorettiMorales, PaulaBotelho, MonicaMorales-Garcia, Jose A.Botta, BrunoMorales-González, José AntonioBoublikova, LudmilaMorales-Narváez, EdenBouché, MarinaMorales-Quintana, LuisBoucheix, ClaudeMoralli, DanielaBoudhraa, ZiedMoran, OscarBoulaiz, HouriaMorán, PalomaBoulling, ArnaudMorbidelli, LuciaBouřa, EvženMoreau, MaximBourdon, EmmanuelMoreira, Felismina T.C.Bourgeois, DominiqueMoreira, PaulaBourgoin, SylvainMorel, BertrandBourneuf, EmmanuelleMorello, SilvanaBousbaa, HassanMoreno, AdrianBoušová, IvaMoreno, AndrésBousquet, GuilhemMoreno, Ricardo D.Boussios, StergiosMoreno, SandraBoutin, JeanMoreno, SergioBouyahya, AbdelhakimMoreno-Indias, IsabelBouyer, DanielMoreno-Piraján, Juan CarlosBovolin, PatriziaMoreno-Villanueva, MariaBowen, AnthonyMoret, FrancescaBowling, Heather L.Moretó, MiquelBowsher, AlanMoretti, RitaBoyanova, LyudmilaMorgan, Iain M.Boyd, LesleyMorgan, MichaelBoyer, Justin G.Morganti, PierfrancescoBoysen, ReinhardMorgera, Alessandro FraleoniBozzi, FabioMorgese, Maria GraziaBraakhuis, AndreaMorgillo, FlorianaBrábek, JanMori, HirotadaBracci, MassimoMori, KiyoshiBracco, EnricoMori, MasakiBracht, ThiloMori, MattiaBrack, AndreMori, ShigekiBraczkowski, RyszardMoribayashi, KengoBradáčová, KláraMoricoli, DiegoBradbury, AllisonMoriconi, FedericoBradfute, StevenMorigi, RitaBradshaw, NicholasMoriguchi, TakayaBradshaw, PatrickMorimura, ShigeruBragado, M. JuliaMorin, MatiasBraglia, LucaMorino, KatsutaroBraicu, CorneliaMorioka, TomoakiBraig, SimoneMorison, IanBraliou, GeorgiaMorisset-Lopez, SéverineBramanti, VincenzoMorita, MitsuhiroBramham, CliveMorita, ShigetoBrancati, RenatoMorita, ToshisukeBrand, KorbinianMorito, EndoBrand, ThomasMoriya, ManabuBrandenburg, KlausMoriya, TakahiroBrandli, AliceMoriyama, YoshinoriBranstetter, Michael G.Morkunas, IwonaBranza-Nichita, NoricaMorland, CecilieBratashov, Daniil N.Moroncini, GianLucaBraun, Andrew P.Morozova, OlgaBraun, Frank K.Morris, BrianBraun, SimonMorris, Brian J.Braun, WernerMorris, James L.Braunstein, Evan M.Morris, John B.Bravo-Diaz, CarlosMorris, MhairiBrcic, LukaMorrison, MelanieBréchard, SabrinaMortara, LorenzoBrecker, LotharMosbah, Ismail BenBreckpot, KarineMosca, LucianaBrehmer, AxelMoschovi, MariaBreitbart, HaimMosharov, Eugene V.Brem, JurgenMoskalev, AlexeyBrender, JeffreyMoskot, MartaBrenišin, MarekMosqueira, MatiasBrennan, CharlesMoss, WalterBrenner, Annette K.Mostafavi, MehranBrenner, RolfMostofa, Agm G.M.Brents, Lisa K.Mostowska, AgnieszkaBresson-Bepoldin, LaurenceMot, AugustinBrestic, MarianMota, ManuelBrew-Appiah, RhodaMotlagh Scholle, LeilaBrezovský, JanMotloch, Lukas JBrieger, AngelaMotohashi, NorioBrigelius-Flohe, ReginaMotrich, Rubén DaríoBrigido, ClarisseMotta, Chiara MariaBrill, AlexanderMou, HaiweiBrillante, LucaMou, YongchaoBrindley, MelindaMoulder, RobertBrinkmann, VolkerMouncey, Nigel J.Brites, PedroMounien, LourdesBrito, PauloMousa, Shaaban A.Briza, PeterMousley, CarlBrizuela, LeyreMoustaïd-Moussa, NaïmaBroadbent, AndrewMouzat, KévinBroderick, PatriciaMouzeyar, SaïdBroderick, Tom L.Movahedi, AliBrodlie, MalcolmMovassagh, HesamBrodosi, LuciaMoyes, AmieBroer, StefanMoyle, LouiseBroering, RuthMozos, IoanaBrogden, GrahamMrakovcic, MariaBroggi, GiuseppeMrówczyńska, LucynaBroggini, MassimoMrówczyński, RadosławBrogi, SimoneMróz, WaldemarBromme, DieterMuchembled, JeromeBroniatowski, MarcinMucsi, ZoltánBronowska, AgnieszkaMueller, Wolf C.Brooks, Craig R.Muhammad, SahimiBroom, IainMühl, HeikoBros, MatthiasMühle, ChristianeBrosnahan, C. L.Mühlfeld, ChristianBross, PeterMukai, ShoichiroBrouwer, StephanMukai, TomoyukiBrouwers, Jos F.Mukaida, NaofumiBrown, AlexMukherjee, RajibBrown, Craig E.Mukherjee, SarbajitBrown, Euan RMukherjee, SudipBrown, JamesMukherjee, SumitBrown, KatherineMukhopadhyay, SubhradipBrown, LindsayMukhopadhyay, SumanBrown, Ronald B.Müller, ChristianBrownmiller, CindiMüller, Gerhard AntonBrozovic, AnamariaMüller, LudmilaBruegge, Jennifer ZurMuller, Patricia A. J.Bruegmann, TobiasMüller, PatrickBrüggemann, MonikaMüller, SabineBrulíková, LucieMüller, ThomasBrundage, Cord M.Mullin, James M.Brune, WolframMulthoff, GabrieleBrunet, FrédéricMuniyan, SakthivelBrunetti, GiacominaMunkley, JenniferBrunetti, LuigiMuñoz Caffarel, MaríaBrunner, SusanneMuñoz, Estela M.Brunner-Weinzierl, Monika ChristineMuñoz, Francisco JoséBruno, GiovanniMuñoz, Marcelo AndrésBruserud, ØysteinMuñoz-Chápuli, RamónBrussino, LuisaMuñoz-Torrero, DiegoBrusslan, JudyMunro, AndrewBrutch, NinaMuntané, GerardBruzzaniti, AngelaMuntimadugu, EameemaBruzzone, SantinaMunusamy, ShankarBryant, Andrew J.Mupo, AnnalisaBryant, Christian E.Mura, CatherineBrylinski, MichalMuraca, MaurizioBrys, JoannaMurai, KojiBrzezińska-Błaszczyk, EwaMurai, NoriyukiBrzeziński, KrzysztofMurakami, YasufumiBrzóska, EdytaMuraki, KatsuhikoBrzozowski, TomaszMuranov, KonstantinBubici, GiovanniMurase, SachikoBucci, CeciliaMurata, JunBüchel, ClaudiaMurata, TakayukiBuchet, ReneMurayama, HidekiBuchheim, JudithMurdaca, GiuseppeBüchler, ChristaMurgia, MartaBuchoux, SebastienMuriach, MaríaBücker, RolandMuriel, PabloBucki, RobertMurolo, SergioBuck-Koehntop, Bethany A.Murotomi, KazutoshiBucur, DanielMuroya, SusumuBudak, HikmetMuroya, YoshikazuBudinsky, Robert A.Murphy, AndrewBudisa, NediljkoMurphy, CormacBudnyak, TetyanaMurphy, EainBudryn, GrażynaMurphy, RonanBudzisz, ElżbietaMurray Stewart, TracyBueno, ClarissaMurray, JeremyBuettner, ChristophMurray, LyndsayBujacz, GrzegorzMurray, SandraBukowska, JoannaMuruganandan, ShanmugamBukowski, MichalMusalgaonkar, SharmishthaBulatov, EmilMusani, VesnaBulbarelli, AlessandraMusarò, AntonioBulc, MichałMusazzi, LauraBüll, ChristianMusch, MarkBulla Jr, LeeMuschiol, JanBulli, PeterMuschter, DominiqueBullock, MartynMuscogiuri, GiovannaBumbacea, DragosMusgrave, IanBun NG, TziMusso, NataleBunaciu, Rodica PMustafaf, GhazalaBunik, VictoriaMusti, Anna MariaBuonaguro, Elisabetta FilomenaMusumeci, GiuseppeBuoncervello, MariaMuszyński, SiemowitBuratta, SandraMuthamilarasan, MehanathanBuravkova, LudmilaMuthu Karuppan, Mohan KumarBurchakov, Denis I.Muthukumar, ThangaveluBurdick Sanchez, NicoleMuthuramu, IlayarajaBurek, MalgorzataMutoh, TatsuroBurgalassi, SusiMutti, LucianoBurger, Michael CMuzio, GiulianaBurger, RenateMyasoedova, VeronikaBurgess, Sean M.Myhrstad, Mari C.W.Burgstaller, JoergMyllymaa, SamiBurlacu, AlexandrinaMyrianthopoulos, VassiliosBurlingham, WilliamMyśliwiec, HannaBurnette, Pearlie K.Mystkowska, JoannaBurns, Jorge S.Myung, HeejoonBurrello, JacopoNa, DokyunBurt, Andrew J.Nachtigal, PetrBurtey, StéphaneNaclerio, GinoBurton, Zachary FNader, GustavoBuryanov, YaroslavNadiminty, NagalakshmiBusardò, Francesco PaoloNadler, Jerry L.Busatto, NicolaNadykto, Alexey B.Busch, AlbertNaegele, RachelBusch, MaikeNaftalin, RichardBusinaro, RitaNag, AnitaBussolati, OvidioNagai, AikoBustamante, AlejandroNagai, KouheiBustamante, ClaudiaNagaraj, ViniBustos, Victor H.Nagarajan, BalajiBuszewicz, DanielNagarajan, RagupathiButelli, EugenioNagaraju, Ganji PurnachandraButler, Georgina SNagase, KenichiBuxton, DenisNagashima, YukihiroBuxton, SamuelNagasundaram, NagarajanByler, Kendall G.Nagatoishi, SatoruByrdin, MartinNagel, RaimundByrne, AnthonyNaghibalhossaini, FakhraddinByrne, ScottNagpal, RavinderByun, JonghoeNagy, BálintByun, KyungheeNagy, GáborByun, SanguineNagy, IstvanBywater, Robert P.Nagy, Laszlo G.C Beitz, DonaldNagy, NadineCaaveiro, JoseNagy, NandorCaballero-Garcia, BeatrizNagy, PeterCabantous, StéphanieNagy, TamásCabaro, SerenaNahar, LamiaCabeza Gras, ÓscarNahomi, Rooban B.Cabot, MylesNair, Gopika G.Cabrele, ChiaraNair, MeeraCabrera-Fuentes, Hector AlejandroNair, Praful R.Caburet, SandrineNair, ShinyCaccamo, DanielaNairz, ManfredCacciotti, IlariaNaito, KatsuyukiCadamuro, MassimilianoNaito, ToshioCadet, JeanNaitoh, MotokoCadigan, KennethNajar, AmerCaeiro, MariaNajda, AgnieszkaCaffrey, PatrickNaka, HiroakiCafiero, MauricioNakabayashi, KazumiCagnin, StefanoNakagawa, KimieCahill, MichaelNakagawa, YoshimiCahoon, A. BruceNakahama, Ken-ichiCahova, MonikaNakajima, MasahiroCai, DeminNakamaru-Ogiso, EikoCai, GiampieroNakaminami, KentaroCai, JiyangNakamoto, MasatoshiCai, XiaNakamura, AkinobuCaiazzo, ElisabettaNakamura, KyoheiCailliez, FabienNakamura, MasanaoCaironi, PietroNakamura, MasatoCairrão, ElisaNakamura, MotokiCaivano, AntonellaNakamura, NobuhiroCakouros, DimitriosNakamura, TakehiroCakstina, IneseNakamura, TomokiCal, SantiagoNakamura, TsukasaCalabrese, Edward J.Nakamura, YoshiyukiCalabrese, GiovannaNakanishi, AkiraCalabrese, VittorioNakanishi, TakeoCalado, Cecília R.C.Nakano, KeisukeCalcinotto, AriannaNakano, MichiharuCalcutt, MichaelNakano, Shu-ichiCaldara, MarinaNakano, TakanariCaldo, Kristian MarkNakao, TetsushiCaldon, C. ElizabethNakase, HiroshiCaldwell, R. WilliamNakashima, KazuoCaliandro, RoccoNakashima, SouichiCaligiuri, IsabellaNakashima, YoshikiCalixto, Cristiane P.Nakata, MiyukiCalla, BernardaNakatani, YoshihikoCallery, Patrick S.Nakatsuji, TeruakiCalvano, Cosima DamianaNakaya, NaokiCalvert, Amanda E.Nakayama, HironaoCalvisi, Diego FrancescoNakayama, KohCalvo Polanco, Maria MonicaNakayama, MizuhoCalvo, Ana CristinaNakayama, YasumuneCalvo-Polanco, MonicaNakazawa, MitsuruCamara, Amadou K.S.Nalivaiko, EugeneCamberg, Jodi L.Nallasamy, PalanisamyCamejo, GermánNam, Jin-WuCameron, DonaldNam, KwanghoCametti, CesareNamasivayam, VigneshwaranCamilli, EmanuelaNanasato, YoshihikoCaminati, GabriellaNanba, EijiCamins, AntoniNanda, IndrajitCammalleri, MaurizioNandi, SaikatCampa, ClaudineNanjappa, DeepakCampanella, AlessandroNanjundan, MeeraCampanella, ClaudiaNapolitano, AlessandraCampanini, BarbaraNapolitano, FrancescoCampbell, Corey L.Napolitano, MariasantaCampbell, EwanNaranjo-Hernández, DavidCampesi, IlariaNarayanaswami, VasanthyCampione, MarinaNardelli, CarmelaCampoccia, DavideNardelli, SilviaCampos, CatarinaNardi, MonicaCampos, EricNardi, SerenellaCampos, NarcisoNarendra, DerekCamps, ManelNaro, FabioCampuzano, AltheaNarra, Hema PrasadCampuzano, OscarNaruishi, KojiCanali, RaffaellaNaryzhny, StanislavCanals, MeritxellNarzisi, AntonioCañas, Rafael A.Nascimento, Gustavo G.Cancela, LeonorNashima, KenjiCancemi, PatriziaNashine, SonaliCanfield, ScottNasrallah, June B.Cangkrama, MichaelNass, NorbertCanini, AntonellaNasser, MCannarella, RossellaNassir, FatihaCannavo, AlessandroNatarajan, Sathish KumarCannone, ValentinaNath, Ujjal KumarCansby, EmmelieNath, Vishnu SukumariCantalapiedra, Carlos P.Natile, GiovanniCantini, FrancescaNativ, DudaiCantley, Melissa D.Natsumeda, ManabuCañueto, JavierNault, RanceCao, ChuanhaiNaumann, Renate L. C.Cao, Hai-QunNaumov, AntonCao, Hieu X.Navakauskienė, RūtaCao, RenzhiNavarro, AlfonsCao, WeiguoNavazio, LorellaCao, ZheNaveira, HoracioCapanoglu, EsraNaviglio, DanieleCaparros-Martin, JoséNawaz, MuhammadCaparrotta, StefaniaNawaz, Muhammad AmjadCapasso, RaffaeleNawrot, BarbaraCapel, BlancheNaya, FranciscoCapel, CarmenNayak, TapanCapilla-Capilla, VivianNaz, AliCaplan, AllanNazar, Ross N.Cappariello, AlfredoNazarov, AlexeyCappelletti, GraziellaNazimek, KatarzynaCappellini, FrancescaNazzaro, GianlucaCappuccilli, MariaNduom, EdjahCaprini, MarcoNeagu, MonicaCapurro, ClaudiaNebbioso, MarcellaCarafa, MariaNebeská, DianaCaramalho, IrisNeerukonda, SabarinathCarapelli, AntonioNefzger, ChristianCarapito, RaphaelNeginskaya, Maria A.Carata, ElisabettaNegishi, TakayukiCarazo, AlejandroNegrea, PetruCarballo, IriaNehmé, ReineCarbonell, AlbertoNeira, José L.Carbonell, TeresaNelson, AndrewCarboni, LuciaNelson, ChristianCardillo, CarmineNelson, ErikCardoso, HéliaNelson, Fred R.T.Cardoso, SusanaNeman, JoshCardoso, Taina FigueiredoNemeș, Roxana MariaCarella, PhilipNémeth, BalázsCaretta, AntonioNemoto, WataruCaretti, AnnaNeppl, Ronald LCarey, AmandaNeri, IzaakCargnoni, FaustoNeri, SimonaCaria, PaolaNesbitt, Warwick S.Carillo, PetroniaNesmelov, Yuri E.Carlberg, CarstenNestor, CaseyCarli, Marco DeNestrasil, IgorCarlos, Ana RitaNeubauer, KatarzynaCarlotto, SilviaNeufeld, GeraCarmela, NardelliNeugebauer, DorotaCarmichael, Gordon G.Neumann, DetlefCarneiro, Sueli Coelho Da SilvaNeumann, KonstantinCarnevali, OlianaNeureiter, DanielCaro, ElenaNeves, JoãoCarobbio, StefaniaNewman, LauraCarol, PierreNewton, PhillipCaroleo, Maria CristinaNexø, EbbaCarolina, Alonso GonzálezNeyra, Javier A.Caron, KathleenNeyroud, DariaCarotenuto, MarcoNg, Hooi HooiCarotti, SimoneNg, Qin XiangCarpene, ChristianNg, Roy Chun-LaamCarpenter, GHNg, Tzi BunCarpenter, RichardNg, Wai-LungCarradori, SimoneNgamcherdtrakul, WorapolCarrasco, PedroNgezahayo, AnacletCarrasco-Pozo, CatalinaNguyen Phuoc, LongCarravetta, VincenzoNguyen, Binh P.Carrera, IvanNguyen, Linh T.BCarreras, JoaquimNguyen, Minhhuyen T.Carrieri, AntonioNguyen, Ngoc A.Carrillo-Tripp, MauricioNguyen, Quang D.Carro, LauraNguyen, Thanh BinhCarroll, Lara S.Nguyen, Thanh HCarrouel, FlorenceNguyen, Van KhanhCarta, GianfrancaNguyen, WilliamCartagena-Rivera, AlexanderNicholas, AnthonyCarter, WayneNichols, Buford L.Cartland, SianNicolás, Francisco E.Carullo, GabrieleNicoletti, ClaudioCaruntu, ConstantinNicoletti, FerdinandoCaruso Bavisotto, CelesteNicolia, AlessandroCaruso, CalogeroNicolini, AndreaCaruso, CarlaNicosia, AldoCaruso, GerardoNie, JinleiCaruso, GiuseppeNiedbała, GniewkoCaruso, SalvatoreNieddu, GiovanniCarvajal, Asia FernandezNiederman, Robert A.Carvajal, MicaelaNiedziela, TomaszCarvalho Silva, JorgeNiedźwiedzka-Rystwej, PaulinaCarvalho, CristinaNiedzwiedzki, DariuszCarvalho, JoanaNiehaus, Thomas DCarvalho, LitiaNielsen, Vance G.Carvelli, LuciaNielsen, Vance GirardCarver, ChaseNieman, David C.Casacuberta, ElenaNiemirowicz-Laskowska, KatarzynaCasalino, ElisabettaNiero, GiovanniCasalou, CristinaNiessen, H.W.M.Casamassimi, AmeliaNiessen, LudwigCasanova, Emily LNieto, Teresa PérezCasanova, LlanosNieus, Thierry R.Casanova-Sáez, RubénNieveen Van Dijkum, ElisabethCasas, Ana M.Niewiadomska, EwaCasella, LuigiNifli, Artemissia-PhoebeCash, Elizabeth I.Nighot, PrashantCasimiro, Maria HelenaNigris, Filomena DeCasini, GiovanniNiikura, MasahiroCasolo, ValentinoNikas, Spyros P.Caspari, ThomasNikiforov, AndreyCassano, DomenicoNikodemova, MariaCassidy, Pamela BondNikoh, NaruoCastagna, MichelaNikolaev, EugeneCastandet, BenoitNikolaev, ViacheslavCastanera, RaúlNikolaidis, NikolaosCastelli, M. PaolaNikolaidis, NikolasCastelli, VanessaNikolakaki, EleniCastillo-Gonzalez, Claudia MarcelaNikolakopoulou, Angeliki M.Castñeiras, AlfonsoNikolay, RainerCastoldi, ElisabettaNikulchev, EvgenyCastoli, AngelaNiland, StephanCastoria, GabriellaNilsson, HenrikCastorina, AlessandroNilsson, MariaCastorina, GiuliaNilvebrant, JohanCastren, MaijaNirala, NirajCastriconi, RobertaNishi, KosukeCastro, María ÁngelesNishi, MayumiCasu, CarlaNishida, NaokiCatalano, AntoninoNishida, YuichiroCatanzano, OvidioNishigaki, TakuyaCatanzaro, GiuseppinaNishiguchi, MasamichiCatauro, MichelinaNishihara, HidenoriCaterino, MariannaNishihara, MasamichiCato, Andrew C. B.Nishikawa, ToruCattaneo, FabioNishimoto, KoshiroCattò, CristinaNishimura, MasahiroCatucci, LuciaNishimura, WataruCaudle, MichaelNishiuchi, TakumiCauli, OmarNishiyama, SoichiroCava, ClaudiaNishiyama, YusukeCavalcanti, João Henrique F.Nishizawa, YokoCavallaro, UgoNishizuka, MakotoCavalu, SimonaNisiotou, AspasiaCazzato, EugenioNisnevitch, MarinaCebova, MartinaNistala, RaviCeccarelli, GabrieleNitsos, Christos K.Ceccarelli, MatteoNitta, TakeshiCecchetti, SerenaNitulescu, George MihaiCecchin, ErikaNitulescu, MihaiCécile, OuryNiu, DongyanCefalas, A.C.Nixon, Daniel W.Celetti, AngelaNizhnikov, Anton A.Celia-Terrassa, ToniNjar, Vincent C.O.Celiński, KonradNocentini, GiuseppeCellek, SelimNociari, MarceloČėnas, NarimantasNoda, MakotoCenci, GiovanniNoda, NaonobuCentonze, DiegoNoda, ShinjiCeolotto, GiulioNoël, AlexandraCERALINE, JocelynNofer, Jerzy-RochCeranowicz, PiotrNoghero, AlessioCereda, MatteoNoguchi, MasayukiCeresa, BrianNogué, FabienČerná, MarieNoguera, Nélida InésCernera, GustavoNoh, Ji-yoonČerný, JiříNoh, KeumhanČerný, MartinNohe, AnjaČeřovský, VáclavNomiyama, TakashiCerrada, ElenaNomura, SeitaroCeruti, StefaniaNonami, AtsushiCerutis, RoselynNonnekens, JulieCervino, GabrieleNonogaki, KatsunoriCesareo, RobertoNonomura, Ken-ichiCestmir, AltanerNoordergraaf, Inge-WillemCha, ChaenyungNor Eddine, SounniChacko, Jenu VargheseNordgren, Tara M.Chae, HeeyoungNoren Hooten, NicoleChae, Jung-wooNorman, TrevorChae, YounbyoungNorris, ErinChaen, ShigeruNorton, Kerri-AnnCháfer-Pericás, ConsueloNorton, NadineChaganty, BharatNosoudi, NasimChagraoui, AbdeslamNotaras, Michael J.Chai, Han-haNotarnicola, MariaChaki, MouniraNou, Ill-SupChakrabarti, PriyadarshiniNovack, Gary D.Chakrabarti, SubhadeepNovak Kujundžić, RenataChakraborty, AbhijitNovak, BelaChakraborty, SayanNovák, JanChakravarty, ShatadruNovič, MarjanaChakravorty, DavidNovick, DanielaChalla, DilipNovinec, MarkoChallagundla, KishoreNovío, SilviaChallet, EtienneNovotna, AlzbetaChamakura, KarthikNowak, AdrianaChambers, Jeremy W.Nowak, DorotaChamulitrat, WaleeNowak, KatarzynaChan, Ben C. L.Nowak-Sliwinska, PatrycjaChan, Hon FaiNowicka, GrażynaChan, SunneyNowinska, KatarzynaChan, Yin-ChingNowotarski, ShannonChand, VaibhavNowotny, NorbertChandrasekar, IndraNozaki, MasamiChandrasekaran, MurugesanNozu, KandaiChandrashekhar, KshipraNR, ParineChang, Audrey N.Ntatsi, GeorgiaChang, Cheng-ChangNtie-Kang, FideleChang, Chia-cheNuessler, AndreasChang, Chia-ChenNumakawa, TadahiroChang, Chien-ChungNuñez, PaulaChang, Ching-yaoNunziata, AngelinaChang, Hai-ChouNüsken, EvaChang, Hsin-I.Nüssler, Andreas K.Chang, Hsueh-WeiNuţǎ, Diana CameliaChang, Hui-huaNuthikattu, SaivageethiChang, HyunNwaiwu, OgueriChang, Jia-FengNylander, KarinChang, Kee-lungNzau Matondo, BillyChang, Li-ChingO’Brien, EdwardChang, Ling-ChuO’Connor, DanielChang, Long-SenO’Donoghue, PatrickChang, MeichiO’neill, Catherine A.Chang, Nai-JenObara-Michlewska, MartaChang, PengxiangObata, HideakiChang, TayuanObermeier, BirgitChang, Te-ShengObermüller, NicholasChang, Theresa Li-yunObinata, DaisukeChang, Wen-WeiObrador, ElenaChang, Yu-ChaoOchi, ShinichiroChang, Yu-JiaOchiai, BungoChang, Yu-WeiOchoa-Reparaz, JavierChankhamjon, PranatchareeyaOchsenreiter, TorstenChantana, JakapanOcker, MatthiasChao, Wen-WanOćwieja, MagdalenaChapple, SarahOda, YukariChaptal, VincentOdendall, CharlotteChardot, Thierryodenthal, margareteCharkowski, AmyOdintsova, Tatyiana I.Charles Jacob, Harrys KishoreOdland, JonCharles Richard, John LalithOdle, Angela KatherineCharles, PhilipO’Donoghue, PatrickCharmsaz, SaraOeckinghaus, AndreaCharriaut-Marlangue, ChristianeOertelt-Prigione, SabineChase, P. BryantOffer, StevenChatonnet, ArnaudOffermanns, VincentChatterjea, DevananiOfir, RivkaChatterjee, AbhijitOgai, KazuhiroChatterjee, ShampaOgata, YoshiyukiChatterjee, SubrotoOgawa, AkikoChatterjee, SudiptaOgawa, TakumiChattopadhyay, SaurabhOgi, KazuhiroChatzaki, EkateriniOgino, ShujiChatzellis, EleftheriosOgórek, RafałChatzistavrou, XanthippiOgungbe, IfedayoChauhan, NeerajOgunjirin, AdebowaleChauveau, LiseOh, Eun-jeeChaves, ElenaOh, JaegyunChaves-Martinez, Felipe JavierOh, JisunChazot, PaulOh, Jung HunCheccucci, AliceOh, YohanCheemarla, Nagarjuna ReddyO’Hagan, HeatherCheleschi, SaraOhashi, NamiChen, ChiOhba, ShinsukeChen, Chih-ChengOhe, KenjiChen, ChuanruiOhko, KentaroChen, Chun-ChiOhkuri, TakayukiChen, Chung-HwanOhl, KimChen, DaiqinOhman-Magi, CarolineChen, GangOhmido, NobukoChen, Grace QOhmoto, AkihiroChen, Guang-ChaoOhneda, KinukoChen, Gwo-ShingOhnishi, MasatoshiChen, H. IsaacOhno, Shin-ichiroChen, HongOhno, ShoChen, Hong-HwaOhshimo, ShinichiroChen, HouyangOhta, ShinjiChen, Jen-TsungOhta, YukoChen, JinbiaoOhtaka, AtsushiChen, JingOhtomo, RyoChen, Ji-YihOhya, SusumuChen, Jyh-PingOhyama, A.Chen, Kai-YunOhyama, TakujiChen, Ko FanOikawa, TsunekazuChen, Kuan-FuOjala, JohannaChen, Kuo-YuOkada, MotohiroChen, LeileiOkada, MuneyoshiChen, LiamOkada, TaketoChen, LiangOkada, TomoyoChen, Lih-ChyangOkada, YasunobuChen, LinOkajima, HideakiChen, LisongOkamoto, MasamiChen, LiyeOkamoto, TakayukiChen, MingOkazaki, JojiChen, MingshengOkazawa, AtsushiChen, MingzhouOkinaga, ToshinoriChen, Pei-WenOkino, TatsufumiChen, QiOklestkova, JanaChen, Rong-JaneOkochi, HitoshiChen, Sean Chun ChangOk-Seon, KwonChen, Shih-YuanOku, MasahideChen, Shuen-EiOkumura, NobuakiChen, SuzieOlaru, Octavian TudorelChen, TaipingOlas, Justyna JadwigaChen, Wei-ChuanOlcese, JamesChen, Wei-ShengO’Leary, Brendan M.Chen, Wei-YuO’Leary, SeánChen, WenchunOlech, MartaChen, Wen-YingOlejníková, PetraChen, XiaoyanOliva, AlessandraChen, XinchunOliva, JoanChen, XingjuanOliva, RominaChen, YanaOliveira, Ana M.M.Chen, Yi-JenOliveira, HugoChen, YingOliveira, Maria ConceiçãoChen, Yi-WenOliveira, Mariana BragaChen, Yu-ChunOliveira-Garcia, ElyChen, Yun-JuOlivera, AnaChen, Yun-WenOliverio, ManuelaChen, ZheOlivos-Oré, Luis AlcidesChénais, BenoitOlkkonen, Vesa M.Cheng, Chih-ChiaOllero, MarioCheng, DeOlmeda, BarbaraCheng, Fong-YuOlova, NellyCheng, HailiOlsen, Christopher M.Cheng, HaoOlson, HeatherCheng, Heung-ChinOlsson, SannaCheng, Hung-ChiOltra, ElisaCheng, JiaOmagari, KatsuhisaCheng, Juei-tangOmar, AhmadCheng, Jya-WeiOmar, Syed HarisCheng, Kuang-HungOmidian, KosarCheng, LinOmote, HiroshiCheng, LishengOnali, PierluigiCheng, Shih-PingOnčák, MilanCheng, XiOndrej, VladanCheng, XiangO’Neal, HollisCheng, XiaomingO’Neal, MatthewCheng, XinlaiO’Neal, ScottCheng, Yuen YeeOng, QunxiangCheng, ZheOng, Sang BingChenoll, EmparOngkeko, Weg M.Cheon, Yong-PilOniga, IlioaraCheong, HeesunOniszczuk, AnnaCheong, Mi SunOno, NoriakiCheong, Yong HwaOno, TakashiChereddy, KiranOnyango, IsaacChérel, IsabelleOo, Win MinCherkaoui Malki, MustaphaOoi, KennethCherkasov, ArtemOon, Hazel H.Chermnykh, ElinaOono, YoukoChetwynd, Andrew J.Oostenbrink, ChrisCheung, Wing-HoiOosterwijk, EgbertCheungpasitporn, WisitOosting, JanChevalier, FrançoisOpaliński, ŁukaszChevet, EricOpattová, AlenaChhabra, GaganOpazo, CarlosChhunchha, BhavanaOponowicz, AgnieszkaChi, Celestine N.Oracz, JoannaChi, GeraldOrcesi, SimonaChiang, Hsiu-MeiOrekhov, Alexander N.Chiang, John (Pei-Wen)Orelle, CédricChiang, Meng-TsanOrend, GertraudChiang, Vincent L.Orian, LauraChiang, Yi-TingOrii, Kenji E.Chiapella, JorgeOrimo, AkiraChiarle, RobertoOrlandi, AugustoChiefari, EusebioOrlov, SergeiChien, Huan-ChiehOrlov, YuriyChien, JeremyOrmeci, AlimChien, Wade W.Ornoy, AsherChien, Yin-HsiuO’Rourke, JamieChiesa, EnricaOroz-Guinea, IsabelChifiruc, Mariana CarmenOrr, BernardoChihara, NorioOrrego, SantiagoChijiwa, TakahitoOrso, EvelynChim, Shek ManOrsolic, NadaChimenti, IsottaOrtega Avila, Ana BelenChin, Kok YongOrtega-Loayza, AlexChin, Young-wonOrth, James D.China, ArnabOrtiz, CarmenChini, AndreaOrtiz, J. BryceChino, MarcoOrtolano, SaidaChiocchetti, AnnalisaOrton, Thomas J.Chipman, Ariel D.Orynbayeva, ZulfiyaChiquet, MatthiasOrzan, FrancescaChirumbolo, SalvatoreOrzechowski, ArkadiuszChitraju, ChandramohanOrzelska-Górka, JolantaChitrala, Kumaraswamy NaiduOsada-Oka, MayukoChittori, SagarOsera, CeciliaChitu, VioletaOshima, YusukeChiu, Chi-chengOsipov, VladimirChiu, JoannaOsorio, Elvia JanethChiu, Norman H. L.Osredkar, DamjanChiu, Tsai-HsinOssoli, AliceChiumello, DavideOstacolo, CarmineChiurazzi, MaurizioOstersetzer-Biran, OrenChlichlia, KaterinaOstolaza, HelenaChmurzyńska, AgataOstrowska, AgnieszkaCho, HannaOstrowski, DanielaCho, Hea-YoungO’Sullivan, Jeremy A.Cho, Hyeon-YeolOswald, StefanCho, Jae YoulOszako, TomaszCho, JeongheeOszust, KarolinaCho, Je-YoelOta, HiroyoCho, Man HoOthman, Eman M.Cho, NamhoonOtlewski, JacekCho, SiHyunOtsuka, YuzuruCho, Tae-JuOtsuki, TakemiChobot, VladimírOtto, ThomasChoi, Chang WonOtulak-Kozieł, KatarzynaChoi, DonghoonOtzen, DanielChoi, Jane RuO-Uchi, JinChoi, SangchunOuro, AlbertoChoi, Yeung JoonOwen, CarolynChoi, Yoon KyungOwens, GregoryChoi, YoukyungOxford, JuliaChong, Parskson Lee GauOza, JavinChopra, RatanOzaki, IwataChou, KarenOzaki, ToshinoriChou, Lan-SzuOzaltin, KadirChou, Shih-FengOzawa, ShogoChou, Tsung-HanOzeki, YasuhiroChou, YachangOzen, MehmetChoudhuri, AvikOzen, MustafaChoukroun, JosephOzsvari, BelaChow, Franklin Wang-NgaiP. Morgado S. Neves, Maria Da GraçaChow, JamesPaavola, JereChow, Simon Kwoon-HoPabbidi, Reddy MChowański, SzymonPacak, AndrzejChowdhury, IndrajitPacak, ChristinaChoy, Francis Y.M.Pace, FabioChrist, BrunoPace, PaulChristensen, AlanPace, VittorioChristians, JulianPaci, MaurizioChristman, GregoryPacifico, DanielaChristoffers, MichaelPacifico, SeverinaChristophersen, ClausPacios, Luis F.Christov, Nikolai K.Packiam-Dayalan, AntonyChrobak, ElwiraPadalkar, SonalChruszcz, MaksymilianPadilla-Benavides, TeresitaChrysanthakopoulos, Nikolaos A.Padmanabhan, JagannathChtarbanova, StanislavaPadmanabhan, PriyaChu, Fang-huaPaduch, RomanChu, KonPadula, Matthew P.Chu, Pei-MingPae, Eung-KwonChu, Shi-JyePaek, NamchonChu, Tin-ChunPaek, Seung-mannChu, XianpingPaeschke, NadineChuang, Chin-kaiPaeshuyse, JanChuang, Shih-YiPagán, IsraelChubanov, VladimirPagani, FrancoChueh, Anderly C.Pagani, StefaniaChueh, Pin JuPagano, AlessandraChul-sung, HuhPagano, StefanoChun, RenePage, CliveChun, SungkunPage, Richard C.Chung, Byung MinPageau, KarineChung, DonghoonPagliai, Fernando A.Chung, GyuhwaPagliarani, ChiaraChung, HenryPagnotta, Mario A.Chung, Jay H.Pagnussat, GabrielaChung, Man-KyoPagonopoulou, OlgaChung, SungjinPahwa, RomaChung, Tae NyoungPaik, JisunChuntao, ZhaoPaino, FrancescaChuu, Chih-PinPakhomov, AndreiChwastek, JakubPal Choudhuri, ShreoshiChyan, Chia- LinPal, KasturiChyau, Charng-CherngPál, MagdaCiaccio, MarcelloPalacios, José-ManuelCiaglia, ElenaPalacios-Santander, José MaríaCianci, AntonioPaladino, SimonaCiancio, AurelioPalamara, Anna TeresaCianciosi, DanilaPalani, KirubakaranCiani, BarbaraPalaniappan, BalasubramanianCiardelli, FrancescoPalanisamy, ArulselvanCiarimboli, GiulianoPałasz, ArturCiaudo, ConstancePalazzo, EnzaCiccarelli, MichelePaleari, LauraCicciù, MarcoPalermo, FrancescoCicènas, SauliusPalermo, GiuliaCicero, ArrigoPalestini, PaolaCichero, ElenaPaliyath, GopinadhanCichocki, FrankPallaoro, AlessiaCichorek, MiroslawaPallavi, ManralCiciliot, StefanoPalle, KomaraiahCid, AngelesPallier, VirginieCieniewski-Bernard, CarolinePallottini, ValentinaCiereszko, IwonaPalma, JoséCiesiołka, JerzyPalma, Paulo JCieślińska, AnnaPalmer, Nathan A.Cignarella, AndreaPalmerini, Carlo AlbertoCil, OnurPalmerini, MariaCiliberti, Maria GiovannaPalmieri, CamilloCimmino, FloraPalmieri, LuisaCimpean, Anca MariaPalmieri, OrazioCîmpean, AnişoaraPalmieri, ValentinaCindrova-Davies, TerezaPalmisano, AlidaCinelli, PatriziaPal-Nath, DipasmitaCinteza, Otilia LudmilaPalomba, LetiziaCione, ErikaPalomo Ríos, ElenaCipak Gasparovic, AnaPalumbo, CarlaCipak, LubosPalumbo, PaolaCipollo, JohnPaluri, RaviCiribilli, YariPamarthy, SahithiCirillo, MarcoPamir, NathalieCiszewski, Wojciech MichałPan, Hung-ChuanCitek, JindrichPan, JingCitro, SimonaPan, MiaoCittaro, DavidePan, MingguiCiuffreda, LudovicaPan, PanCivera, Maria ConcepciónPan, Tai-longCividini, FedericoPan, W. DavidClaing, AudreyPan, XiaoyueClanchy, FelixPan, YangangClaridge, Jolyon KPan, YongchuClark, AndyPanara, FrancescoClaros, EdithPanaro, Maria AntoniettaClaus, Ralf A.Panasiewicz, GrzegorzClausen, JohannesPanatala, RadhakrishnanClavijo-McCormick, AndreaPancsa, RitaClement, CristinaPanda, AmareshClement, SophiePanda, Amaresh C.Clerico, AldoPandey, Amit V.Cleveland, Beth M.Pandey, Atul KumarCliment Salarich, MontserratPandey, Avinash ChandraCliment, MontserratPandhi, AbhiClotet, SergiPandith, AnupClyne, MargueritePandolfi, AssuntaCmielewski, PatriciaPandolfini, TizianaCobellis, GildaPanek, JaroslawCocchi, MassimoPanek, RafalCoccia, ElenaPanfoli, IsabellaCoccurello, RobertoPang, BoCochrane, Dawn RPANGA, JAIPALREDDYCodacci-Pisanelli, GiovanniPangestu, MulyotoCoelho, ConstançaPanickar, KiranCohen, BoikoPankratova, StanislavaCohen, DavidPannaccione, AnnaCohen, GeraldPannerselvam, SureshCohen, GuyPantalone, Vincent R.Cohen, MichaelPantea Stoian, AncaCoirault, CatherinePanteleev, MikhailColanzi, AntoninoPanteris, EmmanuelColdea, TeodoraPanthee, DilipCole, JeffPantziarka, PanColecchia, MaurizioPanyutin, IgorColeman, CynthiaPanzarasa, GuidoColeman, Jonathan A.Panzenboeck, UteColeman, Mitchell C.Panzer, SimonColeman, NicholaPaolicelli, PatriziaColgan, Donald JPaolmino, OlgaColicchio, JackPaolocci, NazarenoCollado-González, MarPapa, AntonellaCollavin, LicioPapaccio, FedericaCollawn, James F.Papaccio, GianpaoloCollett, AndrewPapadopoulos, Antonios N.Collier, JasonPapageorgiou, Anastassios C.Collins, Fraser L.Papageorgiou, AristotelisCollins, GretchenPapageorgis, PanagiotisCollinson, Lucy MPapagiannopoulos, AristeidisCologna, StephaniePapakyriakou, AthanasiosColombo, EmanuelaPapamatheakis, Joseph (Sifis)Colon Saez, JosePapantoniou, IoannisColonna, GiovanniPapavassiliou, Athanasios GColuccia, MauroPapazafiropoulou, AthanasiaColzi, IlariaPapini, EmanueleComai, StefanoPaplinska, MagdalenaComan, CristianPappa, AglaiaCombet, EmiliePapp-Wallace, Krisztina M.Comi, CristoforoParadiso, PatriziaCominelli, EleonoraParamythiotis, SpirosCommault, Audrey S.Paranjape, AnuragCommisso, MauroParashar, DeepakComstock, Lindsay R.Paraskeva, EfrosyniConde, CarlosParaskevas, George P.Conde, JavierParayath, NehaCondello, MariaParcells, MarkCondorelli, Daniele FilippoParchem, RonaldConesa, Juan A.Pardo, Jose M.Conese, MassimoPardo, MaríaCongestri, RobertaParent, RomainConigliaro, AliceParfenova, LyudmilaConsole, LaraParfitt, GeraintConstant, SamuelParfrey, HelenConstantinos, PapagiannitsisParikesit, Arli AdityaContaldo, NicolettaParis, JuanConte, MarcoParis, Thomson M.Conti, BicePark, Chan HoConti, LucioPark, Chan YoungConti, PioPark, DaehoContino, MarialessandraPark, Dong SunContreras, CristinaPark, EnochContreras, OsvaldoPark, Jang-SuContreras, Rubén G.Park, Jong KookConway, Daniel E.Park, JoongkyuCook, KristinaPark, JoshuaCooke, Bradley M.Park, Jun-BeomCooney, RalphPark, JunseongCooper, RobinPark, JunyongCooper-Knock, JohnathanPark, Ky YoungCopolovici, Dana MariaPark, KyeongsoonCopolovici, LucianPark, Kyong-CheulCorazzari, MarcoPark, Kyoung-ChanCordani, MarcoPark, Myoung HeeCordaro, MarikaPark, Sae WoongCorella, DoloresPark, Sang-WookCorfield, Anthony P.Park, Seung-ChunCormio, AntonellaPark, Si JaeCornelius, DenisePark, Sung-GyooCornillet, MartinPark, TaehwanCorniola, RikkiPark, TaesunCorrado, AddolorataPark, YongsooCorrado, ChiaraPark, Young-SeoCorrado, GiandomenicoParkar, Shanthi G.Corrales-García, JoelParkkita, SeppoCorral-Martínez, PatriciaParkman, HenryCorrea, Maria ElviraParl, FritzCorreia Coelho, Ana CláudiaParnandi, AvinashCorreia, Carlos M.Parodis, IoannisCorrochano, SilviaParolini, CinziaCorsi, LorenzoParonetto, Maria PaolaCortese, BarbaraParra-Guillen, Zinnia PatriciaCosco, DonatoParreira, RicardoCosentino, MarcoParrella, PaolaCosenza, MariaParrino, BarbaraCosme, MarcoParrino, VincenzoCoss, ChristopherParry, Traci L.Costa, Ana RitaParsanathan, RajeshCosta, Anna MariaParsonage, DerekCosta, HugoParsons, LindaCosta, JoanaPartch, Carrie L.Costa, JuliaPartearroyo, TeresaCosta, MarinaPasantes, JuanCosta, Paulo JorgePascale, RosaCosta, RuiPascarella, StefanoCosta, ValerioPascual, María BelénCosta, Vera MarisaPascual-Teresa, SoniaCosta, VivianaPasetto, PamelaCosta-Almeida, RaquelPashikanti, SrinathCosta-Bauzá, AntòniaPashov, AnastasCostantini, SusanPasqua, LuigiCosta-Pinto, Ana RitaPasqualoto, KerlyCosta-Silva, BrunoPasquinelli, GianandreaCostelli, PaolaPasternak, TarasCougnoux, AntonyPasternak, Taras P.Courtois, GillesPastor, FernandoCousins, Asaph B.Pastores, Gregory M.Coussons, PeterPašukonienė, VitaCoutant, FrédéricPatching, SimonCoutinho, Maria FranciscaPátek, MiroslavCoutinho, PaulaPatel, AlokCouvineau, AlainPatel, BhargavCovello, GiuseppinaPatel, DaksheshCovino, RobertoPatel, DevenCowan, JuthapornPatel, HardikkumarCowardin, CarriePatel, NilkanthCowman, MaryPatel, NrupaliCox, Linda S.Patel, Vaibhav B.Cozzi, CristinaPathak, DhrubaCraig, Andrew W.Patil, AbhinandanCraige, Siobhan M.Patil, Swati J.Cram, Erin JPatini, RomeoCrans, Debbie C.Paton, ChadCrawford, LindseyPatrauchan, Marianna A.Creamer, RebeccaPatrice, ThierryCrehuet, RamonPatrizi, BarbaraCremers, FransPatrizia, NittiCrescenzi, MarcoPatro-Małysza, JolantaCrescioli, ClaraPattammattel, AjithCrespan, EmmanuelePattanaik, SitakantaCrestani, MaurizioPattappa, GirishCreusot, RemiPatterson, Eric LCreuzenet, CarolePatterson, JenniferCrevillén, PedroPaudel, Keshav RajCridge, AndrewPaul, AnanyaCripe, Linda H.Paul, Narayan ChandraCrisan, LuminitaPaulo, AntónioCristian, ChaparroPautz, AndreaCrnkovic, AnaPauwels, PatrickCrocetti, LetiziaPavic, ValentinaCrocini, ClaudiaPavlin, MaticCroitoru, CatalinPavlov, DanailCronauer, Marcus V.Pavlov, Georges M.Crosas, BernatPavlov, Youri ICrosatti, CristinaPavlova, ElenaCrucho, CarinaPavlovič, AndrejCruickshank-Quinn, CharmionPavo, NoemiCrus-Topete, DianaPavon-Djavid, GracielaCruz-Rubio, José ManuelPawar, KamleshCsapo, EditPawar, PrashantCséfalvay, EditPawar, ShrikantCséplő, ÁgnesPawelek, JohnCsordas, AndrewPawlik, AndrzejCsordas, GyorgyPawlik, AnnaCsősz, ÉvaPayne, AnnetteCuajungco, MathPazienza, ValerioCuburu, NicolasPazos-Pérez, NicolásCucchiarini, MagaliPazzagli, LuigiaCucinotta, FrancisPeana, Massimiliano F.Cucullo, LucaPearce, Amanda KCuddy, WilliamPearce, StephenCui, ChuanlongPechanova, OlgaCui, HaissiPeche, VivekCui, RongfengPécheur, Eve-IsabelleCui, TiantianPeck, BarrieCui, XiaoyingPecori, RiccardoCui, YePedemonte, NicolettaCull, BenjaminPedersen, Finn SkouCunha, Rodrigo A.Pedersen, HenrikCunningham, CatrionaPedersen, Per AmstrupCunningham, Janet L.Pedeux, RémyCuppoletti, JohnPedraza Chaverri, JoséCurcio, Christine A.Pedrero, MariaCurrie, MargaretPeelman, FrankCurrie, SusanPeeters-Scholte, CachaCurtis, DavidPeguero-Pina, José JavierCurtis, MarionPeichl, LeoCusanelli, EmilioPeinado, María ÁngelesCusick, Matthew F.Peinemann, FrankCuspidi, CesarePeirats-Llobet, MartaCutler, Mary LouPeiró, SandraCutone, AntimoPeitzsch, ClaudiaCyganek, LukasPejoski, DavidCytlak, UrszulaPekkala, SatuCzaja, KrzysztofPelagalli, AlessandraCzaplewski, CezaryPelin, MarcoCzarnocka, WeronikaPellegatta, SerenaCzerwinska, MonikaPellegrini, GaiaCzikora, IstvanPellegrino, DanielaCzogalla, KatrinPellet-Rostaing, StéphaneCzyż, JarosławPellettieri, JasonCzyz, MalgorzataPelliccia, FrancaCzyżewski, KrzysztofPellis, AlessandroD. Bortner, CarlPellois, Jean-PhilippeD´Souza, MalcolmPelosi, GiorgioD’Agosta, RobertoPeluso, IlariaD’Agostino, DonatoPeluso, Marco E. M.D’Argenio, ValeriaPemp, BertholdD’Elia, LanfrancoPena, Maria MarjoretteD’Orazi, GabriellaPenchev, HristoDa Costa Martins, PaulaPendin, DianaDa Costa, Ana RosaPenfornis, PatriceDa Costa, Rui M. GilPeng, Anthony W.Da Silva, Jorge MarquesPeng, ChaoDa Silva, Marta AlvesPeng, ChenDa Silva, Mateus WebbaPeng, HanDa Silva, Robin P.Peng, HuiDa Silva, Ronaldo LuisPeng, Shu-FenDabrowiak, JamesPeng, TengDacarro, GiacomoPeng, Xianlu LauraDacheux, Jean-LouisPeng, Yi-JenDachineni, RakeshPeng, Ying-JieD’Agostino, PaulPeng, ZeDahan, AlbertPenin, AlekseyDahl, Jan-UlrikPenke, BotondDahms, Hans-UwePenn, LindaDai, ShaojunPennock, NathanDai, YuminPentzold, StefanDai, ZhaohuaPenzo, MariannaDaimi, HouriaPeppelenbosch, M.P.Daimiel, LidiaPerale, GiuseppeD’Aiuto, FrancescoPercesepe, AntonioDakhama, AzzeddinePerche, FedericoDal Ben, MatteoPercy, Alan KDal Col, JessicaPerdih, AndrejDalby, AndrewPerego, CarlaDale, ReneePerego, ClaudioDaleau, PascalPereira, Ana LuisaD’Alfonso, LauraPereira, Carlos DiasDalianis, TinaPereira, DavidDalla Pozza, ElisaPereira, FlorbelaDall’Acqua, StefanoPereira, Maria CarmoDam, JuliePereira, RubenDama, MuraliPerera, Agampodi PromodaD’Ambrosio, ChiaraPerevalov, Timofey ViktorovichDamia, GiovannaPérez Alvarez, Maria JoséDamiani, DanielaPerez Pascual, DavidD’Amico, Agata GraziaPérez Sánchez, HoracioDammermann, WernerPerez, GuillermoDamm-Welk, ChristinePérez, Karel TalaveraD’Amora, UgoPérez-Álvarez, LeyreDams-Kozlowska, HannaPérez-Fernández, MaríaDandekar, AdityaPérez-García, SeleneDandekar, ThomasPérez-Hernández, MartaDando, SamanthaPérez-Martín, FernandoD’andrea, CristianoPérez-Montaño, FranciscoDane, FennyPérez-Ortiz, José M.Danelli, LucaPérez-Pascual, DavidDanesi, RomanoPérez-Sen, RaquelDaniele, AuroraPerez-Serrano, MartinaDanilenko, MichaelPerez-Siles, GonzaloDanisovic, LubosPergament, EugeneDanková, ZuzanaPérigo, Élio AlbertoDansette, Patrick M.Periyasamy, PalsamyDAnte, SilviaPerkins, AndrewDanti, SerenaPerl, AndrasD’Antonio, Edward L.Permyakov, EugeneDar, Wasim A.Perni, MicheleDaras, GerasimosPernisová, MarkétaDarbani, BehroozPernomian, LaenaDarbin, Olivier EricPero, RaffaelaDarbon, PascalPeronnet, FrederiqueDarcy, JustinPerov, NikolayD’Arcy, PadraigPerrella, GiorgioD’Argenio, ValeriaPerricone, CarloDarie, Costel C.Perrier, Jean-FrancoisDarling, AprilPerrine-Walker, FrancineDarvell, BrianPerron, HervéDarvell, Brian W.Perrone, GiancarloDas Gupta, SanatanPerrone-Bizzozero, Nora I.Das, AninditaPerrotta, FabioDas, SadhanPerry, RachelDas, SubhaPerry, TrentDasari, SurendraPersson, EmmaDasgupta, SantanuPerteghella, SaraDash, BirajaPerticone, FrancescoDastsooz, HassanPerucca Orfei, CarlottaDaszkowska-Golec, AgataPerucha, EsperanzaDatta, KausikPerucka, IrenaDatta, PoulamiPeruzzotti-Jametti, LucaDatta, Proteomic ArnabPervouchine, DmitriDaubon, ThomasPešić, MilicaDaugelavicius, RimantasPeška, VratislavDaverey, AmitaPestov, AlexanderDavid, LaurePestov, DimitriDavid, RobertPeter, Emanuel K.David-Cordonnier, Marie-hélènePéter, HegyiDavidson, MichaelPeter, MartinDavie, AndrewPeters, DonnaDavies, ChristopherPeters, JudithDavis, AdamPeters, StefanDavis, CatherinePetersen, Robert B.Davis, JeffPetit, Patrice X.Davis, Keith R.Petitot, Anne-SophieDavis, Matthew C.Petitte, JimDavis, Michael E.Petkov, StoyanDawood, Mahmoud A.O.Petkova, NadezhdaDawson, JonathanPetráková, VladimíraDAZA NAVARRO, PAULAPetralia, Maria CristinaDe Almeida, AndreiaPetre, BenjaminDe Amici, MarcoPetrie-Hanson, LoraDe Angelis, AntonellaPetrikaite, VilmaDe Aza, Piedad N.Petrillo, SaraDe Bellis, LuigiPetrinovic, Marija-MagdalenaDe Benedetto, AnnaPetroni, KatiaDe Berardis, DomenicoPetropoulos, Spyridon ADe Bock, MarijkePetrou, Athinoula L.De Boer, HugoPetrova, Krasimira T.De Brevern, Alexandre G.Petry, Clive J.De Cáceres González, Francisco Federico NunezPetta, IoannaDe Carlo, FlaviaPettersen, CarolineDe Chiara, LetiziaPettinato, GiuseppeDe Chiara, LorettaPezzi, VincenzoDe Cózar, AbelPezzuto, AldoDe Craene, Johan-OwenPfaender, StephanieDe Croft, Priyakshi KalitaPfeffer, GeraldDe Curtis, FilippoPfeffer, MartinaDe Deurwaerdère, PhilippePfrieger, FrankDe Falco, ElenaPham, ThaoDe Falco, ValentinaPham, Tien DucDe Felice, MarioPhan, Thi Tuong VyDe Felici, MassimoPhelps, Mitch A.De Filippis, VincenzoPhesse, TobyDe Francesco, ErnestinaPhilippe, NaquetDe Freitas Fraga, Hugo PachecoPhillips, JoshuaDe Freitas Germano, JulianaPhillips, TycelDe Frutos, Pablo GarcíaPhipps, Amanda I.De Geest, BartPhulera, SwastikDe Giglio, ElviraPiaggio, GiuliaDe Graaf, CoenPiantanida, IvoDe Gracia, PabloPiao, XijunDe Jong, StevenPiatkevich, Kiryl D.De La Fuente, HortensiaPiazza, OrnellaDe La Grange, Philippe Brunet Picard, DidierDe La Herrán, RobertoPiccialli, VincenzoDe La Lastra, José Manuel PérezPiccinini, AnnaDe La Luz Garcia-Hernandez, MariaPiccionello, Antonio PalumboDe La O Leyva Pérez, Maria Pichler, IreneDe La Torre, Jose GarciaPicimbon, Jean-FrançoisDe Lange, PieterPickering, RaeleneDe Las Heras, JavierPickett, HildaDe Lise, FedericaPicking, William D.De Lorenzo, Mariana SPiconi, CorradoDe Luca, PaolaPiecha, GrzegorzDe Maagd, RuudPiechowicz, LidiaDe Martin, SaraPiedras, PedroDe Masi, LuigiPiegat, AgnieszkaDe Masi, RobertoPiel, William HDe Matos Simoes, RicardoPiemonte, FiorellaDe Matteis, RitaPiemontese, LucaDe Matteis, ValeriaPieniazek, AnnaDe Mieri, MariaPiepenbrink, KurtDe Molina, Paula MaloPieraccini, StefanoDe Oya, Nereida JiménezPiergiovanni, Angela R.De Palma, RaffaelePierno, SabataDe Pinto, VitoPietrowska-Borek, MałgorzataDe Re, VallìPietrzak, AldonaDe Robertis, MariangelaPignata, ClaudioDe Rosa, Maria CristinaPignataro, GiuseppeDe Rosa, VivianaPignatti, PatriziaDe Rossi, AnitaPiiper, AlbrechtDe Ruyck, JérômePikridas, MichaelDe Servi, StefanoPikuła, MichałDe Smaele, EnricoPilarsky, ChristianDe Sousa-Coelho, Ana LuisaPilc, AndrzejDe Stefano, RosaPiliponsky, AdrianDe Toni, LucaPiliponsky, Adrian M.De Totero, DanielaPilipovic, AnaDe Vecchis, RenatoPillozzi, SerenaDe Vega, José Miguel LizcanoPilot, GuillaumeDe Vicente, Juan CarlosPinalli, RobertaDe VIENNE, DominiquePinar, AnitaDe Visser, SamuelPineault, NicolasDe Vos, JohnPineda, MiguelDe Vries, CarliePiñeiro, MartaDe Zotti, MartaPinela, JoséDean, David A.Ping, Yueh-HsinDean, Matthew J.Pingault, LiseDeb, SubrataPingitore, PieroDebela, Ahmed MehdiPinho, Brígida RibeiroDebelius, JustinePinkas, AdiDeBosch, BrianPinske, ConstanzeDębowski, DawidPinto, ErnaniDebuchy, RobertPinto, SandraDechant, ReinhardPinzaru, IuliaDecker, RichardPiomboni, PaolaDeclercq, WimPiontek, JorgDedhar, ShoukatPiotrowska, AleksandraDeevska, GerganaPiotrowska, EwaDegenhardt, KarlPiovesana, SusyDegens, HansPiozzi, AntonellaDegn, MatildaPipan, BarbaraDegola, FrancescaPiperno, AnnaDeGregorio, DaniloPirazzini, MarcoDeineko, Elena VictorovnaPires, Bruno R. B.Dekker, MarloesPirisi-Creek, LuciaDel Duca, StefanoPirity, Melinda K.Del Fattore, AndreaPirman, TatjanaDel Fresno, CarlosPiro, GenyDel Giacco, LucaPiro, Rosario M.Del Papa, NicolettaPiróg, Katarzyna A.Del Prete, AnnalisaPirola, LucianoDel Real, GustavoPirona, RaulDel Río Celestino, MercedesPirtoli, LuigiDel Rio, DanielePisani, LucianaDelaissé, Jean-MariePisanu, ClaudiaDelbarre-Ladrat, ChristinePiscopo, MarinaDelbue, SerenaPisegna, Prof J.Delebarre, ChristophePisitkun, TrairakDéleris, PaulPisk, JanaDelevoye, CédricPisklak, Dariusz MaciejDelfín, Dawn A.Pislariu, CatalinaDelgado-Calle, JesusPissarek, MargitDelhanty, PatricPistocchi, Anna SilviaDelhomme, NicolasPiszczek, PiotrDelhon, GustavoPita, SebastianDeligeoroglou, EfthimiosPittalà, ValeriaDelihas, NicholasPitto, LetiziaDelisle, Jean-SébastienPitucha, MonikaDella Pepa, GiuseppePiu, SahaDella Ragione, FulvioPiva, TerrenceDella Torre, SaraPivarcsi, AndorDellambra, ElenaPiya, SarbottamDelle Monache, SimonaPizzo, Riccardo C.Delle Piane, MassimoPizzolanti, GiuseppeDellepiane, SergioPlacido, JerssonDellis, OlivierPlader, WojciechDello Ioio, RaffaelePlanas, MartaDell’Oste, ValentinaPlanchais, SéverineDelvin, Edgard E.Planchon, Sarah M.DeMarshall, CassandraPlastina, PierluigiDemartini, ChiaraPlatania, ChiaraDemerdash, Omar N.A.Platonov, PyotrDemir, FatihPlatt, Jeffrey LDemyanets, SvitlanaPlatta, HaraldDemyda-Peyrás, SebastiánPlaza-Zabala, AinhoaDen Hartigh, LauraPŁażek, AgnieszkaDenecke, ShanePlenis, AlinaDeng, NanjiePlessas, StavrosDeng, Wei-WeiPlettner, ErikaDeng, Win-PingPletzer, DanielDeng, XufangPlevin, RobinDeng, YoupingPlíhal, OndřejDenifl, StephanPlohl, MiroslavDenis, SerenoPloner, ChristianDenisow, BozenaPlonka, PrzemyslawDenizot, JéremyPlösch, TorstenDenko, NicholasPlotkin, Lilian I.Deplazes, EvelynePlotnikov, Egor Y.Depmski, RobertPluciennik, ElzbietaDePoy, Lauren M.Pluquet, OlivierDepping, ReinhardPluta, RyszardDerler, IsabellaPluth, JaniceDeryusheva, SvetlanaPlyusnin, Victor F.Desai, AbhishekPniewski, TomaszDesai, ChandniPobbati, AjaybabuDesai, JigarPocheć, EwaDesfougères, YannPochi, SubbarayanDeshmukh, RupeshPochini, LorenaDeshpande, ShayuPochynyuk, OlehDesiderio, AntonellaPodechard, NormandDesmet, TomPodlewska, SabinaDesprès, PhilippePoggi, AlessandroDessen, AndréaPoglitsch, MarkoDessolin, JeanPohjoismäki, Jaakko L. O.Deth, RichardPohl, EhmkeDettori, Maria LuisaPohl, PawelDevarajan, PriyadharshiniPohl, SebastianDevarshi, Prasad P.Póka, RóbertDevesa, JesúsPokorski, MieczyslawDevine, Patrick J.Poku, Rosemary A.Devireddy, AmithPolakowski, Nicholas J.Devy, JérômePolańczyk, AndrzejDey, Bijan KPolat, Emre OzanDey, GangotriPolen, TinoDey, MadhusudanPolesello, CédricDey, SoumyadeepPoleszczuk, JanDeyev, Igor E.Polewski, KrzysztofDhakal, DipeshPoli, GiulioDhakal, SomaPoli, MauraDhar, PrajnaparamitaPoli, ValeriaDhariwal, RamanPolicastro, LuciaDharker, NachiketPolimanti, RenatoDharmasiri, NihalPolina, IuliiaDhawan, DeepikaPolishchuk, PavelDhawan, GauravPoljak, IgorDhillon, Braham Deep SinghPollari, FrancescoDhiman, SaurabhPollier, JacobDi Bartolomeo, SabrinaPollok, Karen E.Di Blasio, AlbertoPolonyi, JanosDi Bonito, PaolaPoltronieri, PalmiroDi Bussolo, ValeriaPoluzzi, ElisabettaDi Costanzo, AntonellaPoma, AnnaDi Donato, MarziaPoma, PaolaDi Fazio, PietroPonnandy, PrabhuDi Filippo, Ester SaraPonomarev, EugeneDi Franco, ManuelaPons, JosefinaDi Franco, SimonePons, MiquelDi Gianfilippo, RiccardoPons, SebastianDi Giovanni, CarmenPons, ToniDi Girolamo, RoccoPontius, Frederick W.Di Giulio, MassimoPonz-Sarvise, MarianoDi Liegro, ItaliaPoolos, Nicholas P.Di Luccia, AldoPoon, IvanDi Mambro, RiccardoPoór, MiklósDi Marco, StefanoPoór, PéterDi Maro, AntimoPop, Tudor LucianDi Martino, CatelloPopa-Wagner, AurelDi Matteo, SabinaPope, ChadDi Mauro, MichelePopeijus, Herman E.Di Nardo, FabioPopoff, StevenDi Natale, ConcettaPopolan-Vaida, Denisia M.Di Paola, RosannaPopolo, AdaDi Pasqua, Laura GiuseppinaPopova, AntoanetaDi Pierro, ProsperoPopper, Helmut H.Di Pietro, CinziaPorceddu, AndreaDi Pietro, PaolaPordea, AncaDi Pompo, GemmaPorporato, PaoloDi Salvo, EleonoraPort, MatthiasDi Sansebastiano, Gian-PietroPort, Sarah A.Di Sotto, AntonellaPortela, MartaDi Stasio, DarioPorter-Stransky, KirstenDi Zazzo, ErikaPortillo, Maria Del CarmenDiack, Abigail B.Porzionato, AndreaDiakos, ConniePósa, AnikóDiamond, GillPosa, LucaDiana, JamesPoŝa, MihaljDiana, PatriziaPosada Arias, SilviaDias-Ferreira, JoãoPoschenrieder, CharlotteDIAZ RUBIO, OLGAPosey, Karen L.Díaz, EsperanzaPosselt, DortheDíaz-Hernández, MiguelPostila, Pekka A.Diaz-Rodriguez, PatriciaPostma, Alex V.DiCenzo, George CPoston, BrachDickstein, RebeccaPotaczek, Daniel PDiddens, DiddoPotaczek, Daniel P.Didion, Sean P.Poterszman, ArnaudDie, JosePothuraju, RameshDiederich, MarcPotokar, MajaDiekman, AlanPotopnyk, MykhayloDieni, CristinaPotrykus, KatarzynaDiepstra, ArjanPottosin, IgorDietrich, AlexanderPoudel, BarunDietrich, Johannes W.Poulin, RemingtonDiharce, JulienPouliot, FrédéricDijkstra, BaukePoulos, RebeccaDileep, VishnuPoulsom, RichardDiliën, HannePourquier, PhilippeDillman, Adler R.Pourroy, GenevieveDillon, Laura W.Poveda Larrosa, Jose AntonioDimarogona, MariaPovedano, Raúl EstévezDimitrellou, DimitraPoverennaya, Ekaterina V.Dimitroff, Charles J.Powers, Amanda K.Dimitrov, EugenePowrózek, TomaszDimmock, Jonathan R.Poyner, DavidDimova, ElitsaPozo, ÓscarDinescu, SorinaPozsgai, GaborDing, CherlynPozzi, CeciliaDing, JianxunPozzobon, MichelaDing, QingbaoPradet-Balade, BerengereDing, TianbingPradotto, Luca GuglielmoDing, ZhaoyangPrajzler, VaclavDini, LucianaPrakasam, SivaramanDini, PouyaPrakash, AishwaryaDinischiotu, AncaPrakash, DivyaDiniz, CarmenPranckeviciene, ErinijaDinulescu, Daniela M.Prasad, KasavajhalaDiomede, FrancescaPrasad, Kasavajhala V. S. K.Dion, Patrick A.Prashar, SanjivDionisio, GiuseppePrat, MariaDioum, El Hadji M.Prats, Anne-CatherineDiSpirito, AlanPrentice, HowardDissel, StephanePrescott, AlanDiStefano, JohannaPrestel, MatthiasDiStefano, Marina TPreziosa, PaoloDistelfeld, AssafPriante, GiovannaDiTacchio, LucianoPrice, JeffDitta, AllahPrice, Neil P. J.Dittmar, GunnarPrice, PatriciaDittmar, ThomasPrice, Richard B.Dittmer, Neal T.Prickett, TimDivino-Filho, JosèPricl, SabrinaDixit, RamPrieto, ManuelDjuric, ZoraPrimeaux, StefanyDkhar, Hedwin KitdorlangPrince, Silvas JDo, Sun HeePringault, OlivierDo, Yi-YinPrinsi, BhaktiDobens, Leonard L.Printza, NikoletaDoberentz, ElkePrisic, SladjanaDoblin, MonikaPritchard, MicheleDobosz, RobertPrivé, Gilbert G.Dobramysl, UlrichPrivette Vinnedge, LisaDobrikova, AneliaPriyamvada, LalitaDobrikova, Anelia G.Prochazka, MarekDobrindt, UlrichProchazka, RadekDobritsa, Anna A.Proctor, Carole J.Dobrowolska, GrażynaProfire, LenutaDobson, Tara H.W.Profumo, ElisabettaDocea, Anca OanaProietti, SilviaDocimo, TeresaProsperi, EnnioDocon, M PilarProsperi, Jenifer R.Doda, Sai ReddyProtheroe, AndrewDoddapattar, PrakashPrough, Donald S.Dodero, VeronicaProvot, SylvainDodig, DejanPruess, Birgit M.Doekes, GertPruneda-Paz, JoseDoi, KazuyaPrzekora, AgataDoig, CraigPrzepiera-Będzak, HannaDolan, StephenPrzyborowski, JerzyDoležal, KarelPrzybył, Jarosław L.Domenicali, MarcoPrzybyłek, MaciejDomenici, GiacomoPrzybyło, MałgorzataDomingo, EstebanPrzybylski, PiotrDomingo-Musibay, EvidioPsarra, Anna-Maria G.Domingues, SaraPshezhetsky, AlexeyDominique, DurandPszczółkowska, AgnieszkaDomoki, FerencPuca, AnnibaleDonaire, LiviaPucciarelli, SandraDonati, BenedettaPuccini, MonicaDonato, RosarioPuchalka, RadoslawDonato, Rosario FrancescoPuchart, VladimírDong, FengPuchkov, EvgenyDong, YiboPuchkova, Ludmila V.Dong, YuanchenPuertas, Maria CarmenDongmo, SaustinPuglia, Anna MariaDoni Jayavelu, NareshPuglia, GiuseppeDonizetti, AldoPugliesi, ClaudioDonnini, SandraPuhka, MaijaDonzelli, ElisabettaPuiggalí, JordiDonze-Reiner, TeresaPuiu, MihaelaDoppler, StefaniePuizina, JasnaDore, Maria PinaPujadas, GerardDores, Michael R.Pulavarti, Surya VSRKDormond, OlivierPulgar, VictorDorozhkin, Sergey V.Pulignani, SilviaD’Orsi, BeatricePullen, NicholasDosoky, NouraPuniya, Bhanwar LalDoss, Michael XavierPuppi, DarioDou, LaetitiaPurcell, Erin B.Dou, ZhichengPusch, StefanDouda, OndřejPüschel, Gerhard P.Doudin, KhalidPushchina, EvgeniyaDougherty, JosephPuthanveetil, PrasanthDouros, AntoniosPutilov, ArcadyDovat, SinisaPutla, SudarsanamDovizio, MelaniaPutnam, William C. (Trey)Downie, J. AllanPutz, MihaiDoyle, Lucinda ElizabethPutz, Mihai V.Dračínský, MartinPuzianowska-Kuźnicka, MonikaDragomir, MihneaPytel, PeterDragos, HorvathPytka, KarolinaDragutan, ValerianQasim, MuhammadDreier, RitaQasim, SaadDresler, SławomirQi, FengDriscoll, James JQi, TiancongDrlica, KarlQiao, PengfeiDrobek, MartinQin, SanboDrobizhev, MikhailQin, Xian-YangDrosten, MatthiasQin, XiaoqiongDrougard, AnneQin, ZhenpengDroździk, MarekQiu, BinDS, GalileoQiu, JiaenDu, GuodongQiu, XinDu, HaoQiu, ZhenDu, LiqunQu, NingDu, XingrongQuan, TaihaoDuan, JialeiQuarles, EllenDuarte, AmilcarQuartu, MarinaDuarte, Maria CristinaQuax, PaulDuarte, T. L.Queiroz, Maria JoãoDuatti, AdrianoQueralt, EthelDube, PrabhatchandraQueralt-Martín, MaríaDubey, NileshkumarQuesada Pérez, VíctorDubey, RichaQuide, YannDubois, AnnickQuintarelli, ConcettaDubrez, LaurenceQuinton, Lee J.Dubrova, YuriQuirós, Luis M.Dubrovina, Alexandra S.Quock, Raymond M.Dubrovskiy, Andrey A.Qureshi, TanvirDuch, CarstenRaasmaja, AtsoDuchi, SerenaRabanal-Ruiz, YoanaDuchowicz, PabloRabbani, GulamDuda, MałgorzataRabideau, BrooksDudakova, LubicaRace, PaulDudas, JozsefRachek, LyudmilaDuddy, WilliamRacz, IldikoDudhipala, NarendarRadaram, BhaskerDudnikov, Alexander Ju.Radenkovic, MiroslavDudok, JeroenRadin, Daniel P.Duerr, ClaudiaRadisavljevic, ZivDuffy, Aaron M.Radke, Jay RDufour, AntoineRadošević-Stašić, BiserkaDugé De Bernonville, ThomasRadtke, AleksandraDuggal, NishaRadu, MihaiD’Ugo, EmilioRadzymińska-Lenarcik, ElżbietaDuguez, StephanieRae, ColinDullaart, Robin P.F.Raffaniello, Robert D.Dullart, RobinRafiei, HosseinDuma, Virgil-FlorinRaftery, Rosanne M.Dumbrava, AncaRagauskas, ArthurDumee, LudovicRagavan, MukundanDumez, SylvainRaggi, LorenzoDumic, JerkaRagni, EnricoDumitrica, TraianRagnini-Wilson, AntonellaDumler, FrancisRagno, GaetanoDumond, HeleneRagno, RinoDuncan, Raymond ScottRagusa, AndreaDunlop, ElaineRagusa, Maria AntoniettaDunne, NicholasRahal, OmarDunning, ChelseaRahat, Michal A.Duong, Hai MinhRahimi, FaridDupin, DamienRahimi, HomairaDupureur, Cynthia M.Rahimi, ParvanehDurand, Béatrice C.Rahimi, ShadiDuranton, ChristopheRahman Pour, RahmanDurazzo, AlessandraRahman, M. AzizurDurek, ThomasRahman, MilladurDurlik, MagdalenaRahman, SafikurD’Ursi, Anna MariaRahmathulla, GazanfarD’Ursi, PasqualinaRai, NavneetDuryee, Michael J.Rai, PrakashDusart, IsabelleRai, VikrantDusny, ChristianRaikhy, GauravDusso, AdrianaRaimondi, LaviniaDuszczyk, JakubRaimondo, StefaniaDuszka, KalinaRaina, MedhaDutartre, PatrickRaina, SatishDuterque-Coquillaud, MartineRainger, JoeDutta, ArijitRaj, NitinDutta, PranabanandaRajagopal, SudarshanDutta, RahulRajagopalan, NandhakishoreDuun Rohde, PalleRajão, Daniela S.Duvigneau, J. CatharinaRajarapu, Swapna PriyaDvořáčková, MartinaRajarathnam, KrishnaDvorakova-Hortova, KaterinaRaje, MithunDwivedi, ChandraharRajendran, VazhaikkurichiDworatzek, ElkeRajić, ZrinkaDworkin, SebastianRajnarayanan, RajendramDyakin, Victor V.Rajpert-De Meyts, EwaDyall, JulieRajpurohit, SurendraDyer, NigelRak, JanuszDyguda-Kazimierowicz, EdytaRakhmatullin, AydarDykes, Iain M.Rakonczay, ZoltánDyshlovoy, Sergey A.Ralli, MassimoDysli, ChantalRama, Ana RosaDyson, Paul J.Ramachandran, SrinivasanDziegiel, PiotrRamachandran, VinoyDzięgiel, PiotrRamadan, QasemDziewit, LukaszRamadas, AmuthaDzikowska, AgnieszkaRamaiahgari, Sreenivasa C.Dzobo, KevinRamalingam, NagendranDzolic, ZoranRamalingam, NaveenEamens, Andrew L.Ramana Pidatala, VenkataEbeed, HebaRamani, KomalEbnet, KlausRamasamy, ElamparuthiEboigbodin, KevinRamasamy, MohankandhasamyEcelbarger, Carolyn A.Rambaud, CarolineEchevarria, MiriamRamchandani, DivyaEchigoya, YusukeRamesh, Sunita A.Ecke, ThorstenRamić, SnježanaEcker, GerhardRamírez, MartínEckschlager, TomasRamírez-Bergeron, DianaEdae, Erena A.Ramírez-Vélez, RobinsonEdamakanti, Chandrakanth RRamirez-Vick, JaimeEdderkaoui, MouadRamonell, KatrinaEder, Iris E.Ramoni, RobertoEderli, LuisaRamos, AdrianaEdge, RuthRamos, CarmenEdin, Nina JeppesenRamos, Roxana ValdésEdiriweera, Meran KeshawaRamos-Molina, BrunoEdström, DanielRampazzo, ElenaEdwards, Adrianne N.Rampioni, GiordanoEdwards, JoshuaRamsey, Ian ScottEfird, Jimmy T.Ran, SophiaEfremenko, ElenaRancourt, Rebecca C.Efremenkova, Olga VRandall-Demllo, SarronEftekhari, AliRandazzo, Giuseppe MarcoEfthimios, DardiotisRanf, StefanieEgami, HiromichiRangaswami, ArunEgawa, GyoheiRanieri, ElenaEgawa, NagayasuRanieri, GirolamoEgawa, TatsuroRanjan, AtulEgea Gutiérrez-Cortines, MarcosRanjan, KishuEgea, GustavoRanzato, EliaEgea, IsabelRao, ChristopherEgerszegi, IstvánRao, VidhyaEggel, AlexanderRaol, YogendraEgger, BorisRapacz, MarcinEgorova, KseniaRapak, AndrzejEhlen, J. ChristopherRapi, StefanoEhling-Schulz, MonikaRapp, Anna E.Ehnert, SabrinaRapposelli, SimonaEhrenberg, MansRascio, AgataEhrlich, HermannRashid, Mohammad HarunEhrnhoefer, DagmarRašin, Mladen RokoEid, NabilRasmussen, Angela L.Eide, DavidRasmussen, CarolynEijkelkamp, BartRasmussen, Soren K.Eikmans, MichaelRasoulianboroujeni, MortezaEileen, MoritzRaspanti, MarioEilertsen, Karl-ErikRassoulzadegan, MinooEisenberg, TobiasRastegar, MojganEiz-Vesper, BrittaRastegar, SepandEkberg, JennyRastogi, AnshuEl Andaloussi, AbdeljabarRatajewski, MarcinEl Bairi, KhalidRatet, PascalEl Kasmi, FaridRather, GulamEl Khalloufi, FatimaRather, Irfan AhmadEl Sabban, MarwanRathinasabapathy, AnandharajanEl Shamieh, SaidRathinavelu, AppuElango, JeevithanRato, Luís Pedro FerreiraEldehna, WagdyRau, IleanaElder, RhoderickRauch, Bernhard H.Eldridge, SandyRauch, JensEleftheriadis, TheodorosRaudino, AntonioEleftheriou, Eleftherios P.Raudsepp, TerjeEl-Esawi, MohamedRauk, ArviElfenbein, JohannaRauner, MartinaElferink, CornelisRausch, ManuelEl-Gazzar, Mohamed M.Rausch, VanessaEl-Gendy, Ahmed A.Raut, SangramElias, Martina S.Rautio, JarkkoEliseeva, IrinaRavaioli, StefanoEliseeva, TatianaRavan, MaryamEljaafari, AssiaRavanidis, StylianosElleder, DanielRavegnini, GloriaElli, MarinaRavelonandro, MichelElMallah, Mai K.Ravindranathan, SruthiElmann, AnatRavuri, SudheerElomaa, PaulaRaya, AngelEloy, Catarinarayan, anwarElsaadany, MostafaRayego-Mateos, SandraElsea, Sarah H.Rayner, BenElshabrawy, HatemRaz, VeredEl-Sheikh Ali, HossamRazaviyan, JavadElvas-Leitão, RubenRazzaque, Mohammed S.Elyaman, WassimRe, LambertoEmmersen, JeppeRead, DavidEndo, MakotoReagan, John LEndo, MitsuharuReal-Fernández, FelicianaEndoh, TamakiRebane, AleksEndresen, DagRébé, CédricEne, IulianaRebmann, VeraEng Poh, NgRebsam, AlexandraEngedal, NikolaiRebuffat, SylvieEngelberg, DavidReddy, Marpadga A.Engelberth, JurgenReddy, Pullagurala Venkata LaxmaEngelund, Morten B.Reddy, SakamuriEnguita, FranciscoReddy, Sudhir PuttyEnguita, Francisco J.Reddy, UmeshEnomoto, AtsushiRedegeld, FrankEnomoto, HirayukiRedwine, DavidEom, Gwang HyeonReed, May J.Eppler, ElisabethReeke, George N.Epstein, Alan L.Reers, StefanEraña, HasierReetz, Manfred T.Erban, TomasReeve, AmyErdélyi, MiklósReeve, VivienneErdmann, RalfReeves, MatthewErdő, FranciskaRegal, JeanErdődi, FerencRegalia, AnnaErdoes, GaborRegina, T.M.RErgün, SüleymanReginato, MauricioEric Kast, RichardRegmi, BishnuErickson, Michelle A.Regni, LucaEriguchi, MasahiroReguła, JulitaEritja, RamonRehfeld, JensErmakov, ArtemRehman, MichaelErmolaeva, SvetlanaReichert, David E.Ernst, ChristinaReimann, Regina R.Eroğlu, AbdulkerimRein, AlanErson-Omay, Emine ZeynepRein, TheoErzurumluoglu, A. MesutReinders, AnkeEsadze, AlexandreReinhardt, ChristophEscames, GermaineReis, HenningEscargueil, AlexandreReiser, Peter J.Escher, PascalReiter, Russel J.Escolà-Gil, Joan CarlesRejinold, SanojEscors, DavidRelat, JoanaEscott, CarlosRemesh, SoumyaEscudé, ChristopheRemondelli, PaoloEslani, MediRen, HongEspada, JesúsRen, QingzhongEspadas Villanueva, IsabelRen, YulinEspejo, JaimeRenaudineau, YvesEspel, EnricRenau-Morata, BegoñaEspen, LucaRenčiuk, DanielEspinach, Francesc XavierRengaraj, DeivendranEspinosa-Diez, CristinaRenimel, IsabelleEspinoza, Luis RRenukuntla, JwalaEsposito, Emilio XavierRepnik, UrskaEsposito, FrancaRequena, JesusEsposito, FrancescoRequena, Jose MaríaEsposito, SergioRequicha, JoaoEsque, JérémyRequicha, JoãoEsquifino, Ana IsabelRESCH, UlrikeEsse, RubenReščič, JurijEsser, CharlotteRescifina, AntonioEsser, Kjell B.Reshkin, Stephan JoelEssid, Sumia MohamedRestaino, Odile FrancescaEstanis, NavarroReszka, EdytaEsteban, Armando AriasRetamal-Salgado, JorgeEstelrich, JoanRetana-Marquez, SocorroEsteve-Romero, JosepRety, StephaneEstévez, JorgeReukov, VladimirEsworthy, SteveReuter, KlausEtich, JuliaReverter, EnricEtienne-Manneville, SandrineRevuelta, JuliaEttayapuram Ramaprasad, Azhagiya SingamRey, LuisEudald, CasalsRey, PascalEufemi, MargheritaReyes, ArchieEuler, GerhildReza Safaei, MohammadEva, AlessandraReznik, Jacqueline E.Éva, MikóReznik, SandraEvangelisti, EdouardReznikov, Leah R.Evans, Franklin D.Rezvani, KhosrowEvans, RogerRezzani, RitaEvenden, MayaRhazi, LarbiEvergren, EmmaRhee, HandooEverhart, JoshuaRho, MinaEvrard, Solène M.Riar, Amanjot KaurExbrayat, Jean-MarieRiazalhosseini, YasserExcoffon, KatherineRibaldone, Davide GiuseppeEyre, NicholasRibeiro, Rui S.Ezer, DaphneRibeiro, TiagoEzhov, MaratRibeiro, Viviana P.Ezura, YoichiRibeiro-Barros, Ana I.Fabris, LindaRicagno, StefanoFabris, LucaRicardo, SaraFacchiano, AngeloRicart, SusagnaFaccio, GretaRicca, EzioFacella, PaoloRiccardi, ClaudiaFackenthal, JamesRiccetti, LauraFadeeva, IrinaRicciardelli, CarmelaFadini, Gian PaoloRichard, Guy-FranckFagen, JennieRichard, PeterFahim, AhmedRichards, Dylan J.Fahnestock, MargaretRiches-Suman, KirstenFaijer, PeterRichner, JustinFailla, Cristina M.Richter, PeterFairclough, Robert H.Richtera, LukášFais, StefanoRideout, Elizabeth J.Faitot, FrancoisRider, CynthiaFajkus, JiříRidiandries, AnisyahFakhouri, Walid D.Rienzo, MonicaFakhrullin, Rawil F.Riffelmacher, ThomasFalasca, MarcoRigas, StamatisFalavigna, VitorRighetti, LauraFalcão, AndreRigó, GáborFalcieri, ElisabettaRigobello, MariapiaFalco, AlbertoRijo, PatríciaFalcón-Pérez, Juan ManuelRikkerink, ErikFalet, HervéRiley, RobertFalistocco, EgiziaRim, Kyung TaekFalls, ZackaryRimer, MendellFalsini, SaraRimoldi, SimonaFalzarano, DarrylRimondini, LiaFalzone, LucaRinaldo, SerenaFamilari, MaryRincon, Melvin Y.Famulski, JakubRincón, Sonia V. DelFan, ChenguangRinge, DagmarFan, Guo-changRink, LotharFan, Hueng-ChuenRios, FranciscoFan, MeiyunRisco, CristinaFan, QiongRishi, ArunFandrey, JoachimRising, AnnaFang, Evandro F.Riteau, NicolasFang, FrancisRitler, DominicFang, Hsu-WeiRitu, PandeyFang, JunnanRiuzzi, FrancescaFang, MarongRiva, FedericaFang, MinRivas, CarmenFang, MingxiRivas, FatimaFang, QingmingRivellese, FeliceFang, Ronnie H.Rivera, VictorFang, SungSoonRivera, Víctor M.Fang, XianjunRivero, FranciscoFang, XiaolanRivero-Müller, AdolfoFang, YiRivers, JasonFang, Yi-PingRivolta, CarloFanizzi, Francesco PaoloRizk, MartaFantini, JacquesRizos, HelenFantini, SebastianRizzi, LauraFarantos, Stavros C.Rizzi, MenicoFarcas, Anca CorinaRizzi, VitoFardeau, Marie-LaureRizzo, CarmenFare, SilviaRizzolio, FlavioFarè, SilviaRizzuti, BrunoFarge, GeraldineRizzuto, EmanueleFaria De Oliveira, MichelliRoberta, VenturellaFaria, Jorge M.S.Roberts, Arthur G.Farina, Nicholas H.Roberts, Drucilla J.Farkas, Attila E.Robertson, AvrilFarkas, MichaelRobin, Arif Hasan KhanFarkaš, PavolRobin, Jérôme D.Farnese, Fernanda S.Robinson, EdwardFárník, MichalRobinson, JamesFaroni, AlessandroRobinson, NirmalFarr, Christine J.Robles, PedroFarré Belmonte, MartaRoby, KatherineFarrell, HelenRoca-Sanjuán, DanielFarrugia, BrookeRocca, CarmineFasinu, PiusRoccella, MicheleFasoula, VasiliaRocha Ferreira, EridanFaustino, RandolphRocha, JavierFauzi, IlianaRocha, SoniaFavaloro, Emmanuel J.Rocha-Ferreira, EridanFavaretto, NiccolòRochani, AnkitFavi, EvaldoRocha-Rodrigues, SílviaFazarinc, GregorRoche, BenjaminFeas, XesusRoche, MarjolaineFeás, XesúsRockel, Jason SFebbraio, FerdinandoRockwood, DonaldFedele, AnthonyRoderick, LlewelynFedele, FrancescoRodilla, VerónicaFedele, MonicaRodolfo, CarloFederico, AntonioRodrigo, LuisFehér, AttilaRodrigues, António SebastiãoFehr, AnthonyRodrigues, Célia F.Fehse, BorisRodrigues, JoaoFelder-Schmittbuhl, Marie-PauleRodrigues-Pousada, Renato AlbertoFeldkamp, MarciaRodríguez Alvarez, LleretnyFeldman, DaleRodríguez Hermosa, Juan LuisFeldman, SteveRodriguez Peralvarez, ManuelFelgueiras, HelenaRodríguez, AmaiaFelgueiras, Helena P.Rodríguez, IsaacFeliciano, JoanaRodríguez, IsabelFeligioni, MarcoRodriguez-Contreras, AdrianFeliu Torres, NeusRodriguez-Cueto, CarmenFélix, LuisRodriguez-Garcia, MartaFelszeghy, SzabolcsRodríguez-González, ÁlvaroFeng, HailanRodríguez-Henche, NievesFeng, HuiRodríguez-López, PedroFeng, JingRodríguez-Lorenzo, LuisFeng, Sheng-WeiRodríguez-Lozano, Francisco JavierFeng, Wei-shengRodríguez-Morató, JoseFenoglio, IvanaRodriguez-Sinovas, AntonioFerens-Sieczkowska, MirosławaRodriguez-Vita, JuanFeresin, Rafaela G.Rodríguez-Yoldi, María JesúsFerguson, BradleyRoe, Daniel R.Ferguson, DavidRoedel, FranzFerji, KhalidRoelen, BernardFerlazzo, NadiaRoelink, HenkFernandes, PedroRoelofs, JeroenFernandez, Agustin F.Rog, JoannaFernandez, AlbertoRogakou, EmmyFernández, IgnacioRoger, JérômeFernández, José A.Rogers, HilaryFernandez, Marina O.Rogers, ThomasFernández, MarioRogobete, Alexandru FlorinFernandez-Abascal, JesusRogov, VladimirFernández-Álvarez, AlfonsoRogozhin, EugeneFernandez-Botran, RafaelRoh, SanghoFernández-García, MartaRohacs, TiborFernández-Mateos, PilarRohayem, JuliaFernando Teruhiko, HataRojano-Delgado, Antonia MariaFernando, M. RohanRojas-Tapias, Daniel FernandoFerracini, RiccardoRojo, LuisFerrando, LauraRojo, Maria AngelesFerrante, AntonioRolfo, AlessandroFerrante, ClaudioRolfo, ChristianFerrante, PatriziaRolhion, NathalieFerrara, NicolaRolla, GiovanniFerrarese, CarloRolland, AntoineFerrari, LucRom, SlavaFerrari, Paolaroman, koernerFerrario, ValerioRomanenko, Svetlana A.Ferrarotti, FrancescoRomani, AndreaFerraz, Emanuela PradoRomani, MassimoFerree, PatrickRomani, PatriziaFerreira, Jose M.F.Roman-Junior, WalterFerreira, LeonardoRomano, GabrieleFerreira, Luciana B.Romano, JuliaFerreira, RitaRomano, M CarmenFerreira, Tracie L.Romano, NiclaFerreiro, Anxo FernándezRomano-Chernac, Fabian B.Ferreon, Allan Chris M.Romanov, VictorFerreon, JosephineRoma-Rodrigues, CatarinaFerrera, RenéRomeo, GiovannaFerreri, CarlaRomeo, MargaritaFerrer-Miralles, NeusRomeo, RobertoFerrero, SimoneRomero, AlejandroFerretti, ElisabettaRomero, CarmenFerri, FlaminiaRomero, Fernando M.Ferri, Giovanni MariaRomero-Muñiz, CarlosFerrigno, AndreaRomo, Maria Del Mar Ortega-villaizanFerrini, SilvanoRonca, AlfredoFerro, RiccardoRoncada, PaolaFerroni, PatriziaRonchetti, SimonaFesenko, IgorRondanino, ChristineFestuccia, NicolaRong, ChaoFett-Neto, ArthurRonga, DomenicoFiacco, ToddRongvaux, AnthonyFiandra, LuisaRonsard, LaranceFiaschi, TaniaRoot-Bernstein, RobertField, MichaelRoque, AliciaField, RobertRosa, Bruce A.Field, Seth J.Rosa, CristinaFiering, Steven N.Rosa, DivellaFierro, José FernandoRosa, ViniciusFigiel, AdamRosado, CarlosFigueiredo, AndreiaRosales-Mayor, EdmundoFigueiredo, DuarteRosato, AntonioFigueiredo, Marxa L.Rose, AdamFigueroa-Romero, ClaudiaRoselli, MariannaFilannino, PasqualeRosellini, DanieleFilek, MariaRosendahl, JonasFilella, XavierRosenfeld, WarrenFilgueira, CarlyRosenholm, JessicaFiligheddu, NicolettaRosenthal, KenFilipe, HugoRosenthal, RitaFilipeanu, CatalinRosenzweig, DerekFilipecki, JacekRoseti, LiviaFilipova, IneseRosilio, VéroniqueFilippi, FrancescaRoss, Ian LFilippov, AndreiRos-Santaella, Jose LuisFillmore, HelenRos-Santaella, José LuisFimognari, CarmelaRosselló, Josep A.Finco, IsabellaRossi, FrancaFine, James BurkeRossi, LorenzoFinegan, Katherine G.Rossi, LuisaFinelli, CarmineRossi, RiccardoFinetti-Sialer, Mariella MatildeRossi, VittorioFini, Mehdi ARossner, PavelFinkielstein, CarlaRostoker, GuyFinsterer, JosefRotblat, BarakFiocchetti, MarcoRoterman, IrenaFionda, CinziaRoth, MichaelFioramonti, XavierRotherham, MichaelFioravanti, A.Rotondi, MarioFiorentini, DianaRotrekl, VladimírFiorentino, GabriellaRottembourg, JacquesFiorillo, LucaRoubelakis-Angelakis, Kalliopi A.Fiorillo, MarcoRouet, RomainFirczuk, MalgorzataRouhana, LabibFirestein, BonnieRouillé, YvesFirneisz, Ga´borRouleau, EtienneFischer, Michael B.Roulia, MariaFischer-Fodor, EvaRousseau, FrançoisFiscon, GiuliaRousseau, MartheFisher, Jonathan S.Rousselle, PatriciaFishman, PninaRousset, RaphaëlFisk, John D.Rouyer, FrançoisFisslthaler, BeateRovatsos, MichailFiszer, AgnieszkaRoversi, PietroFitz, Nicholas F.Rovida, CostanzaFlak, Jonathan NRovida, ElisabettaFlamigni, FlavioRovnak, JoelFlask, Chris A.Rowbottom, AnthonyFleig, AndreaRowe, Dominic BrockFleischman, Angela G.Rowland, AndrewFleischmann, AchimRowley, ChristopherFleites, Laura A.Rowther, Farjana B.Fleming, Sherry D.Roy Choudhury, Dr. AryadeepFletcher, John S.Roy Choudhury, SwarupFletcher, Nicola F.Roy, DipankarFleury, FabriceRoy-Engel, AstridFlorea, Ana-MariaRoyle, Nicola J.Florens, NansRóżalska, BarbaraFlores, Francisco B.Rozhon, WilfriedFlores, IdhalizRozov, Serge M.Floresta, GiuseppeRozsival, PavelFlorio, TullioRozwadowski, TomaszFluri, FelixRóżycki, BartoszFocaroli, StefanoRu, SushanFodor, CsabaRua, DiegoFoecking, MelanieRubattu, SperanzaFogacci, FedericaRubina, KseniyaFogarty, GeraldRubini, MicheleFogolari, FedericoRubio, MyriamFoijer, FlorisRübsam, MatthiasFojtová, MiloslavaRubtsov, NikolayFolini, MarcoRucinski, MarcinFölster-Holst, ReginaRucker, RobertFonseca, AnaRudd, SeanFonseca, FilomenaRuddock, Lloyd W.Fontana, LuisRudnóy, SzabolcsFontàs, ClaudiaRudolf, EmilFonte, PedroRudolf, RuedigerFontes, Paulo ARudolph, ChristianForcales, Sonia-V.Rudrabhatla, SairamForero-Castro, MaribelRudzińska, MagdalenaForghieri, FabioRuegg, UrsForini, Francesca S.Ruelland, EricFormanowicz, DorotaRuess, Dietrich A.Formenti, DamianoRufino-Palomares, Eva E.Fornaguera, CristinaRuggieri, ValentinoFornai, FrancescoRuggieri, VitalbaFornai, MatteoRuggiero, CarmelindaFornaini, CarloRugnone, MatíasForner, LeopoldoRugonyi, SandraForrer, DanielRuíz De Porras, VicençForrester, StevenRuiz Pérez, María VictoriaFörster, CharlotteRuiz Téllez, TrinidadForster, JamesonRuiz, MatthieuForsyth, NicholasRuiz-Echevarria, Maria J.Forte, ElviraRumyantseva, NatalyaForte, MaurizioRungis Ruņģis, DainisFortuna, SaraRunte, ChristophFortunato, ElizabethRuonala, Mika O.Foster, TimRuoppolo, MargheritaFosu-Nyarko, JohnRupp, SteffenFotopoulos, VasileiosRupprecht, RainerFotouhi, OmidRurek, MichalFountain, Samuel J.Rusciano, Maria RosariaFournier, AntoineRussi, SabinoFournier, Neil M.Russo, AnnapinaFourtounas, CostasRusso, AntonioFourtounas, KonstantinosRusso, Gian LuigiFowkes, RobRusso, NinoFowler, MarcieRusso, SuzanneFox, GlenRustum, Youcef M.Foyez, Mahmud K ARuthstein, SharonFracanzan, AnnaRyabchikova, Elena I.Fradin, ChantalRyabov, EugeneFraga-García, PaulaRyan, Aimee KFragliasso, ValentinaRyan, Lisa K.Frampton, JohnRybak, Leonard P.Francesca, LunardiRychkov, DenisFrancesco, SmedileRychkov, GrigoriFrancesco, StellatoRychtáriková, RenataFrancés-Soriano, LauraRydz, JoannaFrancipane, Maria GiovannaRyo, UshiodaFrancis, Ashwanth ChristopherRysz, JacekFrancis, HeatherRyszawy, DamianFrancisco, VeraRytelewski, MateuszFranco, LuisRyu, Jae-HaFrancuz, TomaszRząsa, AnnaFrank, SašaRzepecki, RyszardFranke, JakobRzymowska, JolantaFranken, Nicolaas A.P.Rzymski, PiotrFranklin, Michael J.S. Gandhi, NehaFransen, MarcS. Oh, DanielFrański, RafałSá, RosáliaFranssen, Maurice C.R.Saad, Nizar Y.Franz, SandraSaakes, MichelFranzè, AnnamariaSaba, Nakhle S.Franzini, AncaSabater, BartolomeFrascaroli, GiadaSabatino, LauraFrau, JuanSabella, ErikaFrazer, J. KimbleSabol, MajaFrazzi, RaffaeleSaburi, WataruFreedman, JonathanSaccardo, PaoloFreeman, Jennifer L.Saccenti, EdoardoFreestone, PrimroseSacchetti, AlessandroFreire, Juan J.Saccone, ValentinaFreisinger, EvaSaczonek, Agnieszka OwczarczykFreitas, Ana CristinaSadasivam, MohanrajFreitas, RenataSadhukhan, AyanFrejd, FredrikSadiku, PranveraFrentiu, FrancescaSaeed, AramFreundt, Eric C.Safa, Ahmad R.Fridmanis, DāvidsSafavi-Rizi, VajihehFrielinghaus, HenrichSah, NirnathFrielingsdorf, StefanSaha, AchintoFrietze, SethSaha, MrinmoyFriis-Hansen, LennartSahab, SareenaFriml, JiriSaheera, SherinFrischauf, IreneSahoo, PabitraFrkanec, RužaSahoo, Pabitra KumarFroeyen, MatheusSahoo, SubhransuFroeyen, MathySahu, JagajjitFröhlich, EleonoreSahu, PranavFrolov, AndrejSahu, Ravi PFroriep, Ulrich P.Sai, KiranFrosini, MariaSaid, NeveenFrostegard, JohanSaielli, GiacomoFrugis, GiovannaSaika, HiroakiFrump, AndreaSaini, ShikhaFrydrychova, Radmila CapkovaSainlos, MatthieuFu, FuyouSaint-Dizier, MarieFu, LianwuSaintigny, PierreFu, Shih-FengSaint-Pol, JulienFu, XueyanSaisho, YoshifumiFu, YingSaito, KosukeFu, ZhongjieSaito, MarikoFučíková, KarolinaSaito, YoshiroFuentealba, ClaudiaSaito, YukihiroFuhrmann, HerbertSaitoh, KenjiFujihara, YoshitakaSaitoh, MasaoFujii, HiroakiSajic, MarijaFujii, RitsukoSakabe, MasahideFujimori, KoSakaguchi, MasakiyoFujioka-Kobayashi, MasakoSakai, AtsushiFujita, MasakiSakai, HidekiFujita, MasayukiSakai, HiromichiFujita, MitsuguSakai, HiroyasuFujita, NaokoSakai, RyuichiFujita, SayakaSakakibara, IoriFujita, YuSakamoto, KensakuFujita, YukiSakamoto, ShingoFujiwara, SatoruSakamuri, Siva Sankara Vara PrasadFukada, So-ichiroSakasai-Sakai, AkikoFukuchi, SatoshiSakata, YoichiFukuda, AkariSakr, SoulaimanFukuda, TakeshiSakuma, KunihiroFukudome, AkihitoSala, Lucia LaFukuhara, YasuyukiSala, RobertoFukui, HirokazuSala, ValentinaFukumitsu, NobuyoshiSalameh, ThereseFukunaga, KenjiSalanta, LianaFukuoka, ShuichiSalanţă, Liana ClaudiaFukushima, AtsushiSalas-Huetos, AlbertFukushima, HiroshiSałasińska, KamilaFukushima, TsuyoshiSalazar, Juan J.Fuller, BryanSaleem, MuhammadFumagalli, FrancescaSaleh, Fatima A.Fumagalli, LauraSaleh, TawfikFunel, NiccolaSalehzadeh-Yazdi, AliFunk, RichardSalem, AbdelhakimFunk, RyanSalemi, MicheleFuoco, AlessioSaler, MarcoFurtula, BorisSalerno, LoredanaFurue, MasutakaSalgado, Francisco JFurukawa, Jun-ichiSalgado-Somoza, AntonioFuruse, YukiSalgar, ShashikumarFuruya, ToshikiSalhab, HassanFusaki, NoemiSalifoglou, AthanasiosFusaro, MariaSalinas Rodríguez, BeatrizFusco, AlfredoSalinska, ElzbietaFusco, NicolaSalis, AndreaFusco, VittorioSalman, MootazFushida, SachioSalmina, AllaFutagami, TaikiSalopek-Sondi, BrankaFutrega, KathrynSalt, IanGabazza, Esteban C.Saltarella, IlariaGaber, TimoSalto, RafaelGábor, ParagiSaluja, Ashok K.Gabriel, RobertSalvadori, NicolaGadaleta, AgataSalvatore, GiulianaGaddam, Ravinder ReddySalvi, MauroGadella, Bart M.Salvi, SilviaGadikota, GreeshmaSalzer, IsabellaGaetano, CarloSalzet, MichelGafencu, AncaSamaja, MicheleGagaoua, MohammedSamal, SnehaGagat, MaciejSamavati, LobeliaGagliano, TeresaŠamec, DunjaGagliardi, Paolo ArmandoSamhan-Arias, AlejandroGahete, ManuelSamoylova, Tatiana I.Gahlaut, VijaySampaolesi, MaurilioGai, ChiaraSampath, KarunaGai, ZhiboSampathi, ShilpaGaipl, Udo S.Sampurna, ChatterjeeGajc-Wolska, JaninaSamulski, Richard JudeGajdács, MárióSanak, MarekGala, Rikhav PSanche, LeonGalasso, GiovanniSanches, SandraGalat, AndrzejSánchez Ballesta, María TeresaGalbiati, MassimoSánchez Crespo, MarianoGalderisi, UmbertoSanchez, ElenaGaldiero, Maria RosariaSanchez, GoarGaleazzi, RobertaSánchez, GoarGalelli, LucaSanchez, SabrinaGaleone, AldoSánchez-Ávila, Ronald MauricioGalgani, MarioSánchez-García, DavidGáliková, MartinaSanchez-Infantes, DavidGalindo, AntonioSanchez-Martinez, MelchorGalindo-Villegas, JorgeSánchez-Pérez, Ana MaríaGalizi, RobertoSancho, JaimeGalkin, MaximSancho, MatiasGall Troselj, KoraljkaSancho-Vaello, EneaGallardo-Guerrero, KarineSander, VeronikaGallavardin, ThibaultSandmann, GerhardGallegos, Martha PatriciaSándor, ZoltánGallemí, MarçalSandovici, IonelGallipoli, PaoloSandra, Lopez-LeonGallo, CarmelaSands, Jeff MGalochkina, Tatiana V.Saneyasu, TakaokiGalstyan, AnzhelaSan-Félix, Ana RosaGaluska, Sebastian P.Sanfilippo, CristinaGalvagni, FedericoSanganyado, EdmondGalvan, CristinaSanguedolce, FrancescaGalvão, Adelino M.Sankar, UmaGalvao, IzabelaSankaranarayanan, Nehru VijiGalvez Llompart, MariaSankarasubramanian, VishwanathGalzitskaya, Oxana V.Sanny, CharlesGama-Carvalho, MargaridaSans, AlbertGambardella, AlessandroSansone, AnnaGambari, LauraSantacroce, LuigiGamberi, TaniaSantamaria, EstrellaGambineri, AlessandraSantamaria, RitaGambino, GiorgioSanter, Frédéric RGamez, AlejandraSanthoshkumar, PutturGamez-Perez, JoseSanti, Chiara DeGammoh, NoorSanti, LucaGanapathy-Kanniappan, ShanmugasundaramSantiago, GilbertoGanau, MarioSantibañez, Juan F.Ganazzoli, FabioSantini, AntonelloGanci, FedericaSantini, EmanuelaGandhi, Maher K.Santino, AngeloGanea, DoinaSantofimia-Castaño, PatriciaGanetzky, RebeccaSantolla, Maria FrancescaGanewatta, MitraSantos, Ana PaulaGangaraju, RajashekharSantos, CarlaGangloff, Sophiesantos, JanineGanguli-Indra, GitaliSantos, JerranGanguly, AvishekSantos, SusanaGanguly, Diep R.Santos, Teresa AlmeidaGangur, VenugopalSantos-Coquillat, AnaGanji, RakeshSantos-Juanes, JorgeGannett, Peter M.Santucci, DanielaGantenbein, BenjaminSantulli, GaetanoGantz, StephanieSanyal, SumanaGao, BeiSanz, AnaGao, Chun-HongSanz-Clemente, AntonioGao, JieSanz-Saez, AlvaroGao, LongSaotome, MasaoGao, MinSapi, EvaGao, PeiyuanSapir, YuvalGao, XuSapudom, JiranuwatGao, XuefeiSarapultsev, AlexeyGao, XuewenSarasija, ShaarikaGaponenko, VadimSaravanan, Prathab BalajiGaraj, VladimírSarbini, ShahrulGaranto, AlejandroSardeli, ChrysanthiGaranto, AlexSardu, CelestinoGarau, GianpieroSaretzki, GabrieleGarbar, ChristianSargueil, BrunoGarbers, ChristophSarkar, AmritaGarcia Amorós, JaumeSarkar, DhrubaGarcia De Frutos, PabloSarkar, Mrinal K.García Souto, DanielSarkar, SibajiGarcia, AbelSarkarai Nadar, VenkadeshGarcía, ConcepciónSarli, Valeria DiGarcia, Coralia V.Sarnataro, DanielaGarcía, GabrielaSarnowski, TomaszGarcia, IdoiaSarti, Alba ClaraGarcía, María JoséSartore, LucianaGarcía, MontserratSarubbo, FiorellaGarcia, SoniaSarver, JessicaGarcia, VictorSasai, YasushiGarcia-Bailo, BibianaSasaki, HirokiGarcia-Calderon, MargaritaSasaki, HitoshiGarcía-Conesa, María-TeresaSasase, TomohikoGarcia-Estañ, JoaquinSase, AjinkyaGarcía-Estañ, JoaquínSass, HenrikGarcia-Estañ, Luis PerezSassi, YassineGarcía-Gasca, Silvia AlejandraSasso, Ferdinando CarloGarcía-Lara, JorgeSastre-Serra, JorgeGarcía-López, Joan R.Satani, NikunjGarcía-Maroto, FedericoSatbhai, SantoshGarcía-Martínez, SantiagoŠatínský, DaliborGarcía-Mauriño, SofíaSatitsuksanoa, PattrapornGarcia-Pardo, JavierSato, EiichiGarcia-Robles, InmaculadaSato, FuyukiGarcia-Ruiz, HernanSato, KeisakuGarcia-Sosa, AlfonsoSato, Marcello OtakeGarcia-Vallve, SantiagoSato, NorihiroGarcia-Verdugo, IgnacioSato, TatsuoGarcía-Villalón, Angel LuisSato-Bigbee, CarmenGarcin, ElsaSatoh, ShigeruGard, PaulSatou, RyousukeGarg, Abhishek D.Satou, TadaakiGarg, KawishŠatović, EvaGarinis, GeorgeSattley, W. MatthewGarofalo, MariangelaSatué, KatiuskaGarrido-Cárdenas, José AntonioSatuito, Cyril Glenn P.Garriga, PereSaul, DominikGarrity, DeborahSaurav, KumarGarrosa, ManuelSautto, Giuseppe A.Garrote-Adrados, José AntonioSavary, StéphaneGarten, RyanSavchenko, Tatyana V.Gartia, Manas RanjanSavenkov, EugeneGarvey, ChristopherSavoi, StefaniaGarziera, MaricaSavoie, Jean-MichelGas, PiotrSawai, HirofumiGasbarri, CarlaSawicki, JakubGase, KlausSawynok, JanaGasic, KsenijaSayanova, OlgaGaspar, Maria ManuelaSayans, Mario PerezGáspár, RóbertSaydam, OkayGaspar, VítorSberveglieri, VeronicaGáspári, ZoltánScafoglio, ClaudioGasparri, FabioScalschi, LoredanaGasparyan, Armen YuriScanu, Antonio MarioGasperini, SerenaScarafoni, AlessioGasque, PhilippeScarano, AntonioGasset, MariaScarborough, Robert J.Gastaldello, StefanoScarfì, SoniaGately, KathyScarlett, GarryGatenby, Robert A.Scarnati, EugenioGates, ColinScarpa, Grazia MariaGatesy, John E.Scarpellini, EmidioGatti, AntoniettaScatena, RobertoGatzka, MartinaScavello, FrancescoGaude, ThierryScavo, Maria PrincipiaGauden, Piotr A.Schaefer, BenediktGaudieri, SilvanaSchäfer, KatrinGaudin, RaphaëlSchäffner, AntonGauer, StefanSchaffner, DeniseGaumann, AndreasSchaiquevich, PaulaGaustad, Ann HelenSchank, Jesse R.Gauster, MartinScheben, ArminGautam, PrsonSchechter, Alan N.Gauthier, BenoitScheel-Krüger, JørgenGautier, MathieuScheiner, SteveGavazzo, PaolaScheller, Frieder W.Gavira, JoséSchemmer, PeterGawarecka, KatarzynaSchempp, Christoph M.Gawel, DamianSchenone, SilviaGawlik, AnetaSchepisi, GiuseppeGay-Quéheillard, JeromeScherer, GüntherGazerani, ParisaScherf, KatharinaGazzano, MassimoSchernthaner, JohannGazzetti, KatiaScherthan, HarryGdula-Argasinska, JoannaSchetz, Miet R.C.Ge, BaoxueSchiaffino, StefanoGe, WenzhenSchiavone, MarcoGe, XiaodongSchiavone, StefaniaGe, XiaoweiSchibler, UeliGeary, SeanSchildgen, OlivierGebhardt, ChristofSchild-Poulter, CarolineGechev, TsankoSchilling, SusanneGeddes, James WSchimmel, Bernardus C.J.Gee, WilliamSchindl, RainerGeerlof, ArieSchininà, Maria EugeniaGeetha, ThangiahSchinke, ThorstenGeeves, MichaelSchiraldi, ChiaraGeha, SamehSchirhagl, RomanaGehlert, SebastianSchlachetzki, JohannesGehring, ChristophSchlaepfer, DavidGelse, KoljaSchlaepfer, IsabelGendek-Kubiak, HannaSchlaepfer, Isabel RGenders, AmandaSchlathölter, ThomasGeng, FengSchleiffer, AlexanderGenova, TullioSchlothauer, TilmanGentile, PietroSchlüter, Klaus-DieterGentile, VittorioSchmetz, QuentinGentilucci, LucaSchmidt, EdGeorg, JensSchmidt, MartinaGeorg, RaphaelaSchmidtke, GunterGeorgakilas, AlexandrosSchmitt, AnthonyGeorgantzinos, Stelios K.Schmitt, JoachimGeorge, SarahSchmitz, BorisGeorgescu, AdrianaSchmitz, ThomasGeorgiadi, AnastasiaSchmitz-Linneweber, ChristianGeorgiev, Georgi As.Schmitz-Rixen, ThomasGeorgiev, VasilSchmoll, DieterGeppetti, PierangeloSchneckenburger, HerbertGeraci, FabianaSchneider, AnjaGeraghty, DanielSchneider, BarbaraGeraghty, PatrickSchneider, RenéGeraldes, PedroSchneider, SabineGerasimcik, NatalijaSchnichels, SvenGerbino, AndreaSchoenhagen, PaulGerdol, MarcoSchoepp-Cothenet, BarbaraGereñu, GorkaSchohn, HervéGergs, UlrichScholkmann, FelixGerhauser, IngoSchönitzer, VeronikaGerin, DonatoSchott, UlfGerke, JörgSchrader, AndreaGermain, HugoSchreiber, MartinGerman, Nadezhda A.Schrier, RachelGerogiorgis, DimitriosSchrock, MorganGeronès, Ferran FeixasSchröder, AgnesGerothanassis, Ioannis P.Schröder, CarolaGerriets, ValerieSchröder, ChristianGershon, TimothySchröder, Henrik DaaGescher, AndreasSchroeder, GrzegorzGesell, AndreasSchrum, AdamGessner, GuidoSchubert, DavidGestwicki, JasonSchubert, MarioGeszke-Moritz, MałgorzataSchuchardt, MirjamGetahun, AndrewSchueler, JuliaGetz, GodfreySchulte, ChristianGewirtz, David A.Schultz, CarolynGeyer, JoachimSchultz, David JGhaleh, BijanSchulz, Benjamin L.Ghandhi, ShanazSchulz, FlorianGhaste, ManojSchulz, MargotGhayor, ChafikSchulz, Rüdiger W.Ghelli, Anna MariaSchulze-Tanzil, GundulaGherasim, CarmenSchulze-Tanzil, Gundula GesineGhiggeri, Gian MarcoSchumann, CananGhigliotti, LauraSchutte, StaceyGhigo, AlessandraSchwab, RebeccaGhigo, EricSchwaminger, SebastianGHILA, LuizaSchwan, WilliamGhinassi, BarbaraSchwartz, GregoryGhinet, AlinaSchwarz, Steven M.Ghirlanda, GiovannaSchweighofer, AloisGhiroldi, AndreaSchweigkofler, WolfgangGhislat, GhitaSchweikert, LorianGhobadi, SajjadSchweizer, MichaelGhodake, GajananSchwerdt, JulianGhose, KaushikSchwerk, ChristianGhosh, AbhijitScicchitano, PietroGhosh, ArnabSciubba, FabioGhosh, RajeshwaryScognamiglio, MonicaGhosh, SantoshScorziello, AntonellaGhoshal, KalpanaScott, DanielGiacomazza, DanielaScotti, MarcusGiacomelli, ChiaraScotto D’Abusco, AnnaGiacomello, EmilianaScrima, MarioGiacomello, StefaniaScumaci, DomenicaGiacosa, SimoneScuruchi, MicheleGiagulli, CinziaSeale, Lucia A.Giampieri, FrancescaSeano, GiorgioGiampietro, LetiziaSebastian, AimyGianazza, ElisabettaSebastián, DavidGianesello, LisaSebastiana, MónicaGianfelici, ValentinaSebastianelli, LucaGianinetti, AlbertoSebastiani, FedericoGiannakas, ArisSebastiani, GuidoGiannakouros, ThomasSébastien, SartGiannoni, PatriziaSeca, Ana Maria Loureiro DaGiansanti, FrancescoSechi, GianPietroGiavaresi, GianlucaSeco-Rovira, VicenteGibbs, BernhardSedic, MirelaGibbs, Bernhard F.Sedlacek, AbigailGibert, YannSedlačík, MichalGibson, Susan I.Sedlák, ErikGiezenaar, CarolineSee Hoe, Louise E.Gigante, AntoniettaSeedorf, UdoGikas, EvagelosSeeger, HaraldGil Fernández, VanessaSeene, TeetGil, KrzysztofSegarra-Marti, JavierGil, MinchanSegatto, MarcoGiles, CoreySeger, RonyGiles, WayneSegers, VincentGill, Martin RSegura, AnaGillet, GermainSehgal, LalitGillevet, PatrickSehrawat, ArchanaGillies, RobertSeibert, Franz JosefGilmer, JohnSeidl, MaximilianGilmour, Matthew IanSeiffert, MartinaGilroy, Eleanor M.Seiler, AlexanderGimenez, EstelaSeiler, Magdalene J.Giménez-Gómez, PabloSeipel, KatjaGinex, TizianaSeligmann, HerveGiordano, AntonioSellars, JonathanGiordano, MarcoSelleri, SilviaGiordano, RaffaeleSelvam, ChelliahGiovannelli, PiaSemenyuk, PavelGiovanni, BUSCOSemisalova, AnnaGiovannini, AnnalisaSemreen, Mohammad H.Giovannoni, RobertoSena, ArmandoGiovarelli, MirellaSenbonmatsu, TakaakiGiraldo, PilarSenderowitz, HanochGirao, HenriqueSengupta, ArjunGirardis, MassimoSengupta, SadhakGirault, AlbanSeo, DaisukeGirijala, Raghavendra LSeo, Dong HoGirolami, MarcoSeo, Goo-YoungGirotti, Albert W.Seo, HeewonGismondi, AngeloSeo, ShigemiGitto, StefanoSeo, Young-KwonGiuca, Maria RitaSeoane-Collazo, PatriciaGiudice, ChiaraSepe, ValentinaGiuffré, AlessandroSerba, DesalegnGiulia, FioravantiSerefko, AnnaGiulia, MauriziSereikaite, JolantaGiuliani, MaurizioSergaki, MaritinaGiulietti, MatteoSergeant, KjellGiulotto, ElenaSergi, ConsolatoGiurg, MirosławSerif, ManuelGiussani, PaolaSerino, GiovannaGiusti, IlariaSerio, GabriellaGizdavic-Nikolaidis, MarijaSerra, StefanoGkaliagkousi, EugeniaSerralheiro, PedroGkolfakis, ParaskevasSerrano, IreneGkretsi, VasilikiSerrano-Aroca, ÁngelGłąbska, DominikaSerre-Beinier, VéroniqueGladish, Daniel K.Seruggia, DavideGladysheva, Inna P.Seth, Punit P.Glasa, MiroslavSethi, GautamGlaser, KeithSethi, SunjayGlaser, ShannonSeuberlich, TorstenGlassop, DonnaSeufferlein, ThomasGlazińska, PaulinaŠevčíková, TerezaGlendining, KellySevcsik, EvaGlimm, TilmannSeverino, BeatriceGliozzi, MicaelaSeverino, PaoloGlobisch, DanielSeverson, Aaron F.Gloria, AntonioSevilimedu Veeravalli, SathyanarayananGlotov, Andrey S.Sevilla, MichaelGłowacka, KatarzynaSevilla-Morán, BeatrizGluck, JessicaSewald, KatherinaGlukhova, Olga E.Seydel, KarlGnanaprakasam, JayaSgarbossa, PaoloGnanasundram, Sivakumar VadivelSgodda, MalteGnanvossou, DesireSgrignani, JacopoGniazdowska, AgnieszkaSH, TanGniewosz, MałgorzataShah, AjitGoda, KeisukeShah, RuchiGoddard, Julie M.Shahi, ShaileshGodfrey, Henry P.Shahjamali, MohammadGodlewska, MarlenaShahzad, BabarGodos, JustynaShakhnovich, ValentinaGoedegebuure, PeterShakya, AkhileshGoel, Peeyush N.Shamay, YosefGoffart, SteffiShamonin, MikhailGoh, Boon ChongShams, KaveGohda, EiichiShams, S. FatemehGok, OzgulSHANG, Jin EricGolanov, Eugene V.Shankar, EswarGoldenkova-Pavlova, IrinaShankar, RohitGoldfarb, David S.Shao, WeiGoldman, Maria HelenaShapiro, BruceGoldoni, MatteoSharan, RitiGoldschmidt, Eliezer E.Sharif, JafarGoldshmit, YonaSharif-Naeini, RezaGolestaneh, NadySharma, AmitGoljanek-Whysall, KasiaSharma, AnchalGollan, Peter J.Sharma, AnuragGolokhvast, KirillSharma, ArishyaGolstov, AlexeySharma, BheshGoltz, Douglas M.Sharma, DeepikaGolubovskaya, VitaSharma, GarimaGombert, AlexanderSharma, GeetanjaliGomes, Ana P.Sharma, IshaGomes, Maria SaloméSharma, KumariGomes, NelsonSharma, NeeruGomes, Paula A. C.Sharma, ShwetaGomes, SóniaSharma, SudhaGómez Castellà, CristinaSharma, SumanaGomez De Agüero, MercedesSharma, VirenderGómez De Cedrón, MartaSharman, Edward H.Gomez Quiroga, AdoracionShaul, Yoav D.Gomez, NidiaShavandi, AminGómez-Baena, GuadalupeShcharbin, DzmitryGomez-Casado, CristinaShe, MaoyunGomez-Florit, ManuelSheiner, LilachGomez-Gil, VeronicaSheka, Elena F.Gomez-Lobo, VeronicaShelper, Todd B.Gomez-Pinilla, Pedro J.Sheltzer, JasonGómez-Puertas, PaulinoShen, Jia ZackGómez-Zorita, SaioaShen, ShichenGomha, SobhiShen, ShihuaGonçalves, A PedroShen, WeiranGoncalves, OlivierShen, XingguiGoncharov, Nikolay V.Shen, XiulongGonda, SándorShen, YiGondi, Christopher SShenderovich, Ilya G.Gonec, TomasSheng, YueGongora Castillo, ElsaSher, Yuh-PyngGonkowski, SlawomirSheri, MadhuGonzalez Alvarez, IsabelSheridan, Robert P.Gonzalez, AidaShetty, Kateel G.Gonzalez, AntonioShevelyov, YuriGonzález, Florenci V.Shewale, SwapnilGonzalez, GabrielShewmaker, Frank P.Gonzalez, Juan M.Shi, ChunlinGonzález, MartaShi, HaifeiGonzalez, MartinShi, Jade JueGonzález, SegundoShi, JunfengGonzalez-Bulnes, AntonioShi, LeiGonzález-Díaz, HumbertShi, WenGonzàlez-Duarte, RoserShi, XiaowenGonzalez-Dunia, DanielShi, YiGonzalez-Escamilla, GabrielShiao, S. Pamela K.González-Fernández, LauroShiao, Young-JiGonzález-Lafont, ÀngelsShibata, HiroshiGonzález-Lezana, TomásShibata, ShigenobuGonzalez-Maeso, JavierShibata, TakumaGonzalez-Martin, CristinaShichita, TakashiGonzález-Minero, Francisco JoséShidal, ChrisGonzález-Navarro, HerminiaShigemura, YasutakaGonzalez-Vicente, AgustinShigeru, HishinumaGood, Deborah J.Shigeyoshi, YasufumiGoodheart, Jessica A.Shih, ChiahoGoodman, Alan G.Shih, Chun-KuangGoodman, Richard E.Shih, Ie-MingGopalakrishnan, GopanShih, Yu-RuGöpel, SvenShiina, TakashiGoppelt-Struebe, MargareteShim, DonghwanGóra, ArturShim, HyunboGorbacheva, LiubovShim, Joon W.Gorbatyuk, Oleg S.Shim, Kwan SeobGoreham, ReneeShim, SehwanGorgan, LucianShimada, YasuhitoGorgey, Ashraf S.Shimada, YohtaGorman, AdrienneShimaoka, MotomuGornowicz, AgnieszkaShimasaki, ToshiakiGorovits, BorisShimba, ShigekiGorrell, MarkShimizu, ShunichiGoschorska, MartaShimizu, TakeyukiGosciniak, GrazynaShimo, TsuyoshiGosmain, YvanShimokawa, NaofumiGossman, ToniShimosawa, TatsuoGoswami, AnandShimura, TatsuoGoswami, AnweshaShin, Chan YoungGoszczyński, TomaszShin, Dong-MyungGoto, KatsumasaShin, Kwang-SooGoto, RiekoShin, Vivian YvonneGoto, TaichiroShinde, SuhasGott, Jonatha M.Shindou, HideoGottesman, SusanShine, M BGottlieb, AlexandraShingu, TakashiGouveia, Ricardo M.Shinohara, AkiraGovaere, OlivierShinohara, MitsuruGovan, JosephShinozaki, JunichiGovindaraj, DhanapalShiomi, DaisukeGowran, AoifeShiota, SeijiGoyal, YogeshShiotari, AkitoshiGoyvaerts, CleoShirahata, TatsuyaGozem, SamerShirai, YasuhitoGrabelnych, Olga I.Shiraishi, IsaoGraber, Zachary TShishova, Maria F.Grabrucker, AndreasShmatov, MikhailGrabrucker, Andreas M.Shoemaker, Jason E.Gradia, DanielaShojaei, ShahlaGraeve, LutzShoji, IkuoGräf, RalphShoombuatong, WatsharaGraff, MariaelisaShortland, PeterGrafi, GideonShostak, AntonGraham, BrianShu, JiangGraham, JulieShu, XinhuaGraham, Nicholas AShukla, RuchiGramss, GerhardShulman, AdrianGramza-Michałowska, AnnaSiamaki, Ali R.Granado, M. DoloresSibilla, SaraGranados-Principal, SergioSibson, David RossGrand, Roger J.Siddappa, ByrareddyGrande Tovar, Carlos DavidSiddique, Abu BakarGrange, CristinaSiddiqui, ImranGrãos, MárioSideratou, ZiliGraphodatsky, AlexanderSidhu, SachdevGrässel, SusanneSidorova, YuliaGrasselli, ElenaSidoti, AntoninaGrasso, GianvitoSieber, Alisa-NaomiGrasso, GiuseppeSiebert, Hans-ChristianGrath, SonjaSiegel, JakubGrattagliano, IgnazioSielker, SonjaGrau, ElenaSiemianowski, OskarGraves, PatriciaSieniawska, ElwiraGraziano, GiuseppeSier, CornelisGrazioso, GiovanniSiewert, BiankaGrebenkov, Denis S.Sigal, DarrenGrebien, FlorianSignore, GiovanniGreco, AntonioSignore, MicheleGreco, SimonaSignori, EmanuelaGreen, HilarySikder, PrabahaGreen, MichaelSikorski, AleksanderGreenberger, Joel S.Sil, PayelGreene, Catherine M.Silaghi, Ciprian N.Greenhalgh, DavidŠiler, BranislavGreenwood, Michael T.Silikas, NickGreer, JohnSiliqi, DritanGreer, YoshimiSills, E ScottGreetham, DarrenSilva, Amelia MGreffrath, WolfgangSilva, Carla ManuelaGregg, RandalSilva, CatarinaGregori, AlbertoSilva, João PedroGregory, Stephen L.Silva, JoséGreither, ThomasSilva, Maria FátimaGrela, PrzemysławSilva, Nuno A.Grelet, SimonSilva, Pedro JorgeGrembiale, Rosa DanielaSilva, SaraGrendár, MarianSilveira, CéliaGrest, Gary S.Silveira, Nádya P. DaGrewal, Raji PaulSilver, Daniel J.Grewal, ThomasSilvestris, EricaGrewer, ChristofSilvestris, NicolaGrieco, DomenicoSim, ValerieGriesbeck, OliverSima, LiviaGriffiths, Cara A.Simakov, S. S.Grigelioniene, GiedreSimeoli, RaffaeleGrigoriadis, GeorgeSimes, DinaGrigorieva, Elvira V.Simkin, JenniferGrigorov, IlijanaSimko, FedorGrimm, Wolf-DieterSimm, StefanGriñán-Ferré, ChristianSimmen, Frank A.Gris, DenisSimmen, Rosalia C.M.Grisanti, Laurel A.Simoes, SandraGrisoni, FrancescaSimões, Zilá Luz PaulinoGrodzik, MartaSimon, Anne F.Groen, EwoutSimone, Angela DeGrolli, StefanoSimoni, JanGromiha M, MichaelŠimoník, OndřejGrondin, AlexandreSimonini, SaraGrones, PeterSimopoulos, ArtemisGroot, StevenSimopoulou, MaraGros, FrédéricSimova-Stoilova, LyudmilaGross, GideonSimova-Stoilova, Lyudmila PetrovaGroßhans, JörgSimpson, A. JohnGrosso, GiuseppeSimpson, Julie E.Grothe, ClaudiaSinclair, CharlesGrötzinger, CarstenŠindlerová, LenkaGroux-Degroote, SophieSingan, Vasanth R.Grubbs, NathanielSingh, AbhishekGrube, SusanneSingh, Amar M.Grubić Kezele, TanjaSingh, AmitGrudnik, PrzemyslawSingh, AnilGrul’ová, DanielaSingh, AnirudhaGrumezescu, Alexandru MihaiSingh, AnupGründker, CarstenSingh, ArunimaGrunebaum, EyalSingh, Ashok K.Gruntenko, NatalySingh, Brijesh KumarGruss, OliverSingh, ChandraGruszczynska-Biegala, JoannaSingh, ChanpreetGruszka, Alicja MSingh, DeoGrynkiewicz, GrzegorzSingh, Dr. KhushwantGrządziel, JarosławSingh, JugpreetGrzebelus, DariuszSingh, KamalendraGrzebelus, EwaSingh, KamayaniGrzela, RenataSingh, Kumar SaurabhGrzeskowiak, BartoszSingh, Manjeet K.Grzmil, PawełSingh, ManjulGrzymajlo, KrzysztofSingh, NarinderGu, FengSingh, NaveenGu, LiangcaiSingh, RajbirGu, LiuqiSingh, RajeshGu, YuchaoSingh, RatnakarGu, ZhenSingh, Reetu R.Guadagno, CarmelaSingh, SarbjitGuadagno, EliaSingh, ShaktiGualerzi, Claudio O.Singh, Surya PratapGuan, FengSingharoy, AbhishekGuan, JikuiSingla, BhupeshGuan, QingdongSinha Ray, SaikatGuarino, CarmineSinha, Birandra K.Guarino, FrancescoSinha, DebottamGubin, DenisSinha, JasmineGudey, Shyam KumarSinha, KalyanGudkov, Sergey V.Sinha, Sudarson SekharGuérardel, YannSiniscalco, DarioGuerra, CarmenSinkovic, AndrejaGuerra, FloraSipkens, Timothy A.Guerreiro, Joana FSipos, FerencGuerrini, GabriellaSipos, KatalinGugatschka, MarkusSiracusa, RosalbaGugliandolo, EnricoSirbu, OvidiuGugnoni, MilaSireci, GuidoGuguen-Guillouzo, ChristianeSiristatidis, CharalamposGuha, Prajna P.Siriwardena, Thissa N.Guha, PrasunSirko, SwetlanaGuha, SoniaSirobhushanam, SirishaGui, Shun-HuaSironen, TarjaGuibal, EricSirtori, CesareGuida, FrancescaSistla, SrinivasGuido, LuisSitarek, PrzemysławGuigon, CélineSivaguru, MayandiGuil, SòniaSivakumar, SushamaGuillermet Guibert, JulieSivalingam, PeriyasamyGuilloux, Jean-PhilippeSivanesan, IyyakkannuGuinamard, RomainSivasubramaniyan, KavithaGuirado, DamiánSiwiec, TadeuszGuirado, RamonSizochenko, NataliaGuix, Francesc XavierSizun, ChristinaGujas, BojanSjöberg, Bror FolkeGüldiken, NurdanSjoholm, AkeGuldimann, ClaudiaSkalniak, LukaszGulei, DianaSkelding, Kathryn A.Gulic, TamaraSkellam, ElizabethGullberg, DonaldSkendi, AdrianaGullì, MariolinaSkidmore, MarkGullner, GaborSkillbäck, TobiasGumfekar, SarangSkinner, Jonathan RGunaje, JayaramaSkoda, JanGunasekar, Susheel KumarSkoneczny, MarekGunawidjaja, RaySkorik, Yury A.Gundamaraju, RohitSkorko-Glonek, JoannaGuney, EmreSkorokhod, OleksiiGunia-Krzyżak, AgnieszkaSkorski, TomaszGunness, PurnimaSkotak, MaciejGünzel, DorotheeSkowrońska, KatarzynaGuo, BinSkrabana, RostislavGuo, Chih-HungSkriver, KarenGuo, DandanSkurikhin, Evgenii GermanovichGuo, FenghaiSlade, NedaGuo, FukunSlamova, KristynaGuo, LongbiaoSlawson, ChadGuo, WeiŚledziński, TomaszGuo, WeiminSleigh, James N.Guo, ZhenhengSlepian, MarvinGuo, ZhongmaoSliwinska, AgnieszkaGupta, BrijSliwinski, TomaszGupta, RajatSłoczyńska, KarolinaGupta, RaviSłomińska-Wojewódzka, MonikaGupta, SanjaySlominski, AndrzejGupta, ShuchiSłomkiewicz, Piotr MarekGupta, SouradeepSlootweg, Erik JGupta-Rossi, NeetuSlouka, ZdenekGurevich, Vsevolod V.Smeriglio, AntonellaGuriec, NathalieSmertenko, AndreiGururani, Mayank AnandSmetana, KarelGurzhiy, VladislavŚmieszek, AgnieszkaGus’kov, VladimirŚmiga-Matuszowicz, MonikaGusev, Nikolai B.Sminia, PeterGuskov, AlbertSmit, GuusGussago, CristinaSmit, LindaGussoni, EmanuelaSmith, CarolineGustav, StalhammarSmith, G. JasonGutiérrez, Juan CarlosSmith, JessicaGutierrez, SantiagoSmith, Lloyd M.Gutiérrez-Adán, AlfonsoSmith, Michael A.Gutsaeva, DianaSmith, Micholas DeanGuy, PaulSmith, PatriceGuyot, RomainSmith, RobertGuzek, DominikaSmykal, PetrGuzhova, Irina V.Smyth, JamesGuzik, MaciejSmyth, Lesley A.Guzmán, EduardoSmyth, RedmondGuzman-Puyol, SusanaSnegur, LubovGweon, Hyun SoonSnow, NicholasGyöngyösi, MariannSnyder, ChristopherH Shirvani, ArashSoare, AndreeaHa, Bo-KeunSoares, Paula IsabelHa, Ki-TaeSobacchi, CristinaHaarmann-Stemmann, ThomasSobhy, HaithamHaase, HajoSobierajska, KatarzynaHabyarimana, EphremSobotta, LukaszHada, MegumiSobotta, ŁukaszHadjiargyrou, MichaelSocała, KatarzynaHadjikakou, Sotiris KSocas-Rodríguez, BárbaraHadj-Rabia, SmailSofronov, GeorgyHa-Duong, TâpSohn, Jung WooHadži, SanSohn, Uy DongHaendler, BernardSoica, CodrutaHafizi, SinaSokoloski, KevinHagelueken, GregorSokolova, EvgeniyaHagerty, ChristinaSokolowska, MilenaHagland, Hanne R.Sola, MariaHaguet, VincentSolanki, SumeetHahm, BumsukSolano, FranciscoHahn, OliverSolecki, Wojciech BarnabaHaidar, SamerSoler, CarlesHaijes, HannekeSoler, David C.Haisma, HiddeSoler, Miguel ÁngelHalbritter, JanSolimando, Antonio G.Hald, Bjørn OlavSolimando, Antonio GiovanniHaldar, SumantoSolimena, MicheleHalder, Amit KumarSolinas, CinziaHallden, GunnelSoll, DieterHalldórsson, SkarphéðinnSolomon, MelaniHallerman, EricSolovchenko, AlexeiHallett, Maurice B.Solov’yov, AndreyHällgren, AnitaSoltani, MehdiHalloran, KieranSomani, SukrutHallstrom, Timothy C.Somarowthu, SrinivasHálová, IvanaSomasundaram, Dinesh BabuHamad-Schifferli, KimberleySommerhalter, MonikaHamaguchi, MasahideSomoza, RodrigoHamaguchi, ShogoSon, Deok-sooHamanishi, JunzoSonah, HumiraHamano, TadanoriSone, HidekoHamaoka, TakafumiSong, ChunhuaHamar, PeterSong, DanHamdan, Feda H.Song, GuoqingHamidi, TewfikSong, HailongHamieh, MohamadSong, Jong-WonHamming, JaapSong, JunqiHammock, Bruce.DSong, Lu-JieHammond, Timothy G.Song, MingHamsher, Sarah E.Song, MinjungHan, Byung WooSong, Min-SukHan, ChangseokSong, YoungsupHan, DongSong, ZiyuanHan, Dong-WookSoni, ChetnaHan, Hwa SeungSonntag, KaiHan, JaehongSoranno, AndreaHan, LinSorci, GuglielmoHan, MiraSørensen, Jens LauridsHan, PingpingSorg, OlivierHan, Sun-YoungSoriano, Jose MiguelHan, Yeon S.Soriano, SalvadorHanai, Jun-ichiSorli, Jorid B.Hanania, MichelSorokin, Vitaly A.Hanaoka, MitsumasaSorrell, J. MichaelHanas, JaySorrenti, ValeriaHancock, MeaghanSosic, IzidorHanda, OsamuSosna, JustynaHanda, Robert J.Sosnowski, Tomasz R. Haney, Conor M.Sota, TeijiHangelbroek, Roland W. J.Sotelo, Ana I.Hanif, MuhammadSoto, GabrielaHaniu, HisaoSoto, JulioHankir, Mohammed KSoto, PaulHannafon, BethanySoto-Reyes, ErnestoHano, ChristopheSottero, BarbaraHansen, Kirk C.Sottnik, JosephHansen, Mark BernerSoukup, AlesHansen, NielsSoulika, AthenaHansen, Peter RiisSoundharrajan, IlavenilHanson, BuckSounni, Nor EddineHanson, MaureenSourbier, CaroleHanson, Robert N.Sousa, AnaHantusch, BrigitteSousa, Sérgio F.Hanudel, MarkSousa, SofiaHanyková, LenkaSousounis, KonstantinosHao, JiaqingSoustiel, Jean-FrancoisHaque, InamulSouthgate, JenniferHara, AkiraSouto, ElianaHara, ToshifumiSouto, Eliana B.Harada, HisashiSovak, GuyHarada, MasakoSoveral, GraçaHaraguchi, RyumaSowa, IreneuszHaraguchi, TokukoSowndhararajan, KandhasamyHaraszthy, VioletSpakowicz, DanHardeland, RudigerSpang, ChristophHardies, Stephen C.Spanier, BrittaHardingham, Jennifer E.Sparvoli, FrancescaHardy, JohnSpassieva, Stefka D.Hardy, John GeorgeSpassov, SashkoHardy, PaulSpearman, PaulHardy, RowanSpeciale, AntonioHargreaves, IainSpella, MagdaHariharan, SeethalakshmiSpellman, PaulHarijith, AnanthaSpelman, LyndaHärkönen, PirkkoSpencer, Charles T.Harley, Vincent R.Spendiff, SallyHarmanci, Arif OzgunSperanza, GiorgioHarn, Horng-JyhSpierings, Diana C.J.Haro, DiegoSpiller, O. BradHarper, AlanSpinaci, MarcellaHarper, David R.Spingler, BernhardHarrington, CharlesSpittler, AndreasHarris, Andrew R.Spizzo, GilbertHarris, DanniSpizzo, RiccardoHarris, Edward N.Splichalova, AllaHarrison-Bernard, LisaSpratt, ThomasHarrop, RichardSprent, JanetHart, DavidSpriel, Annemiek VanHarthoorn, Lucien F.Spriggs, ElizabethHartley, Carol J.Spring, KevinHartman, MariuszSpringael, Jean YvesHartman, Matthew C.T.Sprio, Andrea ElioHartmann, Tanja N.Spyrakis, FrancescaHaruta, MiyoshiSquillacioti, CaterinaHarvey, AdamSquillaro, TizianaHarvey, AlexandraSquire, JohnHarvey, DonaldŠrámek, JanHarvey, PetaSreedasyam, AvinashHarvill, EricSreerama, SubramanyaHarvima, Ilkka T.Sresht, VishnuHasan, RaquibulSripathy, SmithaHasanuzzaman, MirzaSriram, GopuHasanzadeh Kafshgari, MortezaSrivastava, AkhilHascup, KevinSrivastava, AnkitHasegawa, HiromasaSrivastava, AtulHasegawa, MakotoSrivastava, PranayHasegawa, MorifumiSrivastava, Sanjay KHasegawa, Paul M.Srivastava, SaurabhHashimi, HassanStacchiotti, AlessandraHashimoto, MakotoStacey, MikeHaslam, IainStachewicz, UrszulaHassan, MohamedStachowiak, Michal K.Hassan, Sherif T. S.Stachowska, EwaHatakeyama, KatsunoriStafford, JamesHatakeyama, KazutoStafforst, ThorstenHatano, NoriyukiStagni, VenturinaHatzakis, NikosStäheli, PeterHatzikraniotis, EvripidisStahelin, Robert V.Hauser, PeterStahl, FelixHausman, Gary. J.Stambuk, NikolaHausmann, LudgerStana, AncaHausner, GeorgStanciu, Luminita A.Hautz, TheresaStanek, AgataHawi, ZiarihStaneva, DesislavaHawse, John R.Staneva, GalyaHayakawa, TohruStanford, Fatima CodyHayakawa, YokuStange, KatjaHayakawa, YoshihiroStaniak, MariolaHayashi, MakotoStanicová, JanaHayashi, Mariko KatoStanirowski, Paweł JanHayashi, NagaoStanislav, VosolsoběHayashi, ShinichiroStanisz, BeataHayashi, YoheiStankevičius, VaidotasHayden, HelenStanley, Jone A.Haynes, KarmellaStanley, RobertHaynes, Nicole M.Stapleford, Kenneth A.Hayward, Richard D.Starek, MałgorzataHazak, OraStark, Azadeh T.HAZAMA, ShoichiStärkel, PeterHe, AinaStarov, VictorHe, ChunboStarowicz, MałgorzataHe, FangStarr, Timothy K.He, FengStasiak, AndrzejHe, JingStasiak, MagdalenaHe, WeilueStathopoulos, ConstantinosHe, XinqiangStathopulos, PeterHealey, GarethStaudacher, DawidHealy, Luke M.Staunstrup, Nicklas HeineHearn, DavidStavrou, VasileiosHeath, P.R.Stawikowski, Maciej J.Heath, TimothyStawski, RobertHecht, Michael H.Stecco, CarlaHeckenhauer, JacquelineSteel, Laura F.Heckmann, Prof Dr JosefSteelmann, AndrewHector, Ralph D.Steenbergen, RenskeHeegaard, Peter M. H.Stefanaki, CharikleiaHefferon, Kathleen L.Stefanini, IreneHeffeter, PetraStefanon, BrunoHegarty, MatthewStefanovic, BrankoHegedűsová, AlžbetaStefanucci, AzzurraHegyi, PéterSteffen-Heins, AnjaHehlgans, StephanieStein, ColleenHeideman, DanielleStein, MatthiasHeinbockel, ThomasStein, ViktorHeindryckx, FemkeSteinbrenner, HolgerHeinrich, SandraSteinle, JenaHeitkam, TonySteinmann, Stephan NHejatko, JanSteinmeyer, JuergenHejátko, JanStelinski, Lukasz L.Hejčl, AlešStelmach-Mardas, MartaHejtmancik, J. FieldingStenzel, TomaszHeller, GerwinStepanenko, Aleksei A.Heller, JanoschStepanov, Gennady V.Hellewell, SarahStepanova, AnnaHellinger, RolandStéphanie, Miserey-LenkeiHelliwell, JohnStephanopoulos, NicholasHellmann, Jason L.Stepien, EwaHellmann, NadjaStępień, ŁukaszHellweg, ChristineStevenson, BradleyHendgen-Cotta, Ulrike B.Steward, RobertHendriks, WiljanStewart, AdeleHenen, Morkos A.Stewart, Jared J.Hengesbach, MartinStewart, JasonHenkel, JaninStieger, BrunoHenkin, Robert I.Stilli, DonatellaHenríquez, VitaliaStimac, DavorHenry, Ryan A.Stine, Keith J.Heo, Su-JinStobdan, TseringHer, Lu-ShiunStobiecki, MaciejHerath, VenuraStocco, CarlosHerichova, IvetaStocco, GabrieleHering-Smith, KathleenStockmann, ChristianHerman, Andrzej PrzemysławStojanović, SanjaHermans, EmmanuelStoker, AaronHermenean, AncaStolzenburg, Lindsay RHermoso, JuanStone, WendyHernandez, AgustinStoodley, Marcus A.Hernández, ÁngelStopka, TomášHernandez-Alonso, PabloŠtorchová, HelenaHernandez-Cerda, PedroStorey, Robert F.Hernández-Rodríguez, CésarStork, Christian J.Hernando, Carlos EstebanStorti, PaolaHerold, NikolasStossi, FabioHerraez, ElisaStout, Michael J.Herrera Paredes, SurStout, Michael JosephHerrero, María JesúsStowell, KathrynHerrero-Beaumont, GabrielSt-Pierre, BenoitHerskind, CarstenStrappe, PadraigHerskowitz, Jeremy H.Strasser, RichardHerten, MonikaStrati, FrancescoHerum, Kate MøllerStrati, KaterinaHerz, JosephineStrauss, LauraHess, AngelaStrauss, SarahHester, JoannaStringaro, AnnaritaHevey, RachelStrnadel, JanHewett, SandraStroffekova, KatarinaHeydemann, AhlkeStroh, AlbrechtHibaoui, YoussefStrohbach, AnneHicks, Julie A.Ström, LenaHidenobu, SoejimaStromberg, ZacharyHieda, MikiStrongin, RobertHietala, Ari M.Strudwick, XantheHigashida, HaruhiroStruga, MartaHiggins, Paul J.Strungaru, Stefan-AdrianHikosaka, KenjiStruzik, JustynaHildreth, BlakeStrygina, Ksenia V.Hilgemann, Donald WilliamStrzelecka-Kiliszek, AgnieszkaHilhorst, MarcStuart, JeffreyHill, MichaelStührwohldt, NilsHimeno, HyoutaStuper-Szablewska, KingaHindges, RobertStupka, NicoleHindra, HindraStur, ElaineHinds, Terry D.Sturlese, MattiaHines, Dustin J.Stutz, BernardoHines, Justin K.Stylianakis, Minas M.Hingorani, RittuSu, Chia-HaoHinkelbein, JochenSu, Chun-LiHinton, Shantá D.Su, MengHirabayashi, TetsuyaSu, YuanjieHiraga, ToruSuarez-Carmona, MeggyHirakawa, HidehikoSuárez-Varela, María M.Hirakawa, HidetadaSuau, PedroHirano, KatsuyaSubach, Fedor V.Hirano, NobutakaSubburaju, SivanHirano, ShigeruSubirats, XavierHirasaka, KatsuyaSubramaniam, DharmalingamHirasawa, NoriyasuSubramanian, ArulHirasawa, TakashiSubramanian, ChitraHirata, YokoSubramanian, SrinivasHiratani, IchiroSubramanian, VeedamaliHirata-Tsuchiya, ShizuSuciu, Bogdan AndreiHirayama, FumitoshiSud’ina, Galina F.Hiroaki, HidekazuSuda, KenichiHiroyuki, ShibataSuda, YoshihitoHirsch, PierreSudhan, DhivyaHirschi, KendalSudheeran, Pradeep KumarHiruma, KeiSuetsugu, NoriyukiHisada, YoheiSuffritti, Giuseppe BaldovinoHissa, BarbaraSuga, HirotakaHitachi, KeisukeSugahara, RyoheiHlavaty, JurajSugase, KenjiHluska, TomášSugawara, AkihiroHnilicka, FrantisekSugawara, AkiraHo CM, RogerSugimoto, YukioHo, Chia-cheSugimura, HaruhikoHo, HonSugita, MamoruHo, Jar-YiSugiyama, AkinoriHo, MengchiaoSugiyama, ToshihiroHo, MengfeiSugumaran, ManickamHo, RogerSuhail, YasirHo, Shin-LonSuidan, GeorgetteHo, VincentSujobert, PierreHoad, SteveSukhorukov, VladimirHoang, Nam VSukhov, VladimirHoang-Vu, CuongSukocheva, OlgaHobza, RomanSukseree, SupawadeeHodgkinson, James T.SUKUMARAN, ANILHodivala-Dilke, KairbaanSukumaran, SunithaHoepner, Lori A.Suliburska, JoannaHofbauer, StefanSullivan, Lucy C.Hoffmann, Klaus H.Sulová, ZdenkaHoffmann, MarkusSumczynski, DanielaHoffmann, Markus M.Sumi, ShoichiroHohenegger, MartinSummhammer, JohannHohensinner, PhilippSun, ChongkuiHohenstein, PeterSun, FengjieHoike, HarukiSun, GuohuiHoja-Łukowicz, DorotaSun, HaoHojo, HironoriSun, JieHojsgaard, DiegoSun, KunHolalu, SrinidhiSun, LemingHolban, AlinaSUN, MingKuanHoldt, Hans-JürgenSun, QianHole, DavidSun, RamonHolefors, AnnaSun, WeiHolen, IngunnSun, WujinHolguin, Francisco OmarSun, XiaofeiHolien, JessicaSun, XiaomingHolland, Linda Z.Sun, YingjieHolland, MartinSun, ZhonghuaHollenbach, MarcusSundar, Isaac KirubakaranHöllig, AnkeSundar, Krishna M.Holloman, WilliamSundar, ReshmaHolme, Jørn AndreasSundaram, Sivaraj MohanaHolmes, WilliamSundaramoorthy, PasupathiHolt, Scott M.Sundararajan, RajiHoltmann, DirkSundaresan, AlameluHolynska, MalgorzataSundram, SureshHolzmann, KlausSung, Jong HyukHomma, TetsuyaSung, Myong-HeeHonda, YoshitomoSung, Tzu-ChengHong, Chang-WonSung, Wen-WeiHong, Chien-HuiSungthong, RungrochHong, Seung-MoSunna, AnwarHong, Su-HyungSunseri, FrancescoHong, YonggeunSuphioglu, CenkHong, YuningSupuran, ClaudiuHonvo, GermainŠuput, DušanHood, DavidSurapathrudu, KanakalaHoover, Jennifer L.Surdutovich, EugeneHopkins, SamanthaSurguchov, AndreiHopp, RussellŠurinová, MáriaHoras, KonstantinSurmacz, LilianaHorejs-Hoeck, JuttaSurmenev, RomanHorhat, Florin GeorgeSusanne, KaeslerHori, KiyosumiSusila, HendryHorie, IchiroŠušor, AndrejHorim, LeeSutherland, Hamish S.Horinek, DominikSužiedėlienė, EditaHorn, PatrickSuzuki, HidenoriHorner, AndreasSuzuki, HideoHorowitz, John D.Suzuki, Hiroshi I.Horsburgh, AnnSuzuki, KeikoHorsch, Martin ThomasSuzuki, Masataka G.Hortle, ElinorSuzuki, NobuhiroHorvath, DavidSuzuki, NorioHorzmann, KatharineSuzuki, TakafumiHosgood, Sarah A.Suzuki, TomoHoshi, AkihikoSuzuki, ToshikazuHoshiba, TakashiSuzuki, YusukeHoshino, MasaruSvetashev, VasilyHoskins, ClareSvirshchevskaya, ElenaHosohata, KeikoSvoronos, Paris D.N.Hosoya, AkihiroSwayne, Leigh-AnneHosoya, ShoSweeney, BlakeHossain, Md KamalSwerczek, ThomasHossain, Mohammad ZakirSwiatecka-Urban, AgnieszkaHossain, MokarramSwiatkowska, AgataHosseini, Seyed AbdollahSwidergall, MarcHostiuc, SorinSwidsinski, AlexanderHosui, AtsushiŚwierczewska, MonikaHotea, IonelaŚwierniak, AndrzejHotta, YoshihiroSyed, NaeemHou, Chien-WeiSygitowicz, GrażynaHour, Mann-JenSyguda, AnnaHouse, ImranSykiotis, Gerasimos P.Houston, Kevin D.Syková, EvaHowell, StephenSymonds, Michael E.Howes, PhilipSymonova, RadkaHowland, John G.Synytsya, AndriyHozumi, KentaroSytykiewicz, HubertHrabal, RichardSzablewski, LeszekHrelia, PatriziaSzabo, AronHromadnikova, IlonaSzachniuk, MartaHryciw, Deanne H.Szaflarski, Jerzy P.Hsia, Shih-MinSzakiel, AnnaHsiao, Chung-DerSzakonyi, ZsoltHsiao, Pai-YiSzalai, ZoltanHsieh, ChengyingSzaleniec, MaciejHsieh, Chia-ChienSzczepanek, Steven M.Hsieh, Feng-ChiaSzczepaniec, AdaHsieh, Hsu-LiangSzczepanik, BeataHsieh, Ming KunSzczepanski, CarolineHsieh, Ming-ChingSzebeni, Gábor JánosHsieh, Pei-LingSzedlacsek, StefanHsieh, Tusty-JuanSzegletes, ZsoltHsieh, Yi-HsienSzeiffova Bacova, BarbaraHsu, Fei-TingSzekely, GyorgyHsu, Hsiu-FuSzeleszczuk, ŁukaszHsu, JonghauSzeliga, MonikaHsu, Kuo-ShengSzéll, MártaHsu, Ming-JenSzepesi, AgnesHsu, Shang-Te DannySzeto, Yim-TongHsu, Sung-PoSzewczyk, RomanHsu, ToddSzilágyi, AndrásHsu, Tsung-ISzilágyi, LászlóHsu, Tuan-TiSzklarz, GrazynaHsu, Yi-ChiungSzmatoła, TomaszHsueh, Yi-HuangSznajder, LukaszHu, ChenlinSzöllősi, DánielHu, GuangSzollosi, J.Hu, GuokuSzopa, AleksandraHu, GuoliSztal, TamarHu, JianSztuba-Solinska, JoannaHu, JunSzubert, SebastianHu, JunhuiSzweda, PiotrHu, KesongSzweda, PoitrHu, MinSzychlinska, MartaHu, Po-ShengSzychlinska, Marta AnnaHu, QinSzymanowska, UrszulaHu, TaoboSzymańska, GrażynaHu, WenweiTabata, YasuhikoHu, Wen-YangTabish, TanveerHu, XiaoyuTabolacci, ElisabettaHu, YangTabuchi, MasashiHu, YuanTacke, FrankHu, Yueh-ChiangTada, RuiHu, ZhenbinTada, YuichiHuang, ChangjunTadepalli, SirimuvvaHuang, Chao-ChengTadyszak, KrzysztofHuang, Chiu-jungTae-Jin, KimHuang, Guan-JhongTafani, MarcoHuang, HaoTafuri, SimonaHuang, Hsiao-YunTaga, ArensHuang, Jason C.Tagad, HarichandraHuang, Jason H.Taghibiglou, ChangizHuang, Kuang-TzuTaglietti, AngeloHuang, LinfengTaglietti, Angelo MariaHuang, Li-TungTaglietti, ValentinaHuang, Nai-KueiTago, KenjiHuang, Paul Li-HaoTaguchi, Y-h.Huang, Shau-KuTaguchi, YuzuruHuang, Shih-YiTaheri, ShimaHuang, TianyiTai, HelenHuang, Tsui-ChinTai, WanboHuang, Tzu-TingTaillandier, DanielHuang, Wei-ChienTaishi, NakamuraHuang, WeihuaTait Wojno, EliaHuang, WeijieTaji, TeruakiHUANG, Wen-ChinTajiri, NaokiHuang, Wen-LiiTakabe, WakakoHuang, Wu-JangTakač, IztokHuang, XiaoTakac, TomasHuang, XiaohuTakács, ZoltánHuang, XuTakada, IchiroHuang, Ya-lingTakada, TappeiHuang, YifengTakagi, DaisukeHuang, YongjuTakagi, KiyoshiHuang, Yung-FenTakagi, MasatoshiHuang, ZiweiTakahashi, AkihisaHubacek, Jaroslav ATakahashi, AkiyoshiHuber, RTakahashi, HideyukiHuber, RobertTakahashi, KenHuber, VictorTakahashi, NobuyukiHubert, SytykiewiczTakahashi, ShinyaHubková, BeátaTakahashi, TakuHuchzermeyer, BernhardTakahashi, TetsuyaHuerta-Angeles, GloriaTakahashi, ToshioHueso, MiguelTakai, YasushiHughes, GabrielTakaki, ManabuHuh, Jin HoeTakakura, KazukiHuh, Jung-BoTakamatsu, DaisukeHuhn, RagnarTakamatsu, ShigeyukiHui, Kwai FungTakano, KatsuraHulleman, JohnTakasawa, KenHumbeck, KlausTakasawa, ShinHummel, MaryTakashiba, ShogoHummitzsch, KatjaTakashima, EizoHummler, EdithTakashima, YuyaHumphreys, DavidTakáts, SzabolcsHumphreys, IanTakatsuka, JunHumphries, BrockTakayama, FusakoHumphries, Jonathan D.Takayama, KenichiHung, AndrewTakayama, YasunoriHung, Chin-ChuanTakeda, KojiroHung, Jan-jongTakeda, MakotoHung, Jung-TungTakegahara, NorikoHung, Ming-SzuTakemiya, TakakoHung, Shih-YaTakenaka, MizukiHunt, HarrietTakeshi, KurokuraHunt, PamelaTakeuchi, HideyukiHunter, Wayne B.Takeuchi, TomoharuHuntosova, VeronikaTakeya, SatoshiHuntošová, VeronikaTakiya, ShigeharuHuo, YudaTakizawa, NaokiHuppertz, BertholdTakó, MiklósHuppi, KonradTakuma, KazuhiroHur, HoonTakumi, ShigeoHur, JungukTakyar, SeyedtaghiHur, WooyoungTalamonti, EmanuelaHur, YoonkangTalari, Abdullah Chandra SekharHura, TomaszTalavera, KarelHurst, Brett L.Talbert, ErinHurtado, AntoniTallmadge, RebeccaHusnjak, KoraljkaTalora, ClaudioHussain, KhalidTalukder, PoulamiHussein, WaleedTaly, AntoineHutchison, Robert E.Tamagawa-Mineoka, RisaHutter, GregorTamagnone, LucaHutter, MichaelTamaki, TetsuroHuwiler, AndreaTamba, Bogdan IonelHvattum Divon, HegeTambe, MitaliHviid, ThomasTamminen, TarjaHwa Soung, YoungTampa, MirceaHwang, In KooTamura, KouichiHwang, NathanielTamura, MasaakiHwang, YongsungTamura, OsamuHybiske, KevinTamura, TomokoHyer, SteveTan, Bee KHyndman, Kelly AnneTan, Bee KangHyun, Sang-HwanTan, Du-XianIACCARINO, IngramTan, IsabellaIacono, DiegoTan, JoanneIampietro, MathieuTan, KarenIan, DundasTan, LongzhiIannelli, M. A.Tanabe, HideyukiIannuzzi, ClaraTanabe, KatsuyaIantcheva, AneliaTanabe, KatsuyukiIasevoli, FeliceTanabe, KenjiIbarra Molero, BeatrizTanabe, ShihoriIbarretxe, GaskonTanabe, YooIbdah, MwafaqTanaka, EijiIbiza, SalesTanaka, HisashiIbrahim, AhmedTanaka, IchidaiIbsen, StuartTanaka, MasashiIchida, KimiyoshiTanaka, NaoroIchihashi, MasamitsuTanaka, NobuyukiIchikawa, HiroshiTanaka, SatoshiIchitani, KatsuyukiTanaka, ShigenoriIciek, MałgorzataTanaka, TakujiIdo, YasuoTanaka, ToshiakiIdogawa, MasashiTanaka, Yasuoidris, adiTanaka, YoshikazuIdrissi, OmarTanase, CorneliuIentile, RiccardoTanase, CristianaIgamberdiev, Abir U.Tandra, Pavan KumarIglesias-Osma, Maria CarmenTang, AihuaIgnatov, AlexanderTang, BorIhee, HyotcherlTang, Chih-HsinIijima, NoriakiTang, JingIimura, TadahiroTang, JonathanIizuka, KatsumiTang, KaiIizuka, ShinjiTang, Kwan HoIjichi, HideakiTang, LongtengIkeda, KeigoTang, MinghuaIkeda, MasahiroTang, RenjieIkeda, YoukoTani, EleniIkegaya, NaokiTanic, NikolaIkehara, KenjiTanigawa, TetsuyaIkeogu, UgochukwuTaniguchi, HiroakiIkura, YoshihiroTaniguchi, KoheiIlag, Leodevico L.Tanimizu, NaokiIlgu, HuseyinTanini, MarcoIliescu, CiprianTankiewicz-Kwedlo, AnnaIllangakoon, U.ErankaTanner, NathanIllera, Juan CarlosTanner, S.BoboIlluminati, GiulioTannous, JoannaIlyinskii, Petr O.Tanonaka, KouichiIm, Chang-NimTantos, ÁgnesImai, TomoyaTanuma, Sei-ichiImai, ToshioTanveer, MohsinImai, YoichiTao, ChenqiImai, YuzuruTao, FengImamoto, YasushiTao, JunyanImamura, MasahiroTao, W. AndyImamura, TeruhikoTapia, Alejandro A.Imbriano, CarolTapia, Jose A.Imbrici, PaolaTappa, KarthikImgenberg-Kreuz, JulianaTappia, ParamjitImhof, PetraTarakhovskaya, ElenaImig, JohnTarantino, GiovanniImlach, Wendy L.Tarantul, Vyacheslav Z.Impellizzeri, DanielaTardin, CatherineInagaki, NoritoshiTari, IrmaIncitti, TaniaTarkowski, PetrIndiveri, CesareTarn, Woan-YuhInfante, ArantzaTarozzi, AndreaInfante, PaolaTartera, CarmenInga, AlbertoTartey, SarangInglada-Pérez, LucíaTashima, KimihitoInnocenti, NicolasTashima, ToshihikoInomata, TakenoriTashiro, HirotakaInoue, AtsukoTate, Shin-IchiInoue, HirofumiTatebe, HisashiInoue, KazukiTatsuke, TsuneyukiInoue, KazushiTatsumi, KoukoInoue, Ken-ichiroTatsuya, KatoInoue, SatoshiTattikota, Sudhir GopalInoue, YasumichiTatu, Alin LaurenţiuInoue, YusukeTatulian, Suren A.Insenser, MaríaTatullo, MarcoInyushin, Mikhail Y.Taub, MaryInzana, Thomas J.Taurin, SebastienIommarini, LuisaTavaniello, SiriaIommelli, FrancescaTavanti, FrancescoIop, LauraTavares, AnthonyIorio, Marilena V.Tavares-da-Silva, Elisiario JoséIotti, StefanoTawara, KenIourov, Ivan Y.Tayal, AkhilIpek, ÖzlemTayebati, Khosrow SayedIram, SurtajTaylor, Briana J.Irato, PaolaTaylor, CarolineIrish, ErinTaylor, KathyIrving, TomTaylor, NicholasIshibashi, KenichiTaylor, Nicolas L.Ishigami, TomoakiTaylor, Stephanie R.Ishii, IsaoTchertanov, LubaIshii, KenichiroTchetina, Elena V.Ishii, MasakazuTchoghandjian, AurelieIshii, TetsuroTedbury, PhilipIshijima, SumioTedesco, IdoloIshikawa, RyujiTeeri, TeemuIshikawa, YasuyukiTei, LorenzoIshimoto, TakatsuguTeichmann, ThomasIshitsuka, YoichiTeif, VladimirIshizaki, TakumaTeixeira, João M. C.Isidori, AlessandroTeixeira, RitaIskratsch, ThomasTeixidó, ElisabetIslam, MahmudaTéletchéa, StéphaneIslam, Md. TabibulTeli, Mahesh KumarIslam, Mohammad MirazulTellez-Gabriel, MartaIslam, SyedTellier, MichaelIsmael, M. IsabelTemel, AslihanIsmail, Mohd NazriTempfer, HerbertIsola, GaetanoTemraz, SallyIson, Michael G.Teng, Ba-BieIsozaki, TakeoTeng, JianItakura, YokoTeng, YangItatani, YoshiroTeng, YongItenov, Theis SkovsgaardTenkorang, MavisIto, HiroyasuTennstedt, PierreIto, NaokiTentscher, PeterIto, ShosukeTeo, JeremyIto, TakamichiTEO, Wei SuongIto, TasukuTeodoro, Anderson JungerIto, YasuhiroTeotia, SachinItoh, TohruTeparić, RenataItokazu, YutakaTeperino, RaffaeleItou, JunjiTerai, YoheiIvana, VucenikTeraishi, MasayoshiIvancic-Bace, IvanaTeramachi, JumpeiIvanov, AlexanderTeramoto, HidetoshiIvanov, AndreiTeramoto, NaozumiIvanov, IvanTeras, RihoIvanov, RumenTerekhova, Irina V.Ivanov, SergeyTerhonen, EevaIvanova, BojidarkaTermolino, PasqualeIvanovitch, Kenzo D.Terpou, AntoniaIvashchenko, DmitriyTerra, XimenaIvics, ZoltánTerracciano, DanielaIviglia, GiorgioTerriente, JavierIwamoto, HiroyukiTeruyuki, TANAKAIwamoto, NaokiTervahauta, Arja IIwamoto, SadahikoTerzaghi, WilliamIwamoto, ShotaroTerzaghi, William BryanIwaoka, MichioTeschke, RolfIwasa, ToruTessier, FredericIwasaki, HironoriTesta, UgoIwasaki, TakashiTestai, FernandoIwasaki, YusakuTestolin, RaffaeleIwata, JunichiTetens, JensIwata, TakanoriTeter, KenIwata, YujiTeves, María EugeniaIyer, HariniThackray, AlanaIzadyar, AnahitaThalamuthu, AnbupalamIzasa, HisashiThammina, Chandra SekharIzawa, Kazuhiro P.Than, Nandor GaborIzawa, TakashiThangaraj, AnnaduraiIzawa, TakeshiThanigaimani, ShivshankarIzdebska, MagdalenaThanikachalam, KannanIzquierdo-Rico, Maria JoséThapa, Raj KumarIzuhara, KenjiThapar, RoopaJ. Storkus, WalterTharabenjasin, PhuntilaJaaro-Peled, HannaThe, ErlindaJabłonowski, ZbigniewTherese, RiedemannJablonska, EwaTheurich, SebastianJablonska, KarolinaThevananther, SundararajahJacevic, VesnaThevenin, DamienJackman, Jane E.Thevenot, Paul T.Jackson, Stephen A.Thi, Mia MiaJackson, William F.Thiel, GerhardJacob, ChristopheThiel, TeresaJacob, FrancisThiele, WilkoJacob, NoamThielen, BethJacobs, Elizabeth RThierry, BenjaminJacobson, Kenneth A.Thilaganathan, BaskyJacomin, Anne-ClaireThilmony, RogerJacquel, ArnaudThines, EckhardJaeger, LucThiruvengadam, MuthuJaehne, Anja KathrinThoma, Markus H.Jaggupilli, AppalarajuThomas, Seddon Y.Jagus, RosemaryThomas, T.J.Jaguva Vasudevan, Ananda AyyappanThomas, WayneJahan-Mihan, AlirezaThompson, BarryJähn, KatharinaThompson, Henry J.Jahr, HolgerThompson, JeffreyJaimes, Edgar A.Thompson, JoyceJain, AnkurThompson, Loren P.Jain, VaibhavThompson, Miles D.Jaing, Tang HerThompson, RichardJaiswal, Ashvin R.Thoms, SvenJaiswal, DeepikaThomson, David M.Jakas, AndrejaThongprayoon, CharatJakasa, IvoneThorne, James L.Jakiela, BogdanThornton, CatherineJakovljevic, VladimirThorpe, ChavaunneJakubczyk, AnnaThummuri, DineshJalaludin, AdamThuzar, MoeJalgaonkar, SwatiTian, BoJalkanen, SirpaTian, JiangJambrina, Pablo G.Tian, LiJames, Euan KevinTian, MiaoJames, MargaretTicconi, CarloJames, Nicholas G.Tidgewell, KevinJames, WilliamTien, Lu-TaiJamet, ElisabethTierney, Keith B.Jamin, NadègeTietjen, IanJamwal, RohitashTiffen, Jessamy C.Jana, SvobodováTigabu, MulualemJanakiraman, HarinarayananTigyi, GaborJancsó, GáborTihkonov, VladimirJanczar, SzymonTiidus, PeterJanczarek, MonikaTikhonov, VladimirJanda, TiborTillekeratne, L.m. VirangaJandl, KatharinaTilley, MichaelJanecki, MarcinTiminszky, GyulaJang, ByungkiTimmins, JoannaJang, Hyeung-JinTimmons, Lisajang, JinahTimoshenko, AlexanderJang, Jyan-ChyunTimson, David J.Jang, SeonghoeTirelli, UmbertoJanga, MadhusudhanaTiribuzi, RobertoJaniak, AgnieszkaTirino, VirginiaJanicka-Russak, MałgorzataTiriveedhi, VenkataswarupJanik, KatrinTischkau, Shelley A.Janiuk, IzabelaTischler, DirkJanjetovic, ZoricaTisné, CarineJankauskaitė, LinaTithi, Jesmin JahanJankovič, Ľuboš Š.Titorencu, Irina DomnicaJankowicz-Cieslak, Joanna BeataTitorenko, VladimirJanousek, BohuslavTitus, MarkJanowiak, Blythe E.Tiu, Brylee DavidJanowski, MiroslawTiwari, Rakesh K.Janssen, DirkTiwari, VijayJara Palacios, María JoséTo, Kin-YingJargin, Sergei VTobin, Desmond J.Jariel-Encontre, IsabelleTobrman, TomášJarome, TimothyTocmo, RestitutoJaroszuk - Ściseł, JolantaToczylowska-Maminska, RenataJarosz-Wilkołazka, AnnaTodaka, DaisukeJarzab, BarbaraTodeschini, Anne-LaureJasinski, MarcinTodeschini, RobertoJat, Parmjit S.Todorova, DessislavaJaudzems, KristapsTogawa, AtsushiJaumot, JoaquimTognon, CristinaJauregui, CatherineToh, Tan BoonJauregui, Rodrigo GutierrezToh, Wei SeongJauset, MiriamToietta, GabrieleJavaheri, TaherehToillon, Robert-AlainJawhara, SamirToiu, AncaJayakumar, ThanasekaranTokarek, TomaszJayaprakash, PriyamvadaTokas, TheodorosJayaraman, AnushaTokuda, GakuJazirehi, Ali R.Tokuda, ShinsakuJean, LudovicTokunaga, AkinoriJeandet, PhilippeToldrá, FidelJee, JunGooTolosa, EzequielJeffery, ElizabethTolvanen, Martti E.E.Jellinger, Kurt A.Toma, MilanJelonek, KatarzynaTomakidi, PascalJeltsch, AlbertTomanin, RosellaJembrek, Maja JazvinšćakTomar, DhanendraJena, Prasant KumarTomas, AlejandraJendelova, PavlaTomasetto, CatherineJenei-Lanzl, ZsuzsaTomassetti, AntonellaJenkins, ColinTomaszewska, EwaJenny, Titus A.Tomatsu, ShunjiJensen, Eric D.Tomaz, Cândida TeixeiraJensen, Jacob KrügerTomczykowa, MonikaJensen, Rebekka VibjergTomi, FélixJensen, RobertTomilin, Alexey N.Jeon, SoheeTominaga, MotokiJeong, Byung-HoonTomita, YusukeJeong, JaeWookTomiyama, ArataJeong, Keun-YeongTomkin, GeraldJeong, SekyooToms, DerekJeong, Seon-YongTomuleasa, CiprianJerebtsova, MarinaTona, FrancesoJeremić, SanjaTong, JessicaJerman, IgorTong, JunchaoJerónimo, CarmenTong, qiangJerzak, Malgorzata MariaToniolo, Antonio Q.Jeschke, UdoTonolo, GiancarloJetten, AntonTonooka, YukiJeung, Eui-BaeToomer, Gabriela Chavez-CalvilloJežek, JanToorie, AnikaJezierski, JanTop, DenizJhanwar, ShaluTopalidou, EleniJi, HezhaoTopin, JeremieJi, PengToppila-Salmi, SannaJi, ShaofeiToranzo, Alicia E.Ji, YuelongTordai, AttilaJia, DongyaToriba, TaiyoJia, LeijieTorigoe, KanjiroJia, YongTornesello, Anna LuciaJian, XingTornesello, MariaJiang, JianxiongTorok, MariannaJiang, Lin-HuaTorras-Ambròs, JoanJiang, PeiyongTorre, Maria LuisaJiang, Qiu-XingTorre, MarialuisaJiang, ShiboTorres Aleman, IgnacioJiang, Shu-YeTorres, DanielJiang, WeiTorres, MagdalenaJiang, XinyinTorres, Miguel AngelJiang, XiqianTorres-Ayuso, PedroJiang, YongTorres-Lagares, DanielJiao, ChenTorres-Reveron, AnnelynJifon, JohnTorres-Ruiz, RaulJimbo, MitsuruTorricelli, RenzoJimenez, JavierTorriglia, AliciaJiménez-Alemán, GuillermoToruner, Gokce AltayJiménez-Barbero, JesusTósaki, ÁrpádJiménez-Jiménez, Félix JavierToscani, DeniseJimi, EijiroTosco, AlessandraJin, Dong-HoonTošner, ZdeněkJin, XinTosolini, AndrewJin, YiTosti, ElisabettaJing, RanTotero Gongora, Juan SebastianJinno, YoheiToth, Balazs IstvanJirkovsky, EduardToth, EricJochum, ChristophTóth, TündeJoglekar, MugdhaTothova, CsillaJohansson, Martin E.Totta, PierangelaJohansson, Mats W.Tourniaire, FranckJohansson, MikaelToussaint, ArianeJohansson, PegahTovoli, FrancescoJohn, ShaliniTowers, ChristinaJohnsen, Kasper BendixToyoda, MasashiJohnson, Colin P.Toyomasu, TomonobuJohnson, DanielTrabace, LuigiaJohnson, Dylan M.Trachtman, HowardJohnson, EricTramontano, DonatellaJohnson, JoshuaTran, Anh NhatJohnson, Lance A.Tran, CuongJohnson, R. JeremyTran, KennethJohnson, Shannon L.Tran, KimJohnston, SimonTran, LangJohnstone, Scott R.Tran, PhuJoles, Jaap A.Tranbarger, Timothy JohnJolly, Mohit KumarTrapani, DarioJonathan, CummingTrapani, ValentinaJones, BleddynTravaglione, SaraJones, Kathryn MarieTravelli, CristinaJones, MarjorieTravlos, IliasJones, MartinaTraw, Milton BrianJones, MerielTraynor, DamienJordão, António ManuelTrcek, JanjaJørgensen, KirstenTrdan, StanislavJorrin-Novo, Jesus VTreebak, JonasJosef, KapfhammerTreeck, OliverJoseph, Laurie B.Tremblay, NicolasJoshi, AmitTrentini, AlessandroJoshi, HirenTresguerres, JesúsJoshi, KamalTrevisan, AndreaJoshi, Pushpa RajTriaca, VivianaJu, GaoTriantafillidis, John KJu, HeongkyuTriantis, Dimos A.Ju, XinshengTribble, James R.Juang, Shin-HunTribulova, NarcisJuárez-Maldonado, AntonioTricarico, DomenicoJuarranz, YasminaTrichet, ValérieJuhasz, BelaTriebl, AlexanderJuhász, TamásTrier, Nicole H.Juif, Pierre EricTrigatti, BernardoJullian, NathalieTriggle, Christopher RobertJulve, JosepTrilling, MirkoJun, Bong-HyunTrimarco, BrunoJung, ChaeyongTrinchera, MarcoJung, Chol-HeeTrincone, AntonioJung, Ho-SupTripathi, DiwakerJung, Hye JinTripathi, Jitendra KumarJung, Hyun-DoTripathi, ManishJung, JayongTripathi, Satyendra C.Jung, Ji HoonTripi, GabrieleJung, Ki-HongTripodi, PasqualeJung, Sung ChulTrivedi, PankajJungnickel, HaraldTrivedi, RachanaJunker, AndreasTrnka, PavelJuranek, Judyta KarolinaTroczka, BartlomiejJurkowska, HalinaTrofimov, Aleksei V.Juszczak, LesławTroitsky, AlexeyKaarniranta, Kai KaiTrombetta, DomenicoKabała-Dzik, AgataTroppmair, JakobKabisch, StefanTroutman, Jerry M.Kachamakova-Trojanowska, NeliTrovato, FabioKaczor, Agnieszka A.Troy II, Frederic A.Kadam, AvinashTrubiani, OrianaKadam, SuhasTrujillo, CristinaKadenbach, BernhardTruong, Nghia P.Kadife, ElifTryggestad, JeanieKadioglu, OnatTsai, Ang-ChenKaeffer, BertrandTsai, Chia-LiangKafarski, PawelTsai, Kun-LinKageyama, YukioTsai, Kuo-WangKagota, SatomiTsai, Lo-LinKahlert, StefanTsai, RongkungKaina, BerndTsai, Shiao-WenKAINOH, YooichiTsai, Shih-JenKaiser, AnnetteTsai, Shu-HuaiKaja, SimonTsai, Yuan-ChenKajiura, HiroyukiTsai, Yu-ChangKajiya, HiroshiTsaltas, DimitrisKajiya, MikihitoTsang, Kwong YokKajiyama, HiroshiTsapogas, PanagiotisKakehashi, AnnaTschernig, ThomasKakisis, John D.Tschon, MatildeKakizaki, SatoruTse, William K.F.Kakrana, AtulTse-Dinh, Yuk-ChingKálai, TamasTsiolaki, Paraskevi L.Kalani, KomalTsirigotis, PanagiotisKalas, WojciechTsitsekian, DikranKalathiya, UmeshTsitsilonis, Ourania E.Kalbacova, MarieTsolakis, Apostolos V.Kale, AbhijitTsotsalas, ManuelKale, Shiv D.Tsoukalas, NikolaosKalemba, Ewa M.Tsoulfas, GeorgeKalen, AmandaTsouvaltzis, PavlosKalendar, RuslanTsubaki, KazunoriKalhan, SatishTsuboi, NaotakeKaliniewicz, ZdzisławTsubota, TakuyaKalinina, OlgaTsuchida, KunihiroKalkman, Hans OTsuchida, SachioKalland, Karl-HenningTsuchiya, HiroyukiKaller, MarkusTsuchiya, KyoichiroKallikourdis, MarinosTsuchiya, MasahikoKalogirou, CharisTsuchiya, YuichiKalra, DivyaTsuda, HirokoKalve, ShwetaTsui, Kuan HaoKalyandurg, Pruthvi BalachandraTsui, Stephen Kwok-WingKamath, DivyaTsuji, Atsushi B.Kamath, KarthikTsuji, GakuKamei, KaekoTsuji, RyoheiKamennaya, NinaTsujita, TadayukiKameshima, SatoshiTsukahara, ToshifumiKamihira, MasamichiTsukamoto, DaisukeKamijo-Ikemori, AtsukoTsukita, SachikoKamil, JeremyTsurkan, Mikhail V.Kaminaka, HironoriTSUTSUI, MakusuKaminer, Michael S.Tsutsui, ShigeyukiKaminski, RafalTsytsarev, VassiliyKaminskyy, VitaliyTu, MinKamiya, TakehiroTu, ZhidongKammerer, TobiasTuanyok, ApichaiKamnev, AlexanderTubaro, AureliaKamolz, Lars-PeterTucci, Chiara DiKamysz, WojciechTuccinardi, TizianoKanakis, GeorgeTucker, Thomas J.Kanakry, Christopher G.Tudela, JoseKanamarlapudi, VenkateswarluTufa, Ramato AshuKanamori, TakaoTuleu, CatherineKanasaki, KeizoTulkens, PaulKanazawa, MasatoTullio, VivianKanczler, JanosTuma, RomanKanda, TatsuoTunaru, SorinKandasamy, RamTundo, SilvioKanekiyo, TakahisaTung, Tao-HsinKaneko, GenTuorto, FrancescaKaneko, JunTupally, Karnaker R.Kaneoka, HidenoriTurano, MimmoKanerva, MikkoTurano, PaolaKang, Bum-yongTurato, CristianKang, Byoung-CheorlTuriel, MaurizioKang, ChenTurk, TomKang, CongBaoTurkina, Maria V.Kang, DongchulTurkman, NashaatKang, Hee Y.Türkoğlu, OnurKang, HyojeungTurks, M.Kang, JunsuTurnaturi, RitaKang, Kyung PyoTurnbull, Matthew W.Kang, KyungsuTurner, Bradley J.Kang, Min-JungTurner, ClaireKang, Sang SunTurner, NeillKang, Sun-WoongTuroverov, Konstantin K.Kang, Tae-HongTurrini, EleonoraKang, WeiTurroni, SilviaKang, Young-HeeTuttolomondo, AntoninoKang, YunTuvdendorj, DemidmaaKango-Singh, MadhuriTuz, RyszardKankaanpää, PasiTvedt, Tor HenrikKanno, HiroshiTwu, Yuh-ChingKanno, TakahiroTyagi, SonikaKanno, YosukeTyler, RobertKanoi, BernardTyrka, MirosławKansara, MayaTzanakakis, George N.Kanugula, AnanthaTzeng, Shiang-JongKanzaki, HiroyukiTzika, Athanasia C.Kao, Shih-HanUberti, FrancescaKapcia, Konrad JerzyUchida, SatoshiKapitsinou, PinelopiUchida, ShizukaKaplan, Joshua M.Uchino, JunjiKaplan, ShaunaUddin, Golam MezbahKappachery, SajeeshUeda, AkihiroKarakasidis, TheodorosUeda, MinoruKaram, SherifUeda, YoshiakiKaraman, SinemUehara, YoshinariKaranis, PanagiotisUeno, Shu-ichiKarantanos, TheodorosUeno, YujiKarasiński, JanuszUfnal, MarcinKarcz, WaldemarUgarte-Berzal, EstefaniaKarczmarzyk, ZbigniewUhde-Stone, ClaudiaKardassis, DimitrisUhl, EberhardKardos, JózsefUhrig, R. GlenKarihtala, PeeterUhrikova, DanielaKarim, NasiaraUimari, AnneKarkanis, AnestisUkic, SimeKarlsson, JoakimUlasov, IlyaKarmakar, ParthaUldrijan, StjepanKarmazyn, MorrisUlf, KahlertKarnati, Konda ReddyUlicna, LiviaKarolkiewicz, JoannaUllah, Md AshikKarpinski, Tomasz M.Ullrich, OliverKartsonaki, ChristianaUmar, ShahidKarunakaran, SuneeshUmehara, TakuyaKarwowski, Boleslaw T.Umemori, JuzohKasahara, HidekoUmemura, AtsushiKasahara, Ryushiro D.Umemura, MycoKasai, FumioUnderhill, Suzanne M.Kaschula, Catherine H.Unfried, KlausKashapov, Ruslan R.Ungaro, PaolaKashfi, KhosrowUngefroren, HendrikKashimada, KenichiUngerstedt, Johanna S.Kashiwagi, ShinichiroUnune, Deepak RajendraKashiwakura, IkuoUpadhyay, RakeshKashtalap, VasilyUpadhyay, Rakesh K.Kashuba, ElenaUpadhyaya, JasbirKaskel, FrederickUpdegrove, Taylor B.Kasper, ClaudiaUpham, Brad L.Kasperkiewicz, PaulinaUppulury, KarthikKasprzak, AldonaUrbach, ValerieKassai, HidetoshiUrbain, AurélieKassan, AdamUrban, OtmarKassan, ModarUrban, PhilippeKasson, MatthewUrbańczyk-Lipkowska, ZofiaKasuya, YoshitoshiUrbanelli, LorenaKatahira, MasatoUrbini, MilenaKatahira, RuiUrra, FelixKatam, RameshUrra, José MiguelKataoka, HiroshiUshakov, Nikolay M.Kataoka, NaoyukiUsmani, AbulKatayama, KentaroUsmani, ShariqKateřina, SchwarzerováUsui, KenjiKathuria, HimanshuUsui, TatsuyaKato, HirohitoUsuki, YoshinosukeKato, KoichiUversky, VladimirKato, Takamitsu AUyeda, TaroKato, YasumasaUygun, SahraKato, YusukeUziel, OritKatoh, HironoriV Ivanov, AlexanderKatoh, KazuoVaandrager, Arie B.Katona, EvaVacca, MicheleKatsanos, Aristeidis H.Vaccarezza, MauroKatselis, GeorgeVáclavíková, RadkaKatsila, TheodoraVadlapudi, Aswani DuttKatsoris, PanagiotisVafiadaki, ElizabethKatsumoto, YukiteruVagner, MarieKatwal, DikshaVago, RiccardoKatz, Ben Z.Vaidya, AmitaKaufman, YairVaidyanathan, GanesanKaundal, RavinderVaidyanathan, SriramKauppinen, AnuVaiman, DanielKaur, AmanpreetVairetti, MariapiaKaur, HarleenVajro, PietroKaur, JasmineValable, SamuelKaur, SimranjeetValabrega, GiorgioKaushik, PrashantValach, MatusKaushik, ShivaniValančiūtė, AngelijaKavroulakis, NektariosValcarce, David G.Kawabe, AkiraValdes-Lopez, OswaldoKawai, Vivian K.Valdivielso, Jose M.Kawakami, HiroshiValdiviesso, TeresaKawakami, SusumuVale, CarmenKawakami, ToshiVale, PauloKawano, KouichiroValenti, MariaKawano, MitsuokiValentová, KateřinaKawasaki, HidekiValentovic, MonicaKawase, OsamuValenzuela, RodrigoKawata, KazumiValenzuela, StellaKawauchi, TakeshiValero, ManuelKawiak, AnnaValiante, VitoKayser, KlausValis, MartinKazama, TomohikoValko, KlaraKazeto, YukinoriValko, MarianKazuaki, TaguchiValkov, VladimirKe, BiboVallat, Jean-MichelKe, Liang-YinVallée, AlexandreKe, Po-YuanVallée, YannickKe, YouqiangVallesi, AdrianaKeat Wei, LooValletta, AlessioKędziora, AnnaVallone, DanielaKeenan, JoanneVallvé, Joan-CarlesKeereetaweep, JantanaValtorta, SilviaKehl, FlorianVamanu, EmanuelKehoe, PatrickVan Beuningen, HenkKeightley, CristinaVan Beusecum, Justin P.Keisaku, SatoVan Cruchten, Steven J.Kelarakis, AntoniosVan Cutsem, PierreKelleher, Fergal C.Van Dam, PeterKellenberger, StephanVan De Ven, RienekeKeller, FriederVan Den Akker, GuusKeller-Pinter, AnikoVan Den Akker, Sebastian EvesKelley, Dior R.Van Den Ende, WimKelley, ThomasVan Den Heuvel, Ellen GHMKello, MartinVan Den Heuvel, JoopKelly, ChristopherVan Der Kamp, Jan WillemKelly, CiaránVan Der Kuyl, AntoinetteKelly, DavidVan Der Meeren, AnneKelly, DervlaVan Der Vorst, EmielKelly, RobertVan Dyke, Michael W.Kelly, William KVan Erp, PietKemp, Michael G.Van Geijlswijk, Ingeborg M.Kempisty, BartoszVan Gisbergen, Marike W.Kendig, MichaelVan Haele, MatthiasKennedy, DavidVan Hook, M.J.Kennedy, Michael A.Van Kempen, LeonKennedy, Oran DVan Laar, RyanKenneth, NiallVan Noorden, RonKępa, MałgorzataVan Rijt, SabineKepinska, MartaVan Royen, Martin E.Kerch, GarryVan Straalen, Nico M.Kerchev, PavelVan Veldhoven, PaulKerekes, GyorgyVan Waardenburg, RobertKern, JanetVan Zandwijk, NicoKerou, MelinaVan Haute, LindseyKerr, IanVance, DavidKerwin, Rachel E.Vande Velde, ChristineKesharwani, SiddharthVandersteen, AnthonyKessel, DavidVandooren, JenniferKeung, Albert J.Vanella, LucaKevadiya, BhaveshVanham, GuidoKeyel, Peter A.Vaňhara, PetrKhacho, MireilleVankayala, RavirajKhaddaj-Mallat, RayanVanneste, SteffenKhafaji, SalwanVanni, RobertaKhairullina, VeronikaVanthuyne, NicolasKhalili-Araghi, FatemehVáradi, CsabaKhaliulin, IgorVarala, KranthiKhalyfa, AbdelnabyVarchetta, StefaniaKhalymbadzha, Igor A.Varga, BalazsKhambu, BilonVarghese, MathewsKhan, Atif AliVarikuti, SanjayKhan, HamidullahVarma, Vijaykumar B.Khan, IrfanVarra, MichelaKhan, MahmoodVarricchio, EttoreKhan, Meraj AlamVartiainen, Maria K.Khan, Mohammad BadruzzamanVasani, Naresh B. Khan, Mohd ParvezVasic, VesnaKhan, MohsinVasicek, OndrejKhan, Muhammad JamalVassilakos, NikonKhanh, Tran DangVassiliou, Aliki GeorgiaKhaperskyy, Denys A.Vasudevan, RavikumarKharkar, PrathameshVathipadiekal, VinodKhasawneh, FadiVattipally, SreenuKhashayar, PatriciaVautier, SimonKhatabi, BehnamVavitsas, KonstantinosKhatri, KshitijVazquez Belda, BeatrizKhattab, MuhammadVeatch-Blohm, MEKhaw, Kooi YepngVecchione, CarmineKhazaei, HamidVecerek, BranislavKhazaie, KhashayarshaVechetti, IvanKhedoe, Padmini S.J.Veenman, LeoKhisamutdinov, EmilVeeramani, SureshKho, Kang HeeVeeranki, SudhakarKhobta, AndriyVeeravalli, VijayabhaskarKhodakovskaya, MariyaVega, Francisco M.Khodanovich, MarinaVeit, GuidoKholmukhamedov, AndalebVejux, AnneKholová, IvanaVelagapudi, RavikanthKhoo, Bee LuanVelasco, Carlos E.Khoshmanesh, KhashayarVeldhuizen, Ruud A. W.Khrustaleva, LiudmilaVeldic, MarinKhuder, Sadik A.Velegzhaninov, Ilya O.Khundmiri, Syed J.Velena, AstridaKhung, Yit LungVelez-Cruz, RenierKhurshid, ZohaibVelho, SérgiaKianifariz, MahnazVelisek, LiborKicińska, AlicjaVelizarov, SvetlozarKieber-Emmons, ThomasVellante, FedericaKiebish, MichaelVelliyagounder, KabilanKiefer, FriedemannVeltkamp, ChristianKieffer, BrunoVenditti, VincenzoKiełczykowska, MałgorzataVenkatachalem, SathishKieliszek, MarekVenkatadri, RajkumarKielland-Brandt, MortenVenkatesan, Jagadeesh KKiessling, SilkeVenkatesan, JayachandranKietzmann, ThomasVenkatesan, ThiagarajanKiger, AmyVenkatesh, SiddarthKiilgaard, Jens FolkeVenkateshwaran, MuthusubramanianKijewska, MonikaVenkateshwari, AnanthapurKikowska, MałgorzataVenter, RieteKikuchi, HidehikoVentoso, IvánKikuchi, KazuVentura-Juárez, JavierKikuta, ShingoVenturella, RobertaKikuyama, MasatakaVenturin, MarcoKilari, SreenivasuluVenugopal, DhamodharanKilaru, ArunaVerbanac, DonatellaKim, BongjuVerde, ZoraidaKim, BongleeVerdeguer, FranciscoKim, Byung SooVerdejo, BegoñaKim, Byung-GeeVerdugo-Escamilla, CritobalKim, Cheorl-HoVereda, FernandoKim, Choon YoungVerestiuc, LilianaKim, DaehwanVergallo, AndreaKim, DonginVergara, DanieleKim, Hae-WonVergés, MarcelKim, HakushiVerghese, ShilpiKim, HakwonVergine, MarziaKim, Ha-NeuiVergnaud, JulietteKim, HankiVerhage, LeonieKim, Hee SungVerheyen, EstherKim, Hee-JoonVerhoeven, AdrieKim, HoonVerhoeven, DavidKim, Ho-ShikVerhulst, AnjaKim, HyesunVerjans, Georges M.G.M.Kim, Hyun JoonVerma, AnilKim, Hyung SikVerma, NirmalKim, Hyung-JunVerma, NupurKim, In-JungVerma, RajniKim, InKyeomVerma, SureshKim, Jae KyoungVermaas, Joshua V.Kim, Jae-YoungVernetti, Lawrence A.Kim, Jee TaekVernis, LaurenceKim, Ji YeonVeronese, NicolaKim, Jin AVeronesi, FabioKim, Jin-HongVeronesi, GiuliaKim, Jin-KyungVéronique, Demers-MathieuKim, Joo-HwanVerri, TizianoKim, Jung SunVersacci, PaoloKim, Jung SungVersino, ElisabettaKim, JunhwanVerstraeten, IngeKim, Jwa-JinVertegaal, AlfredKim, Ki HyunVetere, AmedeoKim, KitaiVetvicka, VaclavKim, Kook-HyungVetvik, KatjaKim, Kyoung SooVeyron-Churlet, RomainKim, KyounghyunVezzu, DileepKim, Kyung BoViapiana, AgnieszkaKim, Kyung-MinVibhute, SandipKim, Kyung-suVicente-Manzanares, MiguelKim, KyuseokVicient, CarlosKim, MyungjinVictoni, TatianaKim, Nam DeukVictor, BrunoKim, NamshinVidetta, WalterKim, NayoungVidic, JasminaKim, Ok-SuVieira, Maria Lucia CarneiroKim, SangkyuViganò, MarcoKim, Sang-WeVígh, LászlóKim, Seok JinVíglaský, ViktorKim, Seok-HoVignes, MichelKim, Seong-RyongVignoli, AglaiaKim, Soo UnVijayakumar, SarathKim, Sung EunVijayan, MuraliKIM, Sung JoonVijayavenkataraman, SanjairajKim, Sung-HoonVikraman, DhanasekaranKim, Sung-KunVikulina, AnnaKim, Tae HyunVilahur, GemmaKim, Tae MinVilla, ChiaraKim, Tae HoonVilla-Morales, MaríaKim, Tae KonVillar-Piqué, AnnaKim, Tae-HyungVillasante, AranzazuKim, WoojinVillaseñor, AlmaKim, Yong JinVillegas, DolorsKim, Yong-GilVillegas, VictoriaKim, Yong-IckVimalachandran, D.Kim, YoonaVinall, RuthKim, YuriViñas, MiguelKimata, YukioVincent, AmyKimber-Trojnar, ŻanetaVincent, DelphineKimchi-Sarfaty, ChavaVincentini, OlimpiaKimura, AtsukoVinci, CristinaKimura, KazuhiroVinciguerra, ManlioKimura, KojiVindas, MarcoKimura, MotokoVinet, JonathanKimura, NobuyukiVingolo, Enzo MariaKimura, SeisukeVingtdeux, ValerieKimura, TomokiVining, KellyKIMURA, YoshinobuVinogradov, Alexander E.Kinoshita, HiroyukiVinothkannan, MohanrajKioussi, ChrissaViola, GiampietroKipp, MarkusViola, ManuelaKiraly, LorantVirag, Jitka A.I.Kirchmair, JohannesVirdi, Amarjit S.Kirienko, NatashaVirdi, KamaldeepKirillova, AlinaVisconti, Richard P.Kirimura, KohtaroVisentin, MicheleKirkwood, Jay SVishe, MaheshKirsch, ThorstenVisiné Rajczi, EszterKirschnek, SusanneVisone, RosaKirschner, Michaela B.Visser, LydiaKirton, Stewart B.Vissink, ArjanKiselev, AntonViswanathan, SowmyaKiselev, Konstantin V.Vita, RobertoKishaba, TomooVitale, FabrizioKishikawa, NaoyaVitale, Salvatore GiovanniKishimoto, DaigaVítámvás, PavelKishimoto, YoVitek, MichaelKiskova, TereziaVitetta, LuisKisková, TeréziaVitiello, PeterKiss, ErzsébetVitkova, VictoriaKiss, John Z.Vitorino, RuiKiss, RobertVitrac, HeidiKissinger, JessicaVitry, PaulineKitagawa, SeiichiVivacqua, AdeleKitagawa, TadaoVives-Peris, VicenteKitahara, RyoVivo, MariaKitajiri, Shin-ichiroVizoso, Francisco J.Kitamura, AkiraVizovišek, MatejKitaoka, SatoshiVlachou, MarilenaKitatani, KazuyukiVladimir, KalininKitaura, HidekiVladimirov, VladimirKitaura, JiroVladimirov, Vladimir I.Kitazawa, TakioVlahopoulos, SpirosKitchen, PhilipVliagoftis, HarissiosKitney, StuartVllasaliu, DritonKiyan, YuliaVo, Duc-ThangKiyokawa, EtsukoVo, GiauKizuka, YasuhikoVodicka, PavelKjaer, LasseVoellger, BenjaminKlampfer, LidijaVogelaar, Christina FranciscaKlar, AgnesVögele, MartinKlar, MaximilianVolarevic, VladislavKlassen, RolandVoliani, ValerioKlebba, Phillip E.Volkov, VadimKleiber, TomaszVon Ameln-Mayerhofer, AndreasKlein, David C.Von Bubnhoff, NikolasKlein, Gordon L.Von Döbeln, UlrikaKlein, MatthiasVon Karstedt, Silvia Kleinert, MaximilianVon Schacky, ClemensKleinpeter, ErichVono, RosaKlener, PavelVora, MehulKlepikova, Anna V.Vostinaru, OliviuKleppe, LeneVozzi, GiovanniKleszczyński, KonradVranic, SemirKleuser, BurkhardVriend, JerryKlimaszewski, LarsVrzal, RadimKlimczak, AleksandraVugrek, OliverKlimkowicz-Pawlas, AgnieszkaVukovic, RosemaryKlimontov, VadimVulturar, RomanaKlocko, Amy L.Vuts, JozsefKloczkowski, AndrzejVynios, DemitriosKlontzas, MichaelVyoral, DanielKlose, HolgerWabiko, HiroetsuKlughammer, JohannaWaddell, JaylynKlusa, VijaWadhwa, SunilKlymenko, YuliyaWagh, PriyeshKmiec, Eric B.Wagner, AnikaKneteman, NormanWagner, GerdKnez, DamijanWagner, Kay DietrichKnoch, EvaWahli, WalterKnoll, MarkoWakade, ChandramohanKnöll, RalphWakamatsu, KazumasaKnoop, VolkerWakao, MasahiroKnoshaug, EricWalczak, PiotrKnudsen, LarsWaldum, HelgeKo, Jane L.Walerych, DawidKo, YoungkyungWaligorski, PiotrKobae, YoshihiroWaligórski, PiotrKobayashi, Eri H.Walker, Chandler L.Kobayashi, HiroshiWalker, JessicaKobayashi, JunWalker, Robert P.Kobayashi, KazuyaWallace, David R.Kobayashi, MasaruWallace, KedraKobayashi, NobumichiWallace, MarshaKobayashi, SatoruWalldorf, UweKobayashi, TakanoriWallis, Graham P.Kobayashi, TatsuyaWallner, BjörnKobayashi, YasuhiroWalsh, KyleKobayashi, YoshiroWalsh, Kyle B.Kobayashi, YutaroWan, EdwinKobeissy, FirasWan, ShibiaoKobler, JamesWan, YinsengKobold, SebastianWanders, RonaldKocábek, TomášWang, ZhenpingKoch, WojciechWang, AilinKocsy, GáborWang, Bang-AnKoda, TomokoWang, BoKodama, DaisukeWang, Bo-ChengKodama, HiroakiWang, ChaoKoenig, XaverWang, Chrong-ReenKoepke, TysonWang, DongdongKőfalvi, AttilaWang, Eddie C.Y.Koga, FumitakaWang, Feng-ShengKoga, Jun-ichiroWang, FupengKoga, TomoyukiWang, GuanKoh, Cho YeowWang, GuankuiKoh, DavidWang, GuliangKoh, Gar YeeWang, GuozhengKoh, Ho-JinWang, HsiuyingKoh, OnoWang, Hui-minKöhl, GudrunWang, Jaw-YuanKöhler, RalfWang, JigangKöhn, MarcelWang, JingKoide, TetsuyaWang, JinxiangKoike, ShinWang, JiongweiKoivisto, AriWang, JuexinKoizuka, NobuyaWang, JunKoizume, ShiroWang, JunjieKoizumi, SchuichiWang, KaiKojima, MasamiWang, KankanKojima, TakashiWang, KunKojima-Yuasa, AkikoWang, LeiKojonazarov, Baktybek K.Wang, PeipeiKok, WouterWang, PengKokabu, ShoichiroWang, PengchengKokkinidis, MichaelWang, PiwenKolanowski, Jacek L.Wang, QingdeKolarik, Andrew J.Wang, RaymondKolbenschlag, JonasWang, Richard R.-CKolisek, MartinWang, ShapengKolk, AndreasWang, ShengKollárová, KarinWang, Shin-WeiKöller, ManfredWang, ShueKölling-Paternoga, RalfWang, ShunfangKolluru, GopiWang, ShyiwuKolodych, SergiiWang, SishuoKolosov, DennisWang, TaoKolot, MikhailWang, Tong-HongKoltai, HinanitWang, Tsung-JenKolukisaoglu, ÜnerWang, Wen-DerKomaguchi, KenjiWang, WenjingKomaki, HisayukiWang, WenqiKomarova, Natalia V.Wang, XianfengKomasa, SatoshiWang, XiaodongKomati, RajeshWang, XiaofengKomatsu, KenWang, XiaowanKomis, GeorgeWang, XueKomives, TamasWang, XueningKomiyama, TomoyoshiWang, XuewenKommireddy, VasuWang, XutongKonar, ArkaprabhaWang, YanKonc, JanezWang, YanchangKondinski, AleksandarWang, YifeiKondo, MotonariWang, YijieKones, RichardWang, YiweiKönig, GerhardWang, Yi-zhiKonigsberg, William HWang, YongqiangKoninckx, Philippe RWang, YoujinKonishi, MasaakiWang, YuKonjar, ŠpelaWang, YuanKonjevoda, PaškoWang, Yu-ChiehKonopka, AnnaWang, YuliangKonopka-Postupolska, DorotaWang, Yun-MingKonstantinidis, Theocharis G.Wang, ZhengkeKonstantinov, Spiro M.Wang, ZhihanKontogiannatos, DimitriosWang, ZhuoKontoravdi, CleoWangchuk, PhurpaKooi Yeong, KhawWankhade, Umesh D.Kopechek, JonathanWanner, MollyKopecki, ZlatkoWanrooij, PaulinaKopjar, MirelaWard, ChristopherKoppe, Janna G.Ward, Peter AKorabecny, JanWardowska, AnnaKorać, PetraWargovich, MichaelKoren, AmnonWarnes, GaryKorfei, MartinaWarrington, Junie PKorinek, VladimirWarzecha, ZygmuntKorkaya, HasanWasan, EllenKorneev, DenisWase, NishikantKornek, MiroslawWashino, SatoshiKornhuber, Malte.Washio, AyakoKornmann, MarkoWasilewska, BarbaraKorolenko, Tatyana A.Wass, MarkKorona, DagmaraWassenaar, Tsjerk A.Korsching, EberhardWasylishen, AmandaKorshunov, Maxim M.Watanabe, KazuhideKortholt, ArjanWatanabe, KazunoriKorupalli, ChiranjeeviWatanabe, MasatoshiKorzhikova-Vlakh, EvgeniaWatanabe, ShunsukeKoschella, AndreasWątek, MarzenaKosiewicz, MicheleWaterhouse, MiguelKosinsky, Robyn LauraWaters, Michael J.Koskela, ElliWaters, PaddyKoslowski, Hans RudolfWatkins, DavidKosmala, ArkadiuszWatson, AlanKosova, KlaraWaugh, DavidKostakis, IoannisWautier, Jean-LucKöster, Reinhard W.Wawruszak, AnnaKöster, WolfgangWaxman, JoshuaKostić, Aleksandar Ž.Wayel, JassemKostin, Gennadiy A.Wcisel, DustinKostner, GerhardWdowiak, KamilKostomitsopoulos, Nikolaos G.Weaver, JolantaKosuge, YasuhiroWebb, R. ClintonKosuke, NOZAKIWeber, Bernhard H.F.Kosutova, PetraWeber, DanielKoswara, AndyWeber, David S.Kot, AnnaWeber, FranzKota, SatyaWeber, GeorgKotani, TomoyaWeber, Georg F.Kotfis, KatarzynaWeber, JohnKotloski, RobertWege, Anja KathrinKotogán, AlexandraWeger, StefanKotsikorou, EvangeliaWegiel, JerzyKotta-Loizou, IolyWegner, Marthe-SusannaKotula-Balak, MalgorzataWegrzyn, AlicjaKotula-Balak, MałgorzataWehling, PeterKoturbash, IgorWei, David ChangliKoubassova, NataliaWei, Gong-HongKoukounaras, AthanasiosWei, Hong-JianKoulen, PeterWei, WanxingKouroupis, DimitriosWei, Wei-ChyouKourtidis, AntonisWei, YonglongKoutakis, PanosWei, YufengKoutelidakis, Antonios E.Weiberg, ArneKouvelis, Vassili N.Weidenhofer, JudithKouza, MaksimWeigand, Julia E.Kovacs, LaszloWeighardt, HeikeKovacs, RichardWeihua, Zhang (John)Kovács, ZsoltWeise, AnjaKovács-Öller, TamásWeiser, Douglas C.Koval, AlexeyWeiss, JuliaKoval, MichaelWeiss, RichardKovalenko, Galina A.Weisz, AlessandroKovanen, PetriWeitzel, Joachim M.Kovatcheva-Datchary, PetiaWeller, Michael G.Kowal, KrzysztofWelling, MickKowalczewski, PrzemysławWellinger, Raymund J.Kowalczuk, DorotaWellmann, SvenKowalczyk, TomaszWells, James W.Kowalska, KarolinaWelsch, RalfKowalski, Radoslaw KajetanWelsh, JaneKowol, Christian R.Welsh, MichaelKoya, DaisukeWempe, MichaelKoya, RichardWen, Amy M.Koyama, RyutaWen, ChunyiKoyama, TomotsuguWen, JiaKoyama, YuWen, JianjunKoyama, YutakaWen, Shu WenKozak, AshotWen, SijinKozaki, AkikoWen, XiaoyanKozakiewicz, AnnaWen, Yu-ChingKozempel, JánWeng, Ching FengKozhevnikova, OyunaWeng, XiaoyuKoziak, KatarzynaWenzl, RenéKozieł, EdmundWerbrouck, Stefaan P.O.Kozieł, JoannaWerley, Christopher A.Kozik, AlexanderWerner, HaimKoziorowska, AnnaWerner, Rudolf AKozlov, Andrey V.Wertz, PhilipKozlov, KonstantinWessels, IngaKozłowska (Kozlowska), MariolaWesson, DonKozlowski, LukaszWest, RachelKozlowski, Michael R.West, TimKracher, DanielWestgate, Gillian EKrähenbühl, StephanWesthauser, FabianKrajewska-Włodarczyk, MagdalenaWesthoff, Mike-AndrewKrajnak, Kristine M.Westphal, AdrieKrakowski, LeszekWestwell, AndrewKrämer, Oliver H.White, AnthonyKrammer, Eva-MariaWhitfield, PhilKranz, StefanWhitten, SteveKrasheninina, Olga A.Whittington, AndrewKrasuska, UrszulaWhyard, SteveKratky, DagmarWhyte, ClaireKratochvíl, LukášWia̧cek, Agnieszka EwaKratochwil, ClaudiusWibowo, DavidKraus, ArminWickner, Reed BKrause, GünterWidłak, WiesławaKrause, MirjaWidomska, JustynaKrebs, JoachimWie, Myung-BokKrebs, PhilippeWieczorek, EdytaKreimer, SimionWiedman, GregoryKrejbich-Trotot, PascaleWiedmann, FelixKrejčí, JanaWielbo, JerzyKrela-Kaźmierczak, IwonaWiemer, Erik A.C.Křen, VladimirWiens, DarrellKreth, JensWiernicki, IreneuszKrick, StefanieWiesner-Reinhold, MelanieKrier, FrançoisWietrzyk, JoannaKrishnamurthy, PannagaWigdahl, BrianKrishnan, HariniWiggs, MichaelKrishnan, NatrajWigle, JeffreyKrishnan, SanalkumarWijesinghe, Dayanjan S.Kristensen, MieWikel, StephenKrizbai, IstvanWiktor, ArturKrizbai, István A.Wiktorska, KatarzynaKrizek, BethWilde, H. DaytonKrol, EwelinaWildemann, BrittKroukamp, HeinrichWilk, AleksandraKroupin, PavelWilkinson, Richard D. A.Krug, SebastianWillberg-Keyriläinen, PiaKrug, Susanne M.Willcox, CarrieKrüger, MarcusWille, TimoKrugman, TamarWillerth, StephanieKruspig, BjornWilliams, Anwen SKruszewska, JoannaWilliams, E. MarkKruszewski, MarcinWilliams, IanKruyt, FrankWilliams, Stephen J.Kryger, PerWillis, RohanKrylova, Alla Yu.Willison, HughKryštof, VladimírWillmann, Matthew RKryštufek, BorisWilm, MatthiasKsiążkiewicz, MichałWilmowicz, EmiliaKu, Kang-MoWilson, Anne M.Ku, SeockmoWilson, DavidKu, Seung-YupWilson, RichardKuan, Yu-HsiangWilton, SteveKubala, SzymonWimmer, MathieuKuban-Jankowska, AlicjaWimmer, Monika A.Kubatka, PeterWimmer, ZdenekKubatzky, KatharinaWindshügel, BjörnKubiak, PiotrWinger, QuintonKubicek, ChristianWinham, Stacey J.Kubíček, VojtěchWinkler, LarsKubicova, LenkaWinter, MarnieKubinova, SarkaWinterhalter, MathiasKubiński, KonradWinuthayanon, WipaweeKubinyi, MiklósWiraja, ChristianKubo, EriWirthmueller, LennartKubo, IzumiWiśniewska, AnitaKuchay, Shafi M.Witorsch, Raphael JayKuchta, AgnieszkaWitzel, KatjaKućko, AgataWlaź, PiotrKucukkal, TugbaWłodarczyk, MaciejKucukoglu, MelisWlodawer, AlexanderKuczera, KrzysztofWłoga, DorotaKuczynska, PaulinaWnuk, MaciejKudryasheva, Nadezhda S.Wobus, ManjaKudryashov, Dmitri S.Wojciech, GiernackiKudryashova, Tatiana V.Wójcik, MaciejKuhlmann, MarkusWojtkiewicz, JoannaKuhn, ErikWojtunik-Kulesza, Karolina AnnaKuhn, HartmutWojtyczka, RobertKujan, OmarWojtyla, ŁukaszKujawa, JoannaWolfinger, Michael ThomasKujawska, MałgorzataWolfs, Tim G.A.M.Kukley, MariaWolińska, AgnieszkaKulasinghe, AruthaWołkow, PawełKulcenty, KatarzynaWolley, MartinKulda, VlastimilWon, Kyung JongKulig, DominikaWon, Moo-HoKulik, AnnaWone, BernieKulkarni, PriyankaWong, AdamKulma, AnnaWong, AlanKulminskaya, Anna A.Wong, Boon SengKulp, SamuelWong, Chi-MingKulus, DariuszWong, Ching OnKumamoto, EiichiWong, ChungKumar Jha, MithileshWong, ConnieKumar, AkhileshWong, Danny K. Y.Kumar, AnilWong, JudyKumar, BinodWong, Ka-ChunKumar, GauravWong, Ka-LeungKumar, GokhleshWong, Lee LeeKumar, JitendraWong, Michael YinKumar, ManuWong, Sek ManKumar, NarenderWong, Sek-ManKumar, NareshWoo, So-YounKumar, PavanWoo, Sun HeeKumar, PradeepWood, Andrew JamesKumar, PrasunWood, MatthewKumar, RajivWood, ScottKumar, RameshWoodman, TimKumar, SujeetWoodrow, PasqualinaKumar, SuneelWoods, Daniel F.Kumar, SunilWoolbright, Benjamin L.Kumari, AshaWooton-Kee, Clavia RuthKume, KensukeWopereis, HarmKumi-Diaka, JamesWorch, RemigiuszKumru, BarisWorley, S.D.Kunej, TanjaWosczyna, MichaelKunetsov, VladimirWozniak-Knopp, GordanaKung, Yu-ChunWree, AndreasKungolos, AthanasiosWright, CatherineKunnev, DimiterWright, HelenKunoh, TatsukiWrobel, MariaKunos, CharlesWróbel, TomaszKunsagi-Mate, SandorWu, AlanKunz, WolframWu, Alexander THKuo, Chia-HungWu, Chin-ChungKuo, Fannie CWu, ChungChunKuo, Ming-TseWu, Chung-ChunKuo, Pao-LinWu, Chung-HsinKuo, Ping-ChungWu, HannahKuo, Po-HsiuWu, HonghongKuo, Sung-HsinWu, HongliKuo, Tsung-RongWu, Hsien-TsaiKupka, TeobaldWu, Hung-ChinKuppusamy, ManiselvanWu, Jin-HuKurakula, KondababuWu, Jin-MingKurczyńska, EwaWu, JohnKurdowska, Anna K.Wu, KaiyuKurgan, LukaszWu, KushengKurganov, BorisWu, Meng-YuKurien, Biji TheyilamannilWu, QingyuKurihara, YukioWu, RongxueKurimoto, KazukiWu, Sheng-NanKuroda, MasashiWu, SiKuroda, TakuyaWu, Ting-FengKurowska, MarzenaWu, Tzong-YuanKurpisz, MaciejWu, WenruKurschus, FlorianWu, XiaohuaKurtz, IraWu, XinKuryk, LukaszWu, YadiKuryłowicz, AlinaWu, YoujunKusaczuk, MagdalenaWu, Yuh-LinKushiro, TetsuoWu, Yu-JenKusters, IljaWu, ZhihaoKustrowski, PiotrWu, ZhiqiangKusumbe, AnjaliWülfing, ClemensKuta, ElżbietaWurdak, HeikoKuter, KatarzynaWurtele, HugoKutikhin, Anton G.Wüst, Rob CIKuttel, MichelleWycisk, RyszardKutwin, MartaWyganowska-Świątkowska, MarzenaKuure, SatuWyman, IanKuwabara, TakuWymann, MatthiasKuwahara, KoichiroWystrychowski, GrzegorzKuzmanovic, LjiljanaXanthou, GeorginaKuznetsov, Vladimir V.Xaplanteri, PanagiotaKuznetsova, Irina M.Xavier Gil, JavierKuźnicki, JacekXavier, Cristina Pinto RibeiroKveberg, LiseXavier, Nuno M.Kwak, ShinXi, XinpingKweon, Oh-KyeongXi, ZhengxiongKwiecień, IwonaXia, GuangbinKwok, Hang Fai (Henry)Xia, TianKwon, Eun-YoungXia, YanKwon, InchanXian, WaKwon, Sung WonXiang, YuKwon, TaehoXiao, LiKwon, Tae-HwanXiao, QinghuanKwon, YongchanXiao, XiaKyostio-Moore, SirkkaXiao, XuKyriacou, CharalambosXiao, YuguoKyunghwan, KimXiao, ZhoushengLa Cava, AntonioXiaohan, WangLa Mendola, DiegoXiaoli, AlusLa Regina, GiuseppeXie, LishiLa Rocca, Renato V.Xie, MengLa Rosa, Valentina LuciaXie, MouzheLabbé, SimonXie, WankunLabi, VerenaXie, WenyanLabruna, GiuseppeXie, ZhiqunLabuda, MateuszXie, Zhong-RuLacerda, SaraXin, DongyueLach, Michał StefanXin, HongLacham-Kaplan, OrlyXing, GengmeiLachowicz, Joanna IzabelaXing, JunjiLachowicz, SabinaXing, MinliLacina, LukasXiong, LixiaLacko, AndrasXiong, Wen-ChengLacomme, ChristopheXiong, ZhaohuiLacza, ZsomborXu, CancanLadoux, AnnieXu, DaweiLaeger, ThomasXu, DazhongLaforenza, UmbertoXu, HuaxiLagali, PamelaXu, JianLaganà, Antonio SimoneXu, PengLagazzo, AlbertoXu, PengfeiLagendijk, Anne KarineXu, RongLaGier, MichaelXu, SuowenLagoumintzis, GeorgeXu, XuesongLahaye, MarcXu, YLahiri, AmitabhaXu, YanLahlil, RachidXu, YiLähnemann, DavidXu, YungangLahooti, HooshangXue, MeilangLahuta, Lesław BernardXue, WeiyaLai, Chin-HungXue, XiangLai, KeaneYadav, DhananjayLai, Pin-KuangYadav, MarshleenLai, SilviaYadav, Narendra SinghLai, Yu-HengYadav, PramodLaing, William AYadav, RachitaLakin, Matthew R.Yadavalli, Nataraja SekharLakin-Thomas, Patricia L.Yadgarov, LenaLall, Gurprit S.Yaeno, TakashiLallena, María JoséYagi, HidekiLaLone, Carlie A.Yagi, NaotoLam, Kwok HoYago, ToruLam, Yuen TingYaish, MahmoudLamar, John M.Yakovlev, VasilyLamarche-Vane, NathalieYamada, KenjiLamas, AlexandreYamada, SohsukeLamas, J. AntonioYamada, TaketoLamas, José RamónYamagata, KanatoLamb, DavidYamaguchi, HirokiLamba, DorianoYamaguchi, NaohiroLambing, ChristopheYamaguchi, RyujiLambrinidis, GeorgeYamaguchi, SoichiroLamichhane, PurushottamYamaji, ToshiyukiLamont, IainYamakuchi, MunekazuLamothe, ShawnYamamoto, HiroshiLampe, JedYamamoto, SuguruLan, LanYamamoto, YusukeLan, Ming-YingYamamura, ShoheiLandi, ClaudiaYamamura, SoichiroLandi, LuciaYamasaki, HideoLandi, MarcoYamasaki, MasahiroLandick, RobertYamashiro, SawakoLang, BingYamashiro, TakashiLang, DiYamashita, AkiraLang, LiweiYamashita, AtsushiLang, MichaelYamashita, HirokoLang, RolandYamauchi, AkiraLangendijk, PieterYamawaki, YosukeLanger, IngridYan, AiminLanger, RupertYan, AnLanger, ThierryYan, ChunhongLangner, CordYan, GuipingLang-Olip, IngridYan, ShengminLankeit, MareikeYan, Yong-BinLanktree, Matthew B.Yanagida, ToshioLanning, NathanYanda, MuraliLanza, GiuseppeYanez-Mo, MariaLanzillotta, MarcoYang, Chang-HaoLanzuolo, ChiaraYang, Chih-JenLaparra-Hernández, JoseYang, Chih-YuLapid, KfirYang, Chuen-MaoLappa, IliadaYang, DaQingLaranjo, MartaYang, Deng-HoLarchet, ChristianYang, DongqingLarraneta, EnekoYang, HongLarre, IsabelYang, InhoLarriba, EduardoYang, Jae WookLarson, Nicholas B.Yang, Jae-WonLarson, Steven R.Yang, JialeLaSalle, JanineYang, Jiann-JouLascaris, ErikYang, Jung-WuLasekan, John B.Yang, Kuang-YaoLasoń, WładysławYang, KunLasonder, EdwinYang, LiuLastovina, TatianaYang, QiyuanLászló, SzeredayYang, RendangLatacz, GniewomirYang, Rong-SenLatinovic, OlgaYang, Seung HwanLatouche, CamilleYang, SeunghwanLatour, RobertYang, Shao-YuLatowski, DariuszYang, Shih-ChunLattanzio, RossanoYang, Shun-ChinLattard, VirginieYang, Shun-FaLatunde-Dada, Gladys OluyemisiYang, Suh-ChingLau, AdelineYang, TaoLau, Chung-Ho EsmondYang, WancaiLau, GeeYang, Wei-hsiungLau, Kin H.Yang, WeipengLau-Cam, Cesar A.Yang, XiyanLaudadio, EmilianoYang, Yi-YuanLauersen, KyleYang, Yong-GuangLauf, PeterYang, YongkangLaufs, PatrickYang, YuchenLaur, JoanYang, Yueh-Hsun KevinLaurence, DewachterYang, YunzeLaurenti, MarcoYang, ZhiboLauretani, FulvioYang, ZhilongLauritano, DorinaYannakopoulou, KonstantinaLauro, ClotildeYannarelli, GustavoLauronen, JouniYano, HajimeLaursen, TomasYano, MasatoLava, Sebastiano A.G.Yano, ShozoLavandera, Jose LuisYao, Chao-LingLavano, AngeloYao, HiroshiLavarino, CinziaYao, JianLavdaniti, MariaYao, JiayiLavender, AndrewYao, PengLaviano, AlessandroYao, YuchengLaviola, GiovanniYap, DesmondLavogina, DarjaYarema, KevinLawen, AlfonsYaremenko, Ivan A.Lawson, CharlotteYarushkina, Natalia ILawson, VictoriaYarwood, StephenLayek, BuddhadevYasuda, KazukiLayer, Paul G.Yasuda, NinaLazado, CCYasuda, TakakoLazar, ZsofiaYasuhara, TakaoLazarev, VladimirYasuhiko, KogaLazzarato, LorettaYasukawa, KiyoshiLe Hir, RozennYasunaga, MasahiroLe Romancer, MurielYasuo, ShinobuLe Stunff, HerveYates, Nathanael J.Le, AnneYatoh, ShigeruLe, Nguyen Quoc KhanhYazawa, TakashiLea, Michael A.Yazdi, ImanLeabu, MirceaYazlovitskaya, Eugenia M.Lea-Smith, DavidYe, KeqiangLebaron, RichardYe, WenleiLebaron, SimonYe, YanqiLebeau, LucYe, ZhiweiLebedev, Vadim G.Yee, Jennifer K.Lechel, AndreYeh, Chau-TingLeclerc, EstelleYeh, Elizabeth S.Leclerc, FabriceYeh, Jan-yingLeclercq, AlexandreYeh, Kun-yunLeclercq, GuyYeh, Te-HueiLeCluyse, Edward L.Yeh, Ting-fengLecomte, PhilippeYehya, NadirLedda, CaterinaYeldandi, VijayLedda, LuigiYeliseev, AlexeiLederkremer, Gerardo Z.Yelle, Daniel J.Ledesma, Maria DoloresYen, AndrewLedo, AnaYen, Chia-HungLee, Bor-ShiunnYen, Feng-LinLee, BradyYen, Meng-ChiLee, Byong-taekYeo, SeonjuLee, Byung ChulYeo, SynLee, Chang HoonYeung, Tsz-LunLee, Chang-SooYew, P. ReneeLee, Che-HsinYi, Ae-KyungLee, Cheng-IYi, Tae HooLee, Chia-HungYi, Young-SuLee, Chia-HwaYildirim, Ali ÖnderLee, Chih-HungYilmaz, BahtiyarLee, Dae HoYilmaz, MehmetLee, Dai-SooYin, ChuntaoLee, Dong HunYin, GuoweiLee, GihyunYin, KingsleyLee, Hae-JuneYin, WenqingLee, HsinyuYing, ShengLee, Huei-JaneYip, Bon HamLee, Hye SukYip, Ping K.Lee, HyoungseokYisraeli, JoelLee, Jae WookYochum, Gregory S.Lee, Jae-HoYoder, KristineLee, JaeminYohda, MasafumiLee, JandeeYokoi, ShujiLee, Jeong SangYokose, SatoshiLee, Jetty Chung-YungYokota, TakafumiLee, Jin-KooYoneda, ToshikiLee, Jin-YongYonekura, ShinichiLee, Joo HyoungYoneshiro, TakeshiLee, KeehoonYong, JeongsikLee, KeunsubYong, Kar WeyLee, Kuo HaoYoo, Ji YoungLee, KyungjinYoo, Jung SunLee, Li-AngYoo, So YoungLee, Li-TingYoon, HyonokLee, Mei-YuehYoon, In SooLee, MinaYoon, Sung-SooLee, MingeolYorgan, TimurLee, Moo-SeungYoshida, Go J.Lee, Myung-ShinYoshida, HiderouLee, Sang GilYoshida, KatsunoriLee, SanghoonYoshida, KentaroLee, Seung JaeYoshida, MakotoLee, Seung-HongYoshida, ManabuLee, Shin-DaYoshida, NaoyasuLee, Soo ChoolYoshida, TakahiroLee, Su-JunYoshida, TakuyaLee, SuminYoshie, MikihiroLee, Sun-YoungYoshimoto, Francis K.Lee, TaesupYoshimura, KoichiLee, Tong GeonYoshino, HironoriLee, Tsung-HsienYoshioka, KeikoLee, Tzong-ShyuanYoshioka, Ken-ichiLee, VivianYoshioka, WataruLee, Wei-ChiehYou, LingyunLee, Wei-HwaYou, Min KyoungLee, Wing-KeeYou, Ren-InLee, Won-HaYou, XiaonaLee, WonhyeYoung, BryanLee, Yen ChienYoung, Howard A.Lee, Yeon SunYoung, Jason C.Lee, Yi-JangYruela, InmaculadaLee, Young HanYsebaert, LoicLee, Young-HoYssel, HansLee, Yun BinYu, FabiaoLee, Yun KyungYu, Feiqiao BrianLee, ZhenghongYu, GangLeeuw, Erik DeYu, GuoqinLefèbvre, Pierrè J.Yu, HaiLefebvre, VeroniqueYu, JiujiuLefere, SanderYu, KailiangLegartová, SoňaYu, LuLegembre, PatrickYu, Margaret K.Legendre, FlorenceYu, MinzhongLegendre, LaurentYu, QingsongLeggio, LorenzoYu, S. MichaelLegler, Daniel F.Yu, WeilingLegler, TobiasYu, WenquanLegrand, François XavierYu, WookyungLegrand, SylvainYu, XiangLehner, MałgorzataYu, XingwangLehotsky, JanYu, Yan PingLehtoranta, LaraYu, YanlinLei, WeiYu, Yong-mingLeibowitz, JulianYuan, HaiYingLeidenheimer, NancyYuan, Wei-EnLeimanis, MaraYuan, XinxuLeiphrakpam, PremilaYuan, ZhihongLeira Feijóo, YagoYuan, ZhiqinLeiser, ScottYuan, ZuanningLeisner, CourtneyYuann, Jeu-Ming P.Leitão, Jorge H.Yuba, EijiLeitner, Michael G.Yudoh, KazuoLeiva-Brondo, MiguelYue, John K.Lekka, MalgorzataYue, XuyiLel Khattabi, LailaYUEN, Karen Wing YeeLembo, GiuseppeYukl, Erik T.Lemma, SilviaYumoto, HiromichiLemmermann, Niels A WYun, ChawonLenaz, GiorgioYun, Cheol-WonLeney, Aneika C.Yun, Jun-WonLeng, XiaolingYun, MiyongLengyel-Zhand, ZsofiaYung, Hong-WaLenormand, PhilippeYung, Ken Kin LamLente, GáborYura, YoshiakiLentini, GiovanniYurchenko, EkaterinaLenzi, PaolaYushenova, IrinaLeo, Jack ChristopherYusof, Yuzaiful MdLéon, CatherineYutani, ReikoLeonard, StephenYuzbasheva, EvgeniyaLeonelli, FrancescaYves, CombarnousLeong, Max K.Zaballos, MiguelLeoni, AlbertoZąbek, AdamLeow, Melvin Khee-ShingZablotskii, VitaliiLepage, CecileZacchia, MiriamLepczynski, AdamZacchini, MassimoLepenies, BerndZadesenets, Kira S.Lephart, Edwin D.Zádor, ErnőLeporatti, StefanoZafar, MuhammadLermyte, FrederikZafar, Muhammad SohailLeshchyns’ka, IrynaZaffagnini, MirkoLesniak, WieslawaZagidullin, NaufalLesnikowski, Zbigniew J.Zago, MiriamLesov, IvanZahan, MariusLesyk, Roman B.Zahid, MalihaLetek, MichalZaidi, SaheemLeung, IvanhoeZaini, PauloLeung, KaHoZaiss, DietmarLeung, PollyZaitlin, DavidLeung, Yu minZaitsev, AlekseyLevade, ThierryZajdel, AlicjaLevanon, ErezZakhari, SamirLevanon, KerenZakharov, Alex V.Levaot, NoamZaki, Md. HasanLevesque, CelineZakrocka, IzabelaLevin, MichaelZalatnai, AttilaLevine, MindyZaleśny, GrzegorzLevitskii, SergeyZaliova, MarketaLevy, Arkene S.A.Zallot, Remi G.Levy, StevenZalubovskis, RavisLev-Yadun, SimchaZalutsky, MichaelLewczuk, BogdanZamani Meymian, NimaLewinska, AnnaZamaraewa, MariaLewis, KarenZamboni, AnitaLewis, Stephen M.Żamojć, KrzysztofLexa, MatejZamora, MònicaLeyva-Grado, VictorZampese, EnricoLezot, FredericZamponi, G. W.Lezza, Angela Maria SerenaZamyatnin, AndreyLhiaubet, Virginie LyriaZandarashvili, LevaniL’homme, LaurentZanella, IsabellaLi Calzi, SergioZanelli, Cleslei FernandoLi Volti, GiovanniZanetti, MichelaLi, Bingbing X.Zang, LiqingLi, Chia-JungZani, Bianca MariaLi, Chien-FengZanivan, SaraLi, DeyaoZannini, MariastellaLi, DongmeiZanotti, IlariaLi, FuyangZapata, CarlosLi, GenyiZapico, Sara C.Li, GuohuiZapico, Sara CasadoLi, HongchunZappaterra, MartinaLi, HuaZappulla, DavidLi, HuabinZaprutko, LucjuszLi, HuinanZaravinos, ApostolosLi, JiaxuZarrelli, ArmandoLi, JichenZaucke, FrankLi, Ka WanZavan, BarbaraLi, Kuo-BinZavattaro, ElisaLi, LeiZavattoni, MaurizioLi, LianboZavriev, Sergey K.Li, LingZawada, KatarzynaLi, MinZdanowicz, MagdalenaLi, NingZdarta, AgataLi, RenfengZdarta, JakubLi, Shau-HsuanZdeněk, KubátLi, Shengwen CalvinZdenek, SloukaLi, ShitaoZdrowowicz, MagdalenaLi, ShuaiZdziarski, PrzemyslawLi, ShurenŻebrowska-Gamdzyk, MartaLi, TianhuZecchi, SandraLi, TommyZeidán-Chuliá, FaresLi, WeihanZeidi, MahdiLi, WenboZeilinger, CarstenLi, Wen-WuŻelaszczyk, DorotaLi, WenyiZeleznik, Oana AlinaLi, Xiang-GuoZelger, BernhardLi, XinZelkó, RománaLi, XinghuiZelt, Jason G. E.Li, YinZeltner, NadjaLi, YingZeman, MichalLi, YvonneZemlyanskaya, Elena V.Li, ZhijunZendo, TakeshiLi, Zhi-yongZeng, AilanLi, ZiminZeng, HuaweiLiagre, BertrandZeng, JiaLiakopoulos, VassiliosZentner, GabrielLian, QizhouZernii, Evgeni Yu.Lian, XizhenZettler, Lawrence W.Liang, DonghaiZgórka, GrażynaLiang, Jessie QiaoyiZhai, KuiLiang, YuerongZhai, ZhiyangLiang, ZhibinZhan, YeLiang, ZhikaiZhan, YiqiangLiao, Jiahn-HaurZhang, BaozhongLiao, Jiunn-WangZhang, Can YangLiao, Jyh-feiZhang, ChangyiLiao, Pei-ChunZhang, ChengfeiLiao, PengZhang, ChiLiao, WupengZHANG, Daniel XinLiao, Yi-JenZhang, DianbaoLiao, Yung-FengZHANG, DUOLiao, YuxingZhang, FanLiapi, CharisZhang, FeiLiberale, LucaZhang, GuanshiLiberles, JessicaZhang, HengyouLibich, DavidZhang, HeyeLibra, MassimoZhang, HuLiby, KarenZhang, HuanLi-Chan, Eunice C.Y.Zhang, HuiLichocka, MałgorzataZhang, JinLichtenauer, MichaelZhang, jin-anLiddell, Jeffrey R.Zhang, JinjinLidon, FernandoZhang, JinnanLiebau, StefanZhang, KaiLiebman, MichaelZhang, KezhongLiebscher, JürgenZhang, LanLiehr, ThomasZhang, LiLigaba-Osena, AyalewZhang, Ling-juanLightfoot, AdamZhang, LiqiangLighthouse, JanetZhang, QiangzheLigi, DanielaZhang, QiupingLih-Ming, YiinZhang, RuiyongLildballe, Dorte LaunholtZhang, RunLim, Dong JunZhang, ShulingLim, EdwinZhang, WeiLim, Gah-HyunZhang, WeicaiLim, HyungyuZhang, WeijieLim, Jae HyangZhang, WenzhongLim, Ji-HongZhang, XiangLim, Kyung-MinZhang, XiaohuiLim, RoderickZhang, XiaoyuLim, Soon SungZhang, XuLim, SoyeonZhang, XuehuaLim, SungsuZhang, XuekuiLima, ErnestoZhang, XuexinLima, Rui A.Zhang, XupingLima-Brito, JoséZhang, YijunLimongi, TaniaZhang, YixiangLin, B.Y.Zhang, YixuanLin, BorenZhang, YonghongLin, Cheng-WenZhang, YongliangLin, Chia-HuaZhang, YuLin, Chien-hongZhang, ZhaoLin, Chien-MinZhang, ZhenbinLin, Chih-LiZhang, ZhuqingLin, Chi-JanZhao, BaoyinLin, Chin-YoZhao, HubinLin, HoZhao, JinpingLin, Hong-HuiZhao, MinLin, Hsu YangZhao, NingningLin, Huey-JenZhao, Peishen (Elva)Lin, Jian-MingZhao, QiLin, Jung-ChunZhao, XiaoaiLin, Kuo-ShyanZhao, XinLin, Liang-InZhao, YangbingLin, Long-LiuZhao, YuguangLin, Pei-HuiZhao, YunfengLin, SenjieZhdanova, Irina V.Lin, TaoZheng, GuopingLin, Tzu-EnZheng, QiLin, Wei-NingZheng, WenweiLin, Wei-YiZheng, XiaomingLin, WenZheng, XintingLin, YanghsiangZheng, YueLin, Yen-ChungZherdev, Anatoly V.Lin, Yen-FengZhila, Natalia OlegovnaLin, Ying-HungZhong, HaizhenLin, Yi-TingZhong, TuhuaLin, Yuan-ChiZhong, WeiLin, Yu-ChingZhong, Wei-ZhuLin, Yuh-FengZhou, BangjunLin, ZhiweiZhou, ChiLin, ZongtaoZhou, DongLindahl, LasseZhou, Donghua H.Lindberg, GerrickZhou, GuangmingLindemann, MonikaZhou, GuangqianLinder, BenediktZhou, Heshan SamLinder, StigZhou, JiajingLindkvist, KarinZhou, JingyingLindner, HeikeZhou, LinLiLindner, MartinaZhou, ManLinert, WolfgangZhou, MinLinette, Gerald PZhou, QingxiangLing, MauriceZhou, ShengbinLinhardt, RobertZhou, ShuanhuLinke, DirkZhou, WeijieLinkies, AdaZhou, WenchaoLins, LaurenceZhou, WenxuLinton, EricZhou, YiqunLiobikas, JuliusZhou, YubinLionetti, VincenzoZhou, ZhaolanLionetto, Maria GiuliaZhou, ZhichaoLiopo, AntonZhou, ZhixiongLipert, AnnaZhu, BingLipiński, Piotr F. J.Zhu, ChangfuLipstein, NoaZhu, DandanLipton, HowardZhu, DonghuiLis Arias, Manuel J.Zhu, Fang (Rose)Lisec, JanZhu, Guo-ZhangLisowska, Katarzyna M.Zhu, HongyanLister, RyanZhu, HuaListrat, AnneZhu, JieLita, AdrianZhu, JinshengLiţescu, Simona CarmenZhu, JunjieLitman, ThomasZhu, Li (Licky)Litofsky, Norman ScottZhu, LuchangLittle, JulianZhu, NaLitvinova, LarisaZhu, QifanLiu, BinZhu, QiuyuLiu, BoZhu, Qiuyu MartinLiu, Cheuk LunZhu, ShupengLiu, Chia-ChiZhu, YuLiu, Chi-MingZhu, ZhouLiu, Chung-JungZhu, ZijieLiu, DelongZhu, ZiwenLiu, FeiZhukov, IgorLiu, FuguoZhuo, ChunliuLiu, JiaŽiarovská, JanaLiu, JianZiche, MarinaLiu, Jui-MingZielenkiewicz, PiotrLiu, JunZielinska, MagdalenaLiu, Jun-JenZienkiewicz, KrzysztofLiu, JunliZienolddiny, ShanbehLiu, KangzeZierler, SusannaLiu, LiZigrino, PaolaLiu, LixinZimmer, JacquesLiu, RongmingZimmer, MarcLiu, Shing-HwaZimmer, Warren E.Liu, SongMeiZimmer-Bensch, GeraldineLiu, TianyuZimpfer, AnnetteLiu, Tzong-shiZingarelli, BasiliaLiu, XiangleiZissimou, GeorgiaLiu, XiaodongZitko, JanLiu, YanZloh, MireLiu, YangZmijewski, MichalLiu, Yen-NienZoete, VincentLiu, YiZoicas, IuliaLiu, Yi-ChangZoidis, EvangelosLiu, YingjunŻok, TomaszLiu, YonggangŻołek, TeresaLiu, YongliangŻołnowska, BeataLiu, YouzhongZordoky, BeshayLiu, Yu-PengZorec, RobertLiu, YusenZottini, MichelaLiu, ZhaohuiZou, ChunbinLiu, ZhiZou, EnminLivanos, PantelisZou, JunjieLivieris, Ioannis E.Zou, QuanLiWang, PatriciaZou, WeiLiwo, AdamZovko Končić, MarijanaLizard, GérardZsurka, GaborLjubimov, AlexanderZubarev, AndreyLlanes, CatherineZubiaga, AnaLlewellyn, CaroleZuellig, RichardLlorens, EugenioZuffo, MichelaLlorente, FranciscoZugaza, J. L.Lloret, AnaZujovic, ZoranLo Giudice, AngelinaZuluaga, PaolaLo Monte, Attilio IgnazioZumerle, SaraLo, Kai-YinZúñiga, ManuelLobaccaro, Jean-MarcZuñiga, SoniaŁobocka, MałgorzataZunke, FriederikeLoboda, AgnieszkaZuorro, AntonioLobysheva, IrinaZuther, EllenLocascio, AntonellaŽuvela, PetarLocatelli, MarcelloZvonar Pobirk, AlenkaLocatelli, VittorioZwacka, RalfLochman, JanZweiker, DavidLochmuller, HannsZych, Maria

